# Expression profiling of MADS-box gene family revealed its role in vegetative development and stem ripening in *S. spontaneum*

**DOI:** 10.1038/s41598-020-77375-6

**Published:** 2020-11-25

**Authors:** Mahpara Fatima, Xiaodan Zhang, Jishan Lin, Ping Zhou, Dong Zhou, Ray Ming

**Affiliations:** 1 College of Agriculture, FAFU and UIUC-SIB Joint Center for Genomics and Biotechnology, National Sugarcane Engineering Technology Research Center, Fujian Provincial Key Laboratory of Haixia Applied Plant Systems Biology, Fujian Agriculture and Forestry University, Fuzhou, 350002 Fujian People’s Republic of China; 2 College of Resources and Environment, Fujian Agriculture and Forestry University, Fuzhou, 350002 People’s Republic of China; 3 Department of Plant Biology, University of Illinois at Urbana-Champaign, Urbana, IL 61801 USA

**Keywords:** Genetics, Development, Gene expression, Gene regulation

## Abstract

Sugarcane is the most important sugar and biofuel crop. MADS-box genes encode transcription factors that are involved in developmental control and signal transduction in plants. Systematic analyses of MADS-box genes have been reported in many plant species, but its identification and characterization were not possible until a reference genome of autotetraploid wild type sugarcane specie, *Saccharum spontaneum* is available recently. We identified 182 MADS-box sequences in the *S. spontaneum* genome, which were annotated into 63 genes, including 6 (9.5%) genes with four alleles, 21 (33.3%) with three, 29 (46%) with two, 7 (11.1%) with one allele. Paralogs (tandem duplication and disperse duplicated) were also identified and characterized. These MADS-box genes were divided into two groups; Type-I (21 Mα, 4 Mβ, 4 Mγ) and Type-II (32 MIKCc, 2 MIKC*) through phylogenetic analysis with orthologs in *Arabidopsis* and sorghum. Structural diversity and distribution of motifs were studied in detail. Chromosomal localizations revealed that *S. spontaneum* MADS-box genes were randomly distributed across eight homologous chromosome groups. The expression profiles of these MADS-box genes were analyzed in leaves, roots, stem sections and after hormones treatment. Important alleles based on promoter analysis and expression variations were dissected. qRT-PCR analysis was performed to verify the expression pattern of pivotal *S. spontaneum* MADS-box genes and suggested that flower timing genes (*SOC1* and *SVP*) may regulate vegetative development.

## Introduction

MADS-box is one of the extensively studied transcription factor families that plays a vital role in diverse biological functions^1^. This gene family is categorized into two groups; Type-I (Mα, Mβ, Mγ) and Type-II (MIKCc and MIKC*)^2^. MADS-box family genes are characterized by the most conserved DNA binding domain, MADS-domain of about 60 amino acids in length at the N-terminal region and involved in DNA binding to their target genes based on consensus sequence CC(A/T)_6_GG (CArG box)^3^. In addition, type-II lineage contains three additional domains, (1) I-domain (intervening), for DNA-binding specificity and dimer formation, positioned between the M and K domain, (2) K-domain (keratin), characterized by coil-coil structure and responsible for protein–protein interactions and (3) C-domain (C-terminus), is the trans-activation domain and the least conserved domain among all^4^. On the other hand, MIKC*-type-II proteins have less-conserved K domain and a larger I domain as compared to MIKCc type-II proteins^5^. In *Arabidopsis*, the MIKCc proteins have been further divided into 12 subfamilies^2, 6^. Unlike type-II, type-I MADS-box proteins are simpler in structure as they lack the K domain and considered the more ancient proteins shared by plants and animals^7, 8^. In most of the plant species, type-I MADS-box genes have a faster birth and death rate than type-II genes due to weaker purifying selection and higher prevalence of segmental gene duplications^9^. To date, limited knowledge is available regarding type-I genes functions in plants.

Most of the evidences suggested that MADS-box genes are involved in many significant developmental and physiological processes, such as the gametophyte cell division, floral transition, regulation of floral organs and fruit development and ripening^10–14^. However, these genes were initially recognized as floral organ identity genes in *Arabidopsis* and antirrhinum. Modified from the original ABC model^15^, the ABCDE model that determines the identity of floral organs has been presented. Different floral organs identities are controlled by various combinations of types of genes, A + E (sepals), A + B + E (petals), B + C + E (stamens), C + E (carpels) and D + E (ovules)^16^. In *Arabidopsis,* genes belong to these functional classes were *AP1* (APETALA1) in class A, *PI* (PISTILATA) and *AP3* (APETALA3) in class B, *AG* (AGAMOUS) in class C, *STK/AGL11* (SEEDSTICK/AGAMOUS-LIKE 11) and *SHP* (SHATTERPROOF) in class D and *SEP*(SEPALLATA) genes in class E^16–18^.

Four MADS sub-families, *FLC, AGL24, SVP* and *SCO1* have been found as floral regulators. *AGL24* and *SCO1* act as floral promoters, whereas *SVP* and *FLC* functioned as flowering inhibitors^19^. To date, limited information about MADS-box transcription family is available for vegetative organs development (stem and root). Given its important roles, this gene family has been characterized in many plant species. However, no significant information is available regarding this family in important subtropical and tropical crop sugarcane.

Sugarcane (*Saccharum* spp.) is a complex polyploid (2n = 4x–16x = 32–128)^20–22^ and the most important sugar crop in the world’s economy. In the *Saccharum* complex, alot of variations in phenotypic traits exist^23, 24^. Genomic variation within *Saccharum* complex can provide understanding about polyploidization, adaptation, stress tolerance, sugar and biomass accumulation^25^. Though insight into their allelic variation has been demanding and, it is critical to identify the alleles important in controlling these traits in sugarcane. Recent generation of reference genome of wild type sugarcane, *Saccharum spontaneum* Ap85-441 (n = 4x = 32) has made it possible to explore allelic variations in MADS-box gene family^26^. *S. spontaneum* is autopolypoid with excellent stress tolerance^27^. Modern sugarcane cultivars are allopolyploids and interspecific hybrids mostly derived from crosses between domesticated *S. officinarum* and wild species *S. spontaneum*, followed by a series of backcrosses with *S. officinarum.* Sugarcane stem is the most economically important part used as raw material for sucrose and bagasse for biofuel production^28^. Although *S. spontaneum* has low biomass, but its mechanism of stem ripening is similar to that of cultivated sugarcane. In this research, we performed genome-wide identification, classification of MADS-box alleles, their structure, motif analysis, chromosome location of MADS-box genes in *S. spontaneum* and their expression profiling in vegetative organs, as well as their role in stem ripening.

## Material and methods

### Plant material

*Saccharum spontaneum* AP85-441 (n = 4x) tissues and SES208 seedlings were collected from Center for Genomics and Biotechnology, Fujian Agriculture and Forestry University, Fuzhou, Fujian 350002, China. These two *S. spontaneum* accessions were shared by Hawaii Agriculture Research Center (HARC) sugarcane genome project. No voucher specimen was deposited to a herbarium as these plant materials are readily available at HARC. Roots (R), leaves (L), stem1 (1–5 internodes; immature), stem2 (12–14 internodes; maturing) and stem3 (20–22 internodes; matured) of AP85-441 were collected, separated quickly. Further, SES-208, 35 days old seedling leaves and stems were collected after 24, 48 and 96 h. of ethylene and 48 h of auxin, gibberellin and abscisic acid treatment. Three replicates for each sample were collected and kept at − 80 °C for RNA extraction and expression analysis.

### Identification and chromosomal localization of MADS-box genes in *S. spontaneum* genome

Two methods were applied to identify MADS-box genes in the recently published *S. spontaneum* genome^26^. Firstly, HMM (Hidden Markov Model) profiles for previously submitted *Arabidopsis* MADS-box domains SRF-type-I (PF00319) and MEF2-type-II (PF09047) were obtained from the Pfam database (https://pfam.sanger.ac.uk)^29^. These profiles were used to discern MADS-box genes in the *S. spontaneum* genome by using HMMER-3.1b2 software (https://hmmer.janelia.org/)^30^. All of the proteins with an e-value lower than 0.1 were selected. Secondly, whole MADS-box proteins sequences of *Arabidopsis* and sorghum were downloaded from TAIR (https://www.arabidopsis.org/) and PlantTFDB (https://planttfdb.cbi.pku.edu.cn/) respectively. BLASTp searches were applied to predict MADS-box genes in the *S. spontaneum* genome using all *Arabidopsis* and sorghum MADS-box proteins as queries. Finally, candidate genes were examined for conserved domain by Pfam (https://Pfam.sanger.ac.uk/) and CDD (https://www.ncbi.nlm.nih.gov/cdd/) with threshold e-value < 0.0001. Molecular weight (MW) and Isoelectric point (PI) of all proteins were estimated by using ProtParam (https://web.expasy.org/protparam/). The physical location along chromosomes were determined by BLASTn searches, using all sequences as query against the *S. spontaneum* draft genome^26^. MapGene2Chrom web v2 (mg2c.iask.in/)^31^ online program was used to draw the location of *S. spontaneum* MADS-box genes on physical map of chromosomes based on their coordinates.

### Classification and subcellular localization of MADS-box genes

*Saccharum spontaneum* MADS-box members were grouped together into gene models based on the phylogenetic relationship with sorghum MADS-box genes. To further classify them into sub-groups, 102 MADS-box genes in *Arabidopsis* and 65 genes in sorghum were used as reference^2, 32^. Reference alleles of *S. spontaneum* MADS-box gene models were aligned to those of *Arabidopsis* and sorghum by MEGA7-Clustal W option^33^, and alignment results were used to construct a phylogenetic tree using maximum likelihood method with Jones–Taylor–Thornton (JTT) model. The tree was visualized by iTOL (https://itol.embl.de/) online program^34^. Subcellular localization of *S. spontaneum* MADS-box gene model’s representative alleles were predicted by WoLFPSORT (https://www.genscript.com/wolf-psort.html) and CELLO v2.5 (https://cello.life.nctu.edu.tw/).

### Comparison of *S. spontaneum* MADS-box gene structure and conserved motif with sorghum

Coding DNA sequences were used to construct an evolutionary tree for reference alleles of *S. spontaneum* MADS-box gene models together with sorghum by using Clustal W and maximum likelihood method via MEGA version 7^33^. Intron/Exon map was constructed using gff3 annotation from the published *S. spontaneum* genome assembly^26^ by Gene Structure and Display Server 2.0 (gsds.cbi.pku.edu.cn/) online program^35^.

Moreover, their protein sequences were searched for conserved motifs by MEME 5.1.0 (meme-suite.org/tools/meme) online program with the following parameters: number of motifs-20, optimum width ranges from > 6 to < 200. Motif distribution was built with TBtools software (https://github.com/CJ-Chen/TBtools). Finally, obtained motifs were annotated by using SMARTBLAST-NIH (https://blast.ncbi.nlm.nih.gov/smartblast/) online program. Furthermore, gene structure and motif distribution for all *S. spontaneum* MADS-box sequences were also obtained using the same methods.

### Transcriptome analysis

Total RNA was isolated from the collected samples using the Illumina TruSeq RNA Sample Preparation Kit (RS-122-2001(2), Illumina) following the manufacturer’s protocol. For each sample, three replicates were used. Sequencing was done by Illumina HiSeq2500 at the Center for Genomics and Biotechnology at the Fujian Agriculture and Forestry University. After that, adapter sequences were removed from raw reads using FASTX-toolkit. Sequence quality was examined by FastQC, and low-quality reads were removed. Clean reads were then mapped to the *S. spontaneum* genome^26^ by using Tophat v.2.0.10 and transcriptome assembly and DEG’s analysis were conducted using Cufflinks^36^ with parameters: p value < 0.05 and log2FC > 1 or < − 1. Furthermore transcript abundance was calculated as Fragment per Kilobase Million (FPKM) using stringtie^37^. Heat map of MADS-box genes were generated based on the FPKM values of gene models (average FPKM of all alleles and paralogs within a gene model).

### Cis-element analysis

1.5 kb sequence upstream of ATG (translation initiation site) of differentially expressed genes during stem development were extracted from *S. spontaneum* genome sequences. Different cis-elements were predicted at PlantPAN (https://plantpan.itps.ncku.edu.tw/) and PlantCARE (https://bioinformatics.psb.ugent.be/webtools/plantcare/html/) and counted. TBtools^38^ was used to plot the enrichment of these elements.

### Quantitative RT-PCR

Same RNA samples sent for RNA-seq were used for expression quantification by qRT-PCR. The first-strand cDNA was synthesized by EasyScript One-Step gDNA Removal and cDNA Synthesis Supermix. For expression quantification, primers for specific genes based on their representative alleles were designed by Primer Premier 5.0 software (Table S6). qRT-PCR was conducted using SYBR Premix Ex Taq (Tli RNaseH Plus) kit (TaKaRa, Japan), in Bio-Rad iQ5 Real-Time PCR System, with profile 95 °C for 30 s, followed by 40 cycles at 95 °C for 5 s and 60 °C for 30 s. GAPDH (glyceraldehyde-3-phosphate-dehydrogenase) gene was chosen as control for data normalization^39, 40^. For the reliability of results, three technical replicates of each sample were used. Finally, the 2^−ΔΔCt^ method was employed to compute the relative quantitative gene expression^41^.

## Results

### Identification, characterization and classification of* S. spontaneum* MADS-box genes

For identification of MADS-box genes, we used HMM (Hidden Markov Model) and BLASTp searches to explore *S. spontaneum* genome. Finally, a total of 182 non-redundant protein sequences were retained after conserved domain confirmation. The ORF length of *S. spontaneum* MADS-box sequences ranged from 174 to 2388 bp, with encoded proteins ranged from 58 to 796aa. These proteins have a predicted PI ranged from 4.4 to 11.13 and molecular weight ranged from 6479.7 to 87,287.16 KDa. (Table S1). Accession number along with sequences of these 182 non-redundant sequences are provided in Table S6.

An un-rooted tree of 182 *S. spontaneum* MADS-box sequences was constructed to determine the gene models based on 65 sorghum MADS orthologs (same as Fig. S1), as sorghum is one of the closest relative diploid genera of *Saccharum*^42^. All the sequences were grouped into 63 gene models with variable number of alleles, paralogs and tandem-duplicates. Alleles were further confirmed by alleles table provided by^26^. These models were named as Ss-subgroup with number (1–65) in relevance with sorghum orthologs. Of them, six (9.5%) gene models were having four alleles. Twenty-one gene models were found with three alleles, 29 with two and 7 with only one allele. Interestingly*, SbMADS* 2, 33, 39, 43, 46, 50, 52, 54, 55 have no homolog in *S. spontaneum* genome. While two copies of *SsSOC1-6a/b*, *SsAG/SHP*-10a/b, *SsSEP*-22a/b, *SsMα*-48a/b and three copies of *SsMβ*-64a/b/c were specific in *S. spontaneum* genome in comparison with sorghum. Summary of 63 genes models along with their corresponding alleles and paralogs are provided in Table S1.

To further clarify the nomenclature, a phylogenetic tree was constructed using representative alleles of *S. spontaneum* MADS-box gene models with *Arabidopsis* and sorghum MADS-box protein coding genes. Out of 63, 34 gene models were determined to be type-II MADS-box genes (32 MIKCc, 2 MIKC*), and 29 were confirmed as type-I (21 Mα, 4 Mβ, 4Mγ). Interestingly, we observe species-specific clustering between *S. spontaneum* and sorghum, which indicated that *S. spontaneum* is much close to sorghum as they shared a common ancestor about 8 million years ago. We also found one new cluster of 4 gene models (representative alleles *Sspon.001C0027130*, *Sspon.004A0013490, Sspon.006C0021980,Sspon.003B0025330*) with no sorghum orthologs, but very close to *Arabidopsis* Mγ sub-group and were considered as Mγ-like.

**Figure 1:**
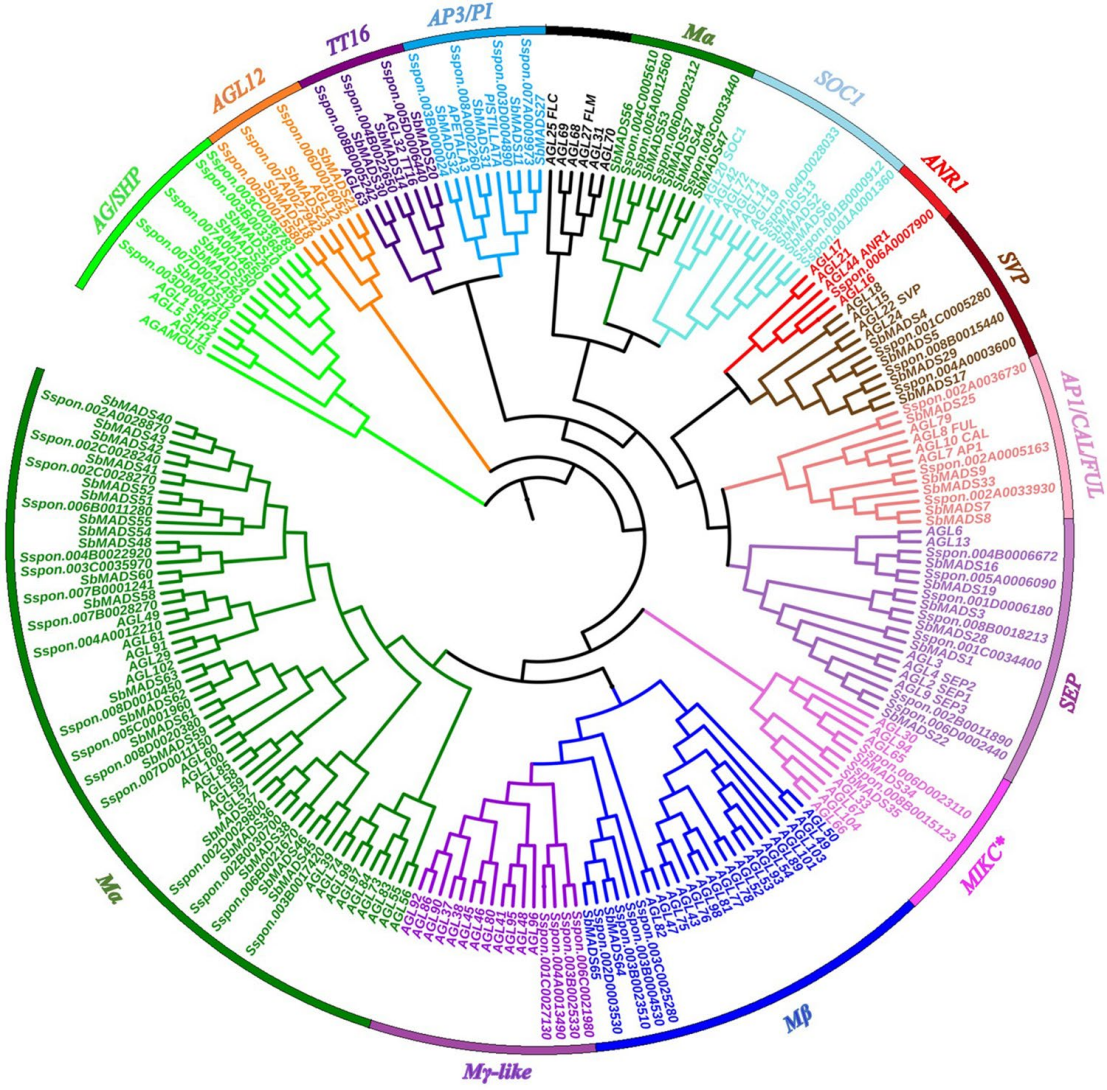
Phylogenetic relation of MADS-box genes in *S.spontaneum*, *Arabidopsis* and sorghum. The phylogenetic tree was constructed using maximum likelihood method. The full-length amino acid sequences were aligned by Clustal-W. The sub-families are presented by different colors.

Based on the phylogenetic tree analysis, 32 MIKCc were divided into 10 subfamilies. The tree shows that there were three homologs for *SVP*, *AGL12*-like, *TT16* and *SOC1*, four for *AP3/PI*, five for *AG/SHP*, seven for *SEP* and one for *ANR1*. We did not find any *FLC/FLM* subfamily members in *S. spontaneum* (Fig. 1).

### Chromosomal location of *S. spontaneum* MADS-box genes

**Figure 2:**
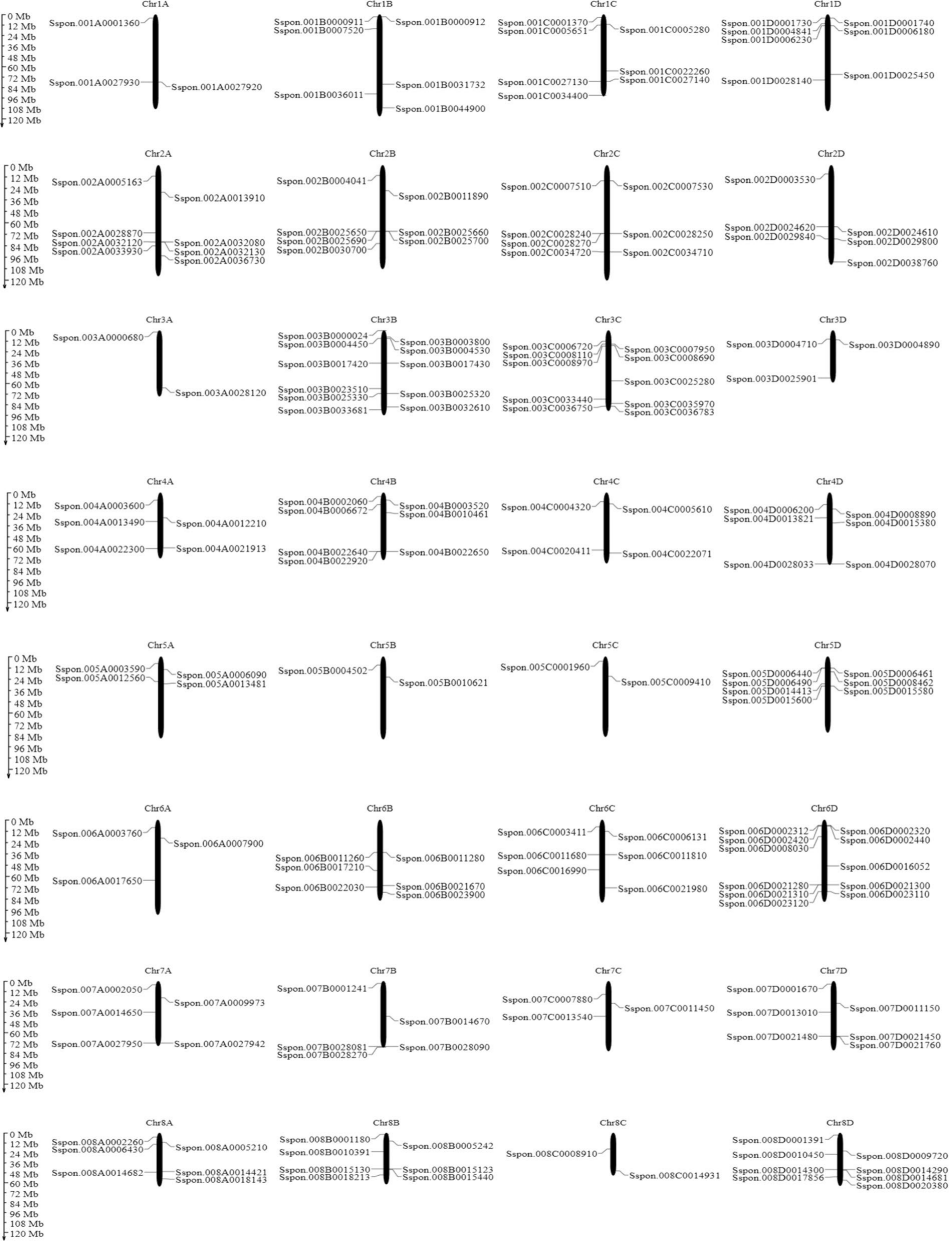
Distribution of 182 MADS-box members on homologous chromosomes.

Physical location of 63 genes (182 MADS-box sequences) were mapped onto the homologous chromosomes (Fig. 2). These sequences were randomly distributed among A 36 (~ 20%), B 51 (~ 28%), C 41 (~ 22%) and D 54 (~ 30%) homologous chromosomes, respectively.

**Figure 3:**
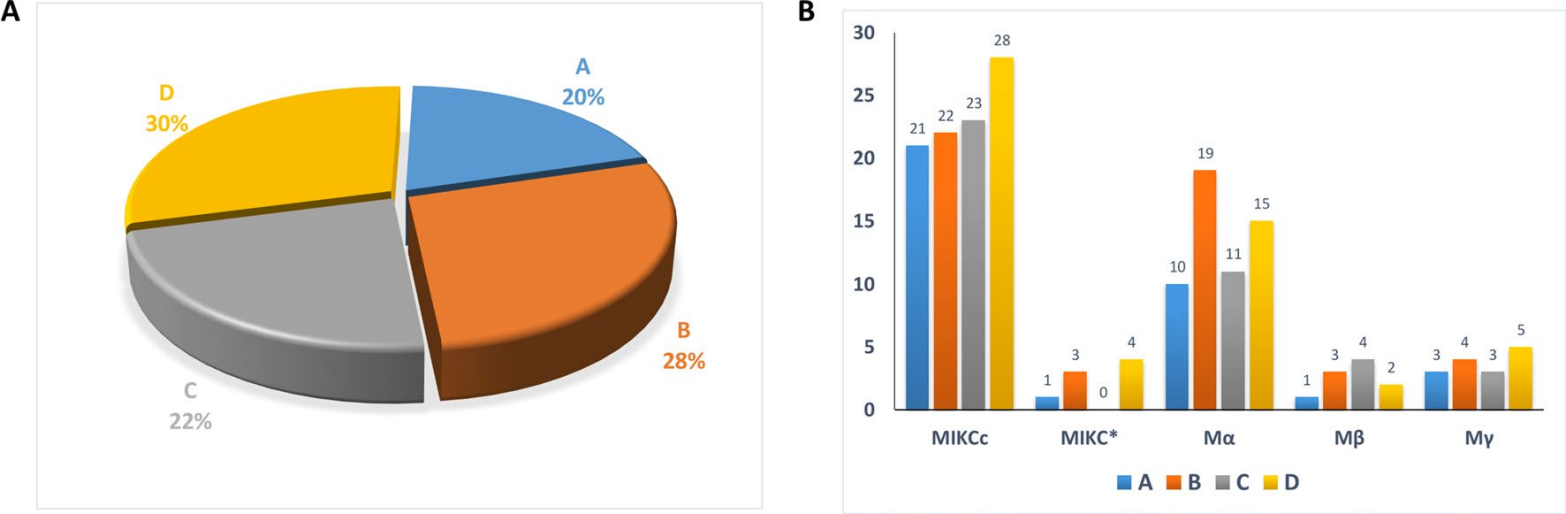
(**A**) The percentage of 182 MADS-box members anchored in four homologous groups (A, B, C, and D). (**B**) Number of different *S. spontaneum* MADS-box type genes (MIKCc, MIKC*, Mα, Mβ, Mγ) in each homologous group.

Out of 102 type-II, 32 sequences were distributed in D, while remaining were equally distributed among A(22), B(25) and C(23) homologous chromosomes. Most of these sequences were present on Chr8B (6) and Chr5D (6). Type-I were randomly distributed among A(14), B(26), C(18), and D(22) . Most of these members were mapped on Chr3B (9). Further distribution proportion of MADS-box members in *S. spontaneum genome* is shown in (Fig. 3).

### Predicted subcellular localization of *S. spontaneum* MADS-box proteins

Subcellular localization of *S. spontaneum* MADS-box proteins based on representative alleles of 63 gene models was predicted by two different tools and higher confidence prediction was mentioned in Table 1. Most of the *S. spontaneum* MADS-box proteins were predicted to be localized in nucleus, while *SsAG/SHP-*26*, SsAGL12-like*-23*, SsMβ*-65 in cytoplasm. SsMα-60, *SsMα*-63*, SsMα*-36*, SsMα*-58*, SsMα*-44, *SsMα*-57 were found in chloroplast, *SsMα*-61, *SsMα*-59, *SsMα*-38, *SsMβ*-64b and *SsMγ-like-d* in mitochondria. However, *SsMβ*-64c*, SsMγ-like-a, SsMγ-like-b* and *SsMγ-like-c* were predicted in mitochondria and chloroplast. Only one protein *SsSVP-*29 was predicted in endoplasmic reticulum. This may suggest that type-I genes may require for regulating expression in mitochondria and chloroplast compartments.

**Table 1:** Summary of MADS-box gene models in *S. spontaneum.*

Gene model	Representative allele	Exon number	Alleles	Paralogs		Subcellular localization
				Dispersaly duplicated	Tandem duplication	
*SsSOC1*_6a	*Sspon.001B0000912*	1	2	0	0	Nuclear
*SsSOC1*-6b	*Sspon.001A0001360*	8	4	0	0	Nuclear
*SsSOC1*-13	*Sspon.004D0028033*	*5*	*1*	*1*	0	Nuclear
*SsAG/SHP*-12	*Sspon.003D0004710*	7	2	0	0	Nuclear
*SsAG/SHP*-24	*Sspon.007D0021450*	7	3	0	1	Nuclear
*SsAG/SHP*-26	*Sspon.007A0014650*	6	4	0	0	Cytoplasmic
*SsAG/SHP*-10a	*Sspon.003B0033681*	8	2	0	0	Nuclear
*SsAG/SHP*-10b	*Sspon.003C0036783*	3	1	1	0	Nuclear
*SsSEP*-22a	*Sspon.006D0002440*	8	3	1	0	Nuclear
*SsSEP*-22b	*Sspon.002B0011890*	7	2	0	0	Nuclear
*SsSEP*-1	*Sspon.001C0034400*	6	2	1	0	Nuclear
*SsSEP*-3	*Sspon.001D0006180*	6	3	1	0	Nuclear
*SsSEP*-28	*Sspon.008B0018213*	7	3	0	0	Nuclear
*SsSEP*-16	*Sspon.004B0006672*	9	2	0	0	Nuclear
*SsSEP*-19	*Sspon.005A0006090*	6	3	0	0	Nuclear
*SsAP1/CAL/FUL*-7/8	*Sspon.002A0033930*	6	2	0	1	Nuclear
*SsAP1/CAL/FUL*-9	*Sspon.002A0005163*	7	2	0	0	Nuclear
*SsAP1/CAL/FUL*-25	*Sspon.002A0036730*	6	2	0	0	Nuclear
*SsSVP*-29	*Sspon.008B0015440*	6	4	0	0	Endoplasmic reticulum
*SsSVP*-17	*Sspon.004A0003600*	8	3	1	0	Nuclear
*SsSVP*-4/5	*Sspon.001C0005280*	6	3	0	0	Nuclear
*SsANR1*-15	*Sspon.006A0007900*	6	2	0	0	Nuclear
*SsAGL12-like*-21	*Sspon.006D0016052*	6	4	0	0	Nuclear
*SsAGL12-like*-18	*Sspon.005D0015580*	6	2	1	0	Nuclear
*SsAGL12-like*-23	*Sspon.007A0027942*	7	2	0	0	Cytoplasmic
*SsTT16*-14	*Sspon.004B0022650*	6	3	0	1	Nuclear
*SsTT16*-30	*Sspon.008B0005242*	8	2	0	0	Nuclear
*SsTT16*-20	*Sspon.005D0006440*	6	2	3	0	Nuclear
*SsAP3/PI*-31	*Sspon.008A0002260*	7	4	0	0	Nuclear
*SsAP3/PI-*11	*Sspon.003D0004890*	7	2	1	0	Nuclear
*SsAP3/PI*-27	*Sspon.007A0009973*	8	2	0	0	Nuclear
*SsAP3/PI*-32	*Sspon.003B0000024*	4	2	0	0	Nuclear
SsMIKC*-35	*Sspon.008B0015123*	3	3	0	2	Nuclear
SsMIKC*-34	*Sspon.006D0023110*	7	2	0	1	Nuclear
*SsMα-*49	*Sspon.004A0012210*	8	2	0	0	Nuclear
*SsMα-*56	*Sspon.004C0005610*	6	3	1	0	Nuclear
*SsMα-*53	*Sspon.005A0012560*	5	3	0	0	Nuclear
*SsMα*-40	*Sspon.002A0028870*	1	3	0	0	Nuclear
*SsMα*-42	*Sspon.002C0028240*	2	3	0	0	Nuclear
*SsMα-*41a	*Sspon.002C0028270*	1	3	1	0	Nuclear
*SsMα*-51	*Sspon.006B0011280*	2	2	2	0	Nuclear
*SsMα*-60	*Sspon.007B0001241*	1	3	0	0	Chloroplast
*SsMα*-62	*Sspon.005C0001960*	6	3	0	0	Nuclear
*SsMα*-63	*Sspon.008D0010450*	1	2	0	0	Chloroplast
*SsMα*-61	*Sspon.008D0020380*	1	1	0	0	Mitochondria
*SsMα*-59	*Sspon.007D0011150*	3	2	0	0	Mitochondria
*SsMα-*48a	*Sspon.004B0022920*	1	3	0	0	Nuclear
*SsMα*-48b	*Sspon.003C0035970*	1	3	0	0	Nuclear
*SsMα*-36	*Sspon.002B0030700*	1	3	0	0	Chloroplast
*SsMα*-37	*Sspon.002D0029800*	4	2	0	0	Nuclear
*SsMα*-38	*Sspon.006B0021670*	1	2	0	0	Mitochondria
*SsMα*-45	*Sspon.003B0017420*	1	1	0	1	Nuclear
*SsMα*-58	*Sspon.007B0028270*	2	2	0	0	Chloroplast
*SsMα*-44/47	*Sspon.003C0033440*	1	2	0	0	Chloroplast
*SsMα*-57	*Sspon.006D0002312*	3	1	0	1	Chloroplast
*SsMβ*-65	*Sspon.002D0003530*	1	2	1	0	Cytoplasmic
*SsMβ-*64a	*Sspon.003B0004530*	1	3	0	0	Nuclear
*SsMβ*-64b	*Sspon.003B0023510*	1	1	0	0	Mitochondria
*SsMβ*-64c	*Sspon.003C0025280*	1	2	1	0	Chloro/Mito
*SsMγ-like*-a	*Sspon.001C0027130*	2	4	0	2	Chloro/Mito
*SsMγ-like*-b	*Sspon.004A0013490*	1	2	0	0	Chloro/Mito
*SsMγ-like-*c	*Sspon.006C0021980*	1	3	1	1	Chloro/Mito
*SsMγ-like*-d	*Sspon.003B0025330*	1	1	0	1	Mitochondria

### Intron–exon structure and conserved motif analysis in comparison with sorghum

**Figure 4:**
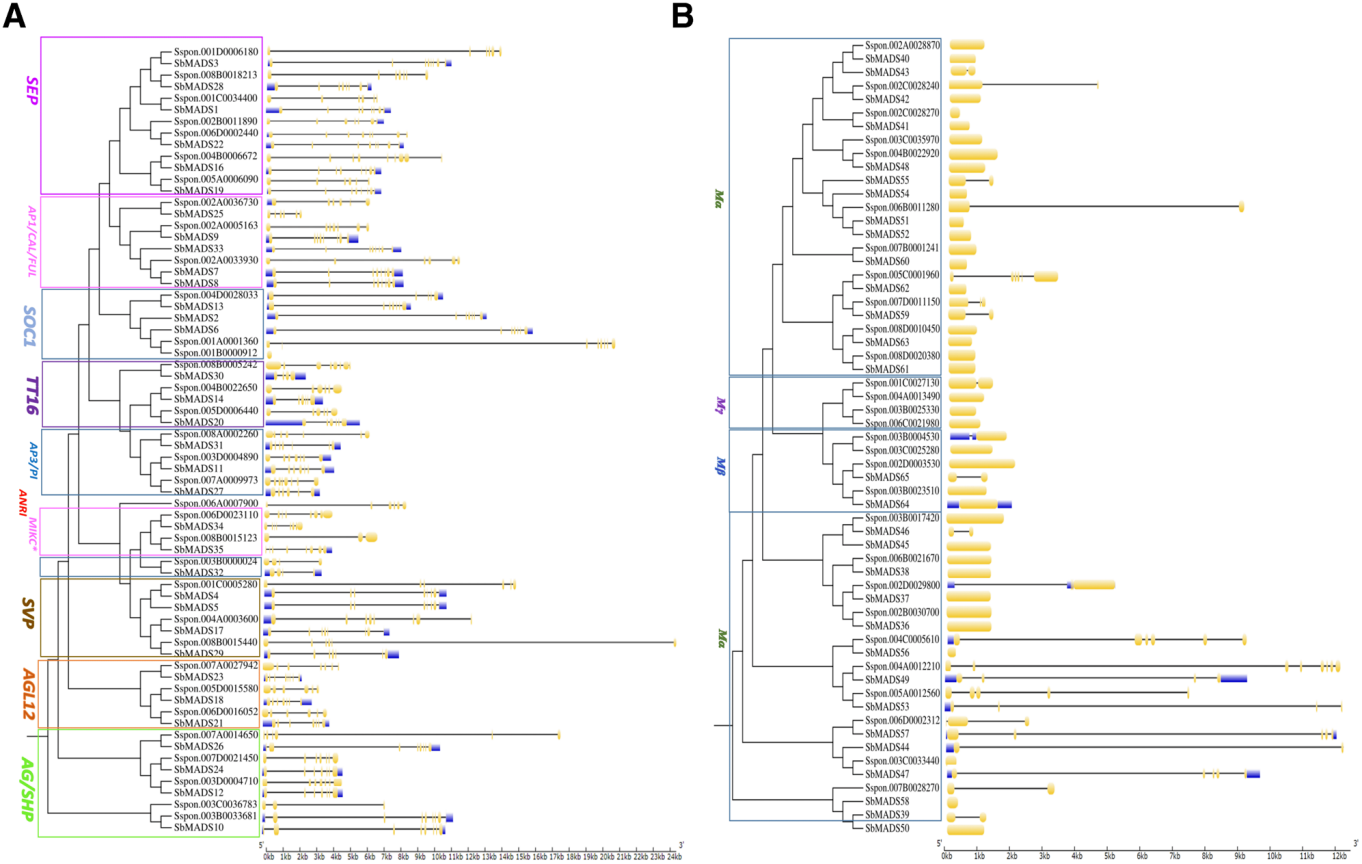
Comparative gene structure analysis of *S.spontaneum* MADS-box gene models based on representative alleles with sorghum. In exon–intron structure yellow blocks indicate exons, black lines indicate introns (**A**) Type-II MADS-box genes (**B**) Type-I MADS-box genes.

Based on the evolutionary relationship of *S. spotaneum* and sorghum, structural diversity and motif distribution were studied. When compared *S. spontaneum* MADS-box type II genes structure with sorghum, based on representative allele (Fig. 4A), it was found that both species have similar intron–exon distribution, differ only in the length of exons or introns. The same trend was observed in type-I genes (Fig. 4B) except for *SsMα*56, 49, 53 and 62, which differ in exon number.

We found that all type-I genes have simpler gene structure with exons ranged from 1 to 5, most of them have only one exon, except for *SsMα*56, 49, 53 and 62. While type-II genes have exons ranged from 1 to 14, most of type-II genes were found with 6–8 exons (Table S1). It was also found that alleles within same gene models were structurally more similar in structure.

**Figure 5:**
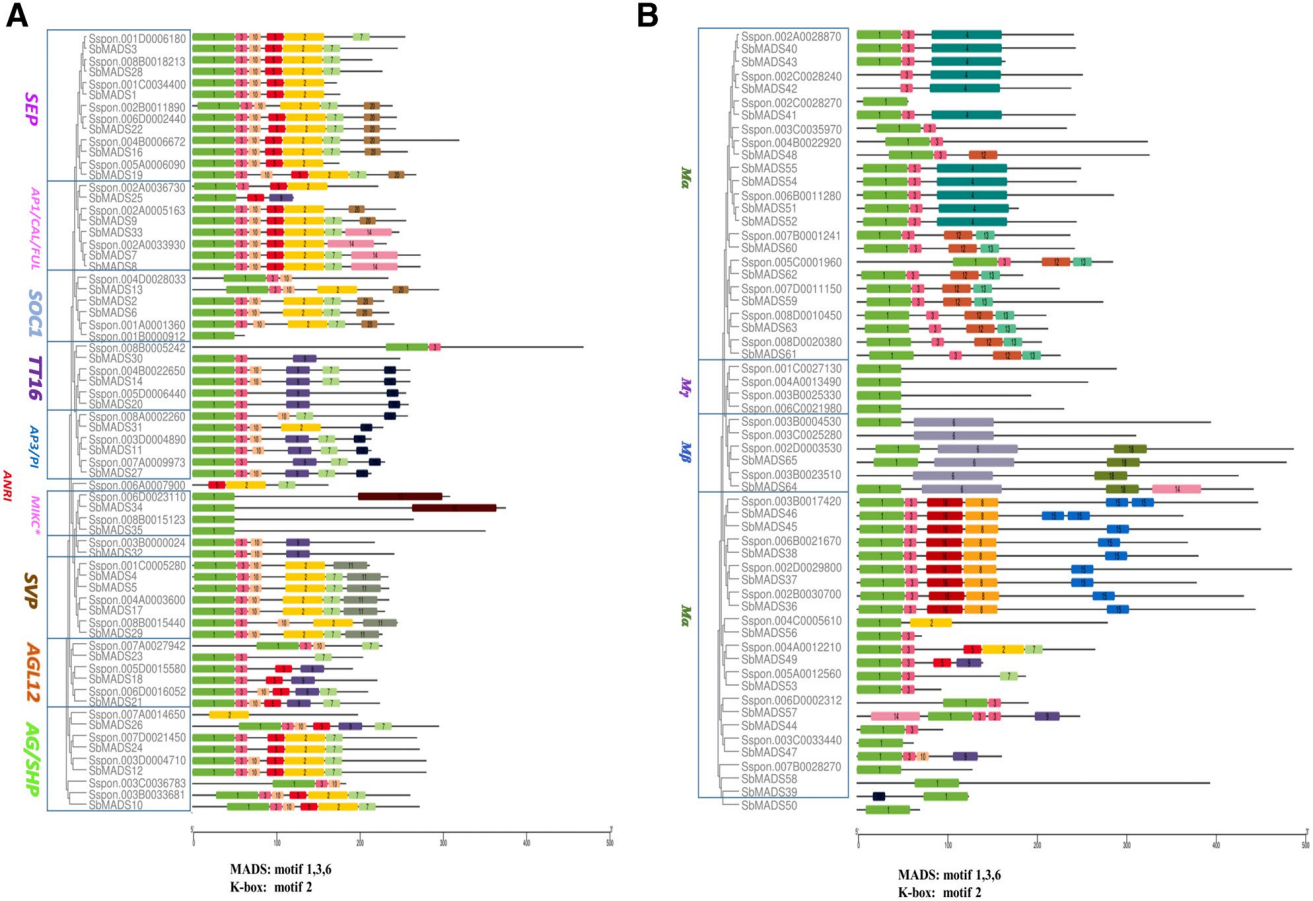
Comparative motif analysis of *S.spontaneum* MADS-box proteins based on representative alleles with sorghum. Colors represent the different motifs identified by MEME database with full-length amino acid sequences of *SsMADS.* Motif 1, 3 and 6 represents MADS-box domain and motif 2 represents K-box domain (**A**) Type-II MADS-box proteins (**B**) Type-I MADS-box proteins.

Furthermore, we obtained some essential sources of the evolutionary relationship based on similar motif distribution between *S. spontaneum* and sorghum. (Fig. 5A,B). A total of 20 conserved motifs were identified, which had previously been subjected to SMART annotation. The commonly shared motifs tended to be in the same group suggesting that the same sub-family genes probably had similar functions^2, 43^. Motif 1 was comprised of approximately 60 amino acids and was the most typical MADS-box domain. Motif 2 represented the K domain, was identified in the majority of type-II proteins. On the other hand, MIKC*-type-II proteins had a less-conserved K domain. This result is consistent with previous studies that showed that the K-box domain was only found in type-II MADS-box genes^44^. However, in our study, two type-I genes, *Sspon.004A0012210*, *Sspon.004C0005610* also contained a K-box domain. It was also found that alleles of the same gene models have similar motif distribution (Fig. S1).

### Expression Profile of* S. spontaneum* MADS-box genes in different tissues

Ripening is a continuous process in sugarcane stem as each internode is an independent growth unit. Sugarcane stem comprised a series of internodes at different physiological stages, i.e., immature (top), at maturation (middle), and mature internodes (bottom)^45^. To analyze the expression profiles of MADS-box genes, leaves, roots, stem1 (1–5 internodes: immature), stem2 (12–14 internodes: at maturation), stem3 (20–22 internodes: matured) of *S. spontaneum* variety AP85-441 were sampled and subjected to RNA-seq assays. A summary of RNA-seq mapping reads is provided in Table S2. Out of 63 MADS-box gene models, only 32 gene models showed average expression profiles (FPKM > 0) in at least one tissue tested (Table S2). For AP85, 27 (42.8%) MADS-box gene models were expressed in the leaves, 32 (50.7%) roots, 26 (41.2%) stem1, 26 (41.2%) stem2, 24 (38.03%) stem3, respectively, of which 5 (18.51%), 3 (9.3%) and 4 (15.3%), 5 (19.23%), 5 (20.8%) genes exhibited significant expression levels (FPKM value > 10), of which 1 (20%), 0 (0%), and 2 (50%), 3 (60%), 3 (60%), genes had higher expression levels (FPKM value > 30), respectively.

**Figure 6:**
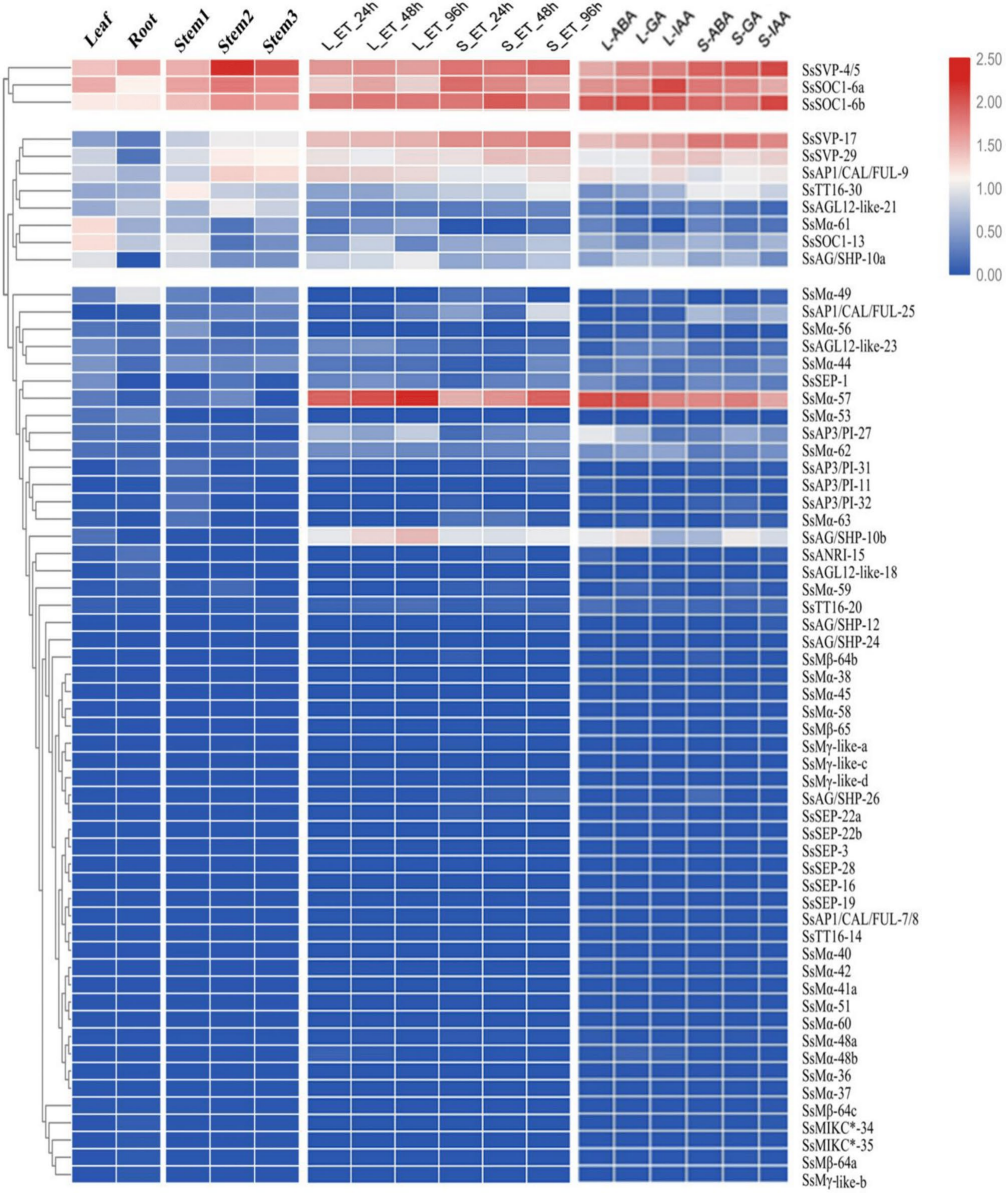
Expression pattern of *S.spontaneum* MADS-box gene models in different tissues (leaf; root; stem1, 1–5 internodes ; stem2, 12–14 internodes; stem3, 20–22 internodes), after 24 h, 48 h and 96 h of ethylene (ET) and after 48 h. of auxin (IAA), abscisic acid (ABA) and gibberellin (GA) treatment in stem and leaf. The expression value was calculated by fragments per kilobase million (FPKM). The scale represents normalized expression value (log2 (FPKM)).

The most highly expressed gene model *SsSVP-4/5*was in stem2 that have a FPKM value of 169.81. However, only three (4.34%) gene models (*SsSOC1-6a, SsSOC1-6b* and *SsSVP-4/5*) had significant transcript accumulation (FPKM value > 10) in all the five tissues tested, with higher expression in stem2 and stem3. *SsAP1/CAL/FUL-9, SVP-29*and*AGL12-like-21* were also more expressed in stem2 and stem3. These results showed that the *SOC1* and *SVP* MADS-box genes have diverse expression pattern in different tissues and more importantly in stem (Fig. 6, Table S3).

Overall, type-I gene models maintained either a relatively low transcript level or showed no expression profile in RNA-seq libraries except for *SsMα*-61. The expression of *SsMα*-61up-regulated in leaves while down-regulated in both root and stem, indicating that type-I genes are perhaps leaf-specific and have crucial role in leaf development.

### Expression profile of* S. spontaneum* MADS-box genes after hormones treatment

Ethylene plays a crucial role in sucrose accumulation and stem ripening in sugarcane. To investigate the expression patterns of MADS-box genes, two tissues leaf and stem were collected from 35 days old seedling of SES-208 after 24, 48 and 96 h of ethylene treatment. RNA-seq mapping reads summary is provided in Table S2. Nineteen MADS-box genes were expressed in at least one tissue (FPKM > 1). Out of all, *SVP* and *SOC1* gene models (*SsSVP-4/5, SsSOC1-6a, SsSOC1-6b SsSVP-17, SsSVP-29*) show higher expression in stem as compared to leaf at all time points. The most enriched expression for these genes models was observed at 48 h and 96 h. Besides, some genes, such as *SsMa57, SsAG/SHP-10a* and *SsAG/SHP-10b* were expressed much higher in leaf than in stem. Overall, lower or no expression was observed by the type-I gene in both tissues tested (Fig. 6; Table S5). The same expression pattern trend for *SVP* and *SOC1* gene models was observed after IAA, ABA, and GA treatment (48 h) (Fig. 6. Table S5) *SVP* gene models (*SsSVP*-17, 29, 4/5) showed higher expression in the stem than in leaves. While *SOC1-6b* has slightly higher expression in leaves than stem after ABA and GA treatment. *SOC1-6a* expressed more in leaves than stem after IAA treatment.

Expression pattern of *SOC1* and *SVP* genes after hormone treatment (especially ethylene) was consistent with results mentioned above in different stem sections suggested that MADS-box genes may interact directly or indirectly with hormone signal transduction genes to control stem ripening in sugarcane.

### Allelic variations in expression and cis-elements identification of *S. spontaneum* MADS-box differentially expressed genes

**Figure 7:**
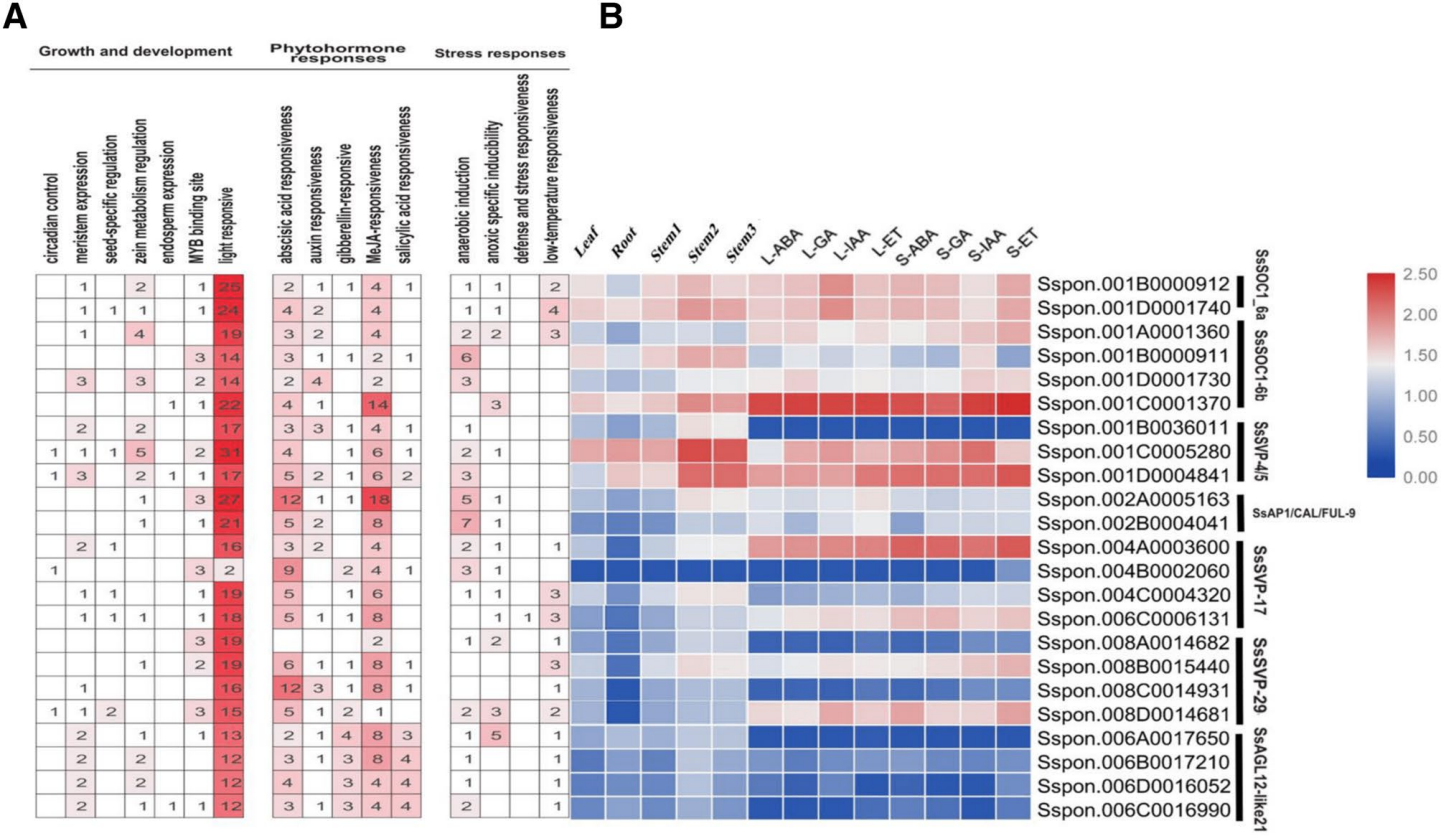
Allelic variations of *S.spontaneum* differentially expressed MADS-box gene models (**A**) It represents the different cis-elements counts, the intensity of red color shows the enrichment of cis-elements in different alleles of gene models (**B**) It represents the allelic expression pattern variations in various tissues, and after hormones treatment, (48 h). The scale represents normalized expression value (log2 (FPKM)).

The expression level of alleles of differentially expressed genes of *SVP* and *SOC1* (*SsSOC1*-6a, *SsSOC1-6b; SsSVP-4/5, SsSVP-17, SsSVP-29*) also showed differences. In view of the significance of *cis*-elements in the promoter region on gene regulation, we extracted the 1.5 kb sequence upstream to the start site (ATG) of each gene alleles to obtained cis-elements that were catagories into three groups: growth and development, stress responses, and phytohormone responses by PlantPAN^46^ and PlantCARE^47^. For growth and development, light-responsive elements G-box and Sp1 were mostly enriched *cis*-elements in these promoters (Fig. 7). *SsSVP*-4/5 alleles *Sspon.001C0005280* showed higher expression level in stem2 and 3 with a higher number (31) of light-responsive elements. However, the lowest number (2) of light responsive elements were observed in *SsSVP-*17 allele *Sspon.004B0002060* with no expression in any tissue tested.

Interestingly, it was observed that alleles with higher expression within a gene model have higher number of light-responsive elements comparing with its partner alleles, For example, *SsSOC1-*6b alleles showed different expression level (from high to low) within vegetative different stem sections tested: *Sspon.001C0001370* > *Sspon.001B0000911* > *Sspon.001D0001730* > *Sspon.001A0001360*, with 22, 14,14, 19 light-responsive elements respectively. Alleles of gene model Ss*SVP*4/5 also showed significant difference in expression; *Sspon.001C0005280* > *Sspon.001D0004841* > *Sspon.001B0036011* with 31, 17, 17 light-responsive elements, respectively. However, alleles within *SsSOC1-6a*, *SsSVP*-29 have a slight difference in expression and have an almost equal number of light-responsive elements. Moreover, the same allelic expression pattern trend was observed after hormone treatment (48 h) suggested that not all but some alleles within differentially expressed gene models are important in controlling traits in sugarcane. The distribution of hormone-related *cis*-elements was detected differently and showed in (Fig. 7). The enrichment of promoters containing light-responsive *cis*-elements in *S. spontaneum* MADS genes might not be ruled out at this point and it is an interesting question to be addressed in future research, once it is known that light-responsive element is involved in plant growth and development^48, 49^.

### Quantitative real-time PCR (qRT-PCR) analysis of differentially expressed *S. spontaneum* MADS-box genes

**Figure 8:**
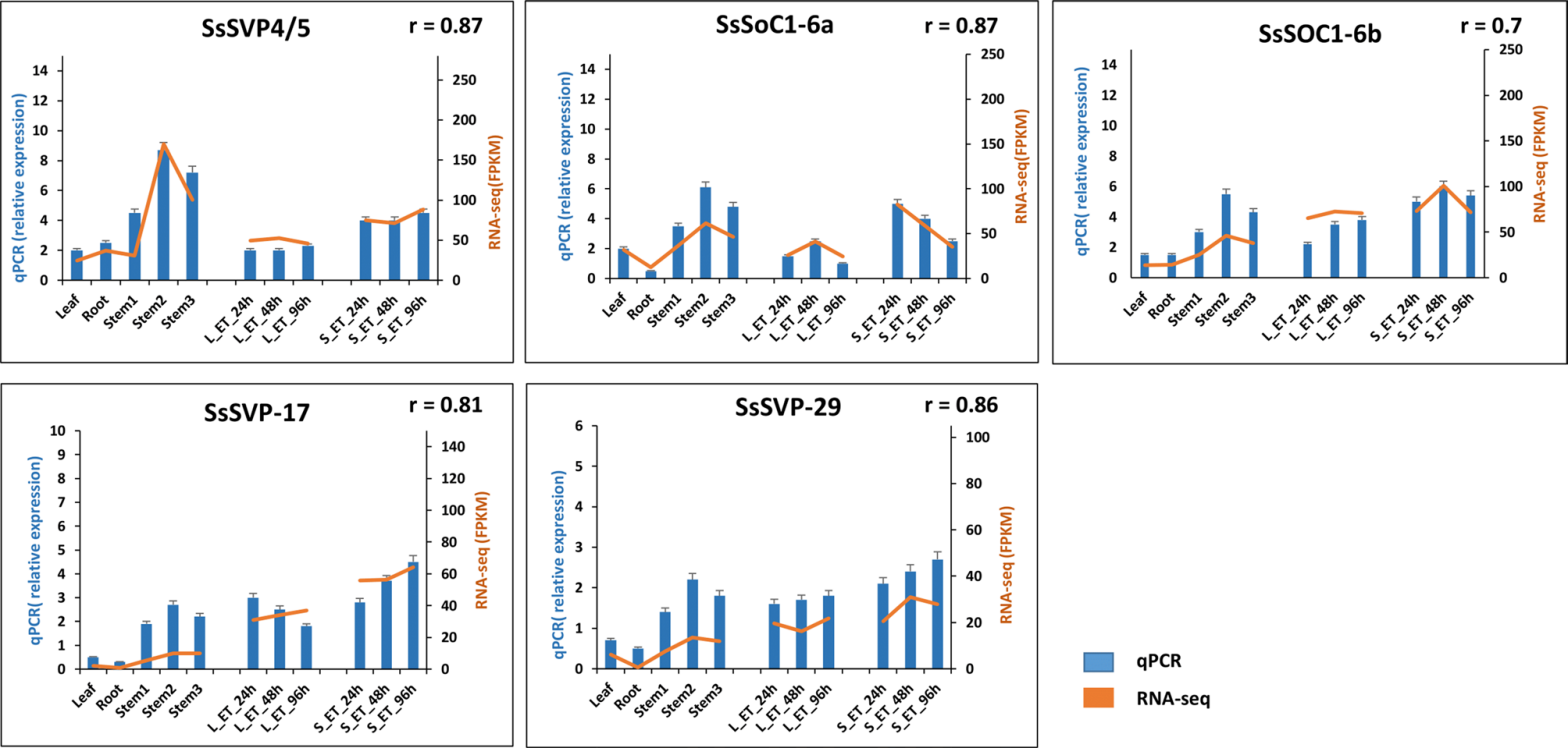
Relative expression of *S. spontaneum* MADS-box gene models by qRT-PCR. Expression pattern of *SsSOC1-6a, SsSOC1-6b, SsSVP-4/5, SsSVP-17* and *SsSVP-29* are shown in different tissues (leaf, root, stem and under ethylene treatment). Blue bars represents the relative expression in qPCR and orange line represents the expression values (FPKM) in transcriptome for corresponding genes. Correlation coefficient is also given as “r”.

According to the RNA-seq data, *S.spontaneum* MADS-box genes *SsSVP-*4/5*, SsSOC1-*6b*, SsSOC1-6a, SsSVP-*29 and *SsSVP-*17 were selected to validate the transcriptome data and subjected to qRT-PCR analysis. After normalization, we found that qRT-PCR results were consistent with the RNA-seq data for all the selected *S. spontaneum* MADS-box genes in root, leaves, different stem sections, and after ethylene treatment (Fig. 8). These results demonstrated that transcriptome data is appropriate for exploring the expression profiles of MADS-box genes in sugarcane.

## Discussion

In total, 182 MADS-box sequences were identified from the *S. spontaneum* genome. These were grouped into 63 genes, with four (6, 9.5%) three (21, 33.3%) two (29, 46%) and one (7, 11.1%) alleles. Based on phylogenetic relationships with *A. thaliana* and *S. bicolor* type-II genes, *S. spontaneum* type-II members were further classified into 10 sub-families. Interestingly, no *FLC/FLM* homologs were found in the *S. spontaneum* genome. This sub-family has been confirmed in regulating flowering time by autonomous pathways and vernalization^50, 51^. Similar results have been found in rice, sorghum, maize and cotton in the light of the fact that vernalization is not required for flowering in these species^32, 52, 53^.

The number of Introns-exon and their arrangement in gene structure, have an influence on alternative gene splicing to a certain extent with the alternation in gene function^54^. Due to structural complexity of *S. spontaneum* MADS-box type-II genes, it could be deduced that type-II genes had more complicated and variable functions comparing with type-I genes, that was in accordance with previous reports on soybean^55^, *Arabidopsis*^2^ and Chinese cabbage^56^. *S. spontaneum* MADS-box genes belonging to the same sub-families were structurally more similar with some variations in exon numbers, intron/exon length. Same trend has been observed for motif distribution in this study. Further comparison of gene structure and motif distribution of *S. spontaneum* MADS-boxgenes with sorghum highlighted the conserved evolution between these two species.

In sugarcane, ripening coincides with the process of sucrose accumulation at different physiological stages of stem^57^. In our study, expression pattern of flowering genes *SVP* and *SOC1* (*SsSOC1*-6a, *SsSOC1*-6b, *SsSVP*-4/5, *SsSVP*-17, Ss*SVP*-29*)* had significant transcript accumulation in stem 2 and 3 (maturation) indicate their diverse function in vegetative development. In banana, *SOC1, SVP* and *CAL/FUL/AP1* highly expressed during fruit developmental and ripening processes, indicating their novel roles rather than functioning on flower^58^. Recent reports on tomato and soybean suggested the fact that MADS-box genes directly regulate a set of genes involved in sugar metabolism, thereby controlling fruit ripening and root development^59, 60^. In barley, ectopic expression of *SVP*-*BM1* caused floral reversion and inhibited spike development, with florets at the base of the spike replaced by tillers^61^. While its overexpression produces chimeric floral structure bearing the typical feature of vegetative shoots so important for shoot identity^62^.

Interestingly, the same gene models of *SOC1* and *SVP* showed higher expression in stem after ethylene and other hormones treatment suggested that these gene models may involve in the hormone signal transduction pathway to regulate vegetative development. Ethylene has a substantial effect on sugarcane maturation, as exogenous application inhibits the growth of young internodes by inducing sucrose accumulation^63^. In apple, *MADS9* was found to act as a trans-activator for *ACO*1 and *ACS*1 promoters^64^. In banana, *MaMADS*7 (*AG-like*) interacted with a *MaACO*1 promoter^65^. In strawberry, *FvSOC1* regulates the differentiation of axillary buds to runners, probably through the activation of gibberellin biosynthetic genes, which plays a central role in the photoperiodic control of both generative and vegetative growth^66^. Overexpression of *KdSOC1* gene alter auxin distribution and accumulation along leaf margin to initiate plantlet formation and distribution, crucial for plasticity during plantlet formation under various environmental conditions^67^.

Furthermore, allelic variation based on expression data and promoter analysis confirms the fact that not all but some alleles within these genes are important for controlling important vegetative traits in sugarcane. These alleles are enriched with light-responsive elements involved in plant growth and development. So, it can be assumed that flower timing genes (*SVP/SOC1*), together with some other genes networks, may regulate vegetative and generative development through separate genetic pathways. The importance of these genes in vegetative development might not be ruled out at this point and it is an interesting question to be addressed in future research.

## Conclusion

Our study comprehensively analyze MADS-box genes in wildtype sugarcane, *S. spontaneum*. We identify 182 sequences that are annotated into 63 genes with variable numbers of alleles and paralogs. More importantly, some floral regulator gene models *SVP* and *SOC1* are highly expressed in stem sections, indicate their role in vegetative development. The same MADS-box genes express significantly after hormone treatment, suggesting that *SVP* and *SOC1* could be involved in the hormone signal transduction pathway to regulate stem development. Of course, the more specific functional differentiation of those genes, specifically alleles, needs further study. Genome-based identification, particularly the tissue-specific expression of these genes, provides essential information for understanding the development and transcriptional regulation of the vegetative development and stem ripening in sugarcane and may potentially aid in the understanding of the molecular basis of many agriculturally important sugarcane traits.

## Supplementary information

Supplementary Information.

## Data Availability

RNA-seq data was collected from Prof. Ray Ming and Prof. Jisen Zhang of National Sugarcane Engineering Technology Research Center, Fujian Provincial Key Laboratory of Haixia Applied Plant Systems Biology, Fujian Agriculture and Forestry University, Fuzhou, Fujian 350002, China.
