# Supplementary material for: Expression profiling of MADS-box gene family revealed its role in vegetative development and stem ripening in S. spontaneum
Source: Sci Rep. 2020 Nov 25;10:20536. doi: 10.1038/s41598-020-77375-6 (PMC7688973; doi:10.1038/s41598-020-77375-6)
Supplement: Supplementary file 1 — Supplementary Information. [file 41598_2020_77375_MOESM1_ESM.pdf]

Expression profiling of MADS-box gene family revealed its role in vegetative development and stem ripening in *S. spontaneum*

Mahpara Fatima[1], Xiaodan Zhang[3], Jishan Lin[1], Ping Zhou[1], Dong Zhou[1,2], Ray Ming[3,1]*

1. College of Agriculture, FAFU and UIUC-SIB Joint Center for Genomics and Biotechnology, National Sugarcane Engineering Technology Research Center, Fujian Provincial Key Laboratory of Haixia Applied Plant Systems Biology, Fujian Agriculture and Forestry University, Fuzhou, Fujian 350002, China.
2. College of Resources and Environment, Fujian Agriculture and Forestry University, Fuzhou 350002, PR. China.
3. Department of Plant Biology, University of Illinois at Urbana-Champaign, Urbana, IL 61801, USA

* Corresponding author; e-mail: rayming@illinois.edu

Author email: mahparafatima1@gmail.com

Table S1| Genome wide analysis of *S. spontaneum* MADS-box members

Gene Model	Locus_IDS	Sorghum _ortholog	Chromosome	Amino Acids	ORF	Exon number	PI	MW (KD)
SsSOC1_6a	*Sspon.001B0000912*	*Sb01g049020.1*	Chr1B	61	186	1	10.65	6946.17
	Sspon.001D0001740		Chr1D	61	186	1	10.65	6946.17
SsSOC1-6b	*Sspon.001A0001360*	*Sb01g049020.1*	Chr1A	238	717	8	8.74	27129.16
	Sspon.001B0000911		Chr1B	160	483	5	8.62	18598.49
	Sspon.001D0001730		Chr1D	197	594	7	5.79	22391.67
	Sspon.001C0001370		Chr1C	179	540	7	6.36	20522.61
SsSOC1-13	*Sspon.004D0028033*	*Sb04g000500.1*	Chr4D	*231*	*696*	*5*	*6.4*	25573.97
	Sspon.004D0028070		Chr4D	*223*	*672*	*2*	*5.47*	24784.17
SsAG/SHP-12	*Sspon.003D0004710*	*Sb03g042080.1*	Chr3D	276	831	7	9.23	30958.07
	Sspon.003C0007950		Chr3C	63	192	1	9.75	7265.4
SsAG/SHP-24	*Sspon.007A0027950*	*Sb08g006460.1*	Chr7A	268	807	7	9.23	30151.37
	Sspon.007B0028090		Chr7B	266	798	7	9.23	29862.03
	Sspon.007B0028081		Chr7B	122	366	4	9.95	14108.31
	Sspon.007D0021450		Chr7D	266	798	7	9.23	29848
SsAG/SHP-26	*Sspon.007A0014650*	*Sb09g006360.1*	Chr7A	165	498	6	6.9	19346.75
	Sspon.007B0014670		Chr7B	253	762	7	9.43	28847.46
	Sspon.007C0013540		Chr7C	279	837	8	9.28	31806.89
	Sspon.007D0013010		Chr7D	260	780	7	9.33	29837.63
SsAG/SHP-10a	*Sspon.003B0033681*	*Sb03g002525.1*	Chr3B	257	774	8	8.66	29050.77
	Sspon.003D0025901		Chr3D	355	1068	6	10	39246.6
SsAG/SHP-10b	*Sspon.003C0036783*	*Sb03g002525.1*	Chr3C	182	546	3	8.87	19727.41
	Sspon.003C0036750		Chr3C	197	593	4	9.72	21598.17
SsSEP-22a	*Sspon.006A0003760*	*Sb07g026200.1*	Chr6A	315	948	8	7.8	34452.92
	Sspon.006C0003411		Chr6C	174	522	6	9.22	20561.4
	Sspon.006D0002420		Chr6D	134	405	6	10.14	15843.24
	Sspon.006D0002440		Chr6D	241	726	8	8.93	28090.01
SsSEP-22b	*Sspon.002A0013910*	*Sb07g026200.1*	Chr2A	227	684	8	7.72	25886.34
	Sspon.002B0011890		Chr2B	230	692	7	7.72	26286.81
SsSEP-1	*Sspon.001B0044900*	*Sb01g007780.1*	Chr1B	241	726	8	6.97	27430.25
	Sspon.001C0022260		Chr1C	217	654	7	8.77	24726.19
	Sspon.001C0034400		Chr1C	171	513	6	9.61	19726.73

SsSEP-3	*Sspon.001B0007520*	*Sb01g042840.1*	Chr1B	207	624	8	8.42	24144.26
	Sspon.001C0005651		Chr1C	103	309	4	6.79	11906.72
	Sspon.001D0006180		Chr1D	252	756	6	8.2	28827.02
	Sspon.001D0006230		Chr1D	180	543	6	7.77	20344.11
SsSEP-28	*Sspon.008A0018143*	*Sb10g004390.1*	Chr8A	223	672	8	8.53	25569.04
	Sspon.008B0018213		Chr8B	213	639	7	8.23	24509.9
	Sspon.008D0017856		Chr8D	219	660	8	8.74	25137.64
SsSEP-16	*Sspon.004B0006672*	*Sb04g031750.1*	Chr4B	315	948	9	8.05	35913.74
	Sspon.004D0008890		Chr4D	220	660	7	9.07	25213.75
SsSEP-19	*Sspon.005A0006090*	*Sb06g026300.1*	Chr5A	173	522	6	9.72	20304.4
	Sspon.005D0008462		Chr5D	299	900	9	8.88	33531.99
	Sspon.005B0004502		Chr5B	90	273	1	10.18	10367.16
SsAP1/CAL/FUL-7/8	*Sspon.002A0033930*	*Sb02g001090.1/ Sb02g029310.1*	Chr2A	230	690	6	9.41	26348.93
	Sspon.002C0034720		Chr2C	325	978	7	9.36	37004.35
	Sspon.002C0034710		Chr2C	148	444	4	9.49	17020.49
SsAP1/CAL/FUL-9	*Sspon.002A0005163*	*Sb02g038780.1*	Chr2A	240	723	7	8.37	27127.81
	Sspon.002B0004041		Chr2B	191	576	5	8.58	22125.37
SsAP1/CAL/FUL-25	*Sspon.002A0036730*	*Sb08g015555.1*	Chr2A	219	660	6	8.74	25191.6
	Sspon.002D0038760		Chr2D	171	516	7	4.99	19474.81
SsSVP-29	*Sspon.008A0014682*	*Sb10g007380.1*	Chr8A	157	474	5	9.55	17894.36
	Sspon.008B0015440		Chr8B	243	729	6	6.56	26965.35
	Sspon.008C0014931		Chr8C	157	474	5	9.55	17924.38
	Sspon.008D0014681		Chr8D	128	387	6	6.43	14576.36
SsSVP-17	*Sspon.004A0003600*	*Sb04g033930.1*	Chr4A	232	699	8	5.4	26000.15
	Sspon.004B0002060		Chr4B	107	324	6	5.24	12578.13
	Sspon.004C0004320		Chr4C	297	894	10	7.07	33215.74
	Sspon.006C0006131		Chr6C	169	510	5	6.34	19281.95
SsSVP-4/5	*Sspon.001B0036011*	*Sb01g044810.1/ Sb01g044810.3*	Chr1B	87	261	2	7.72	9400.51
	Sspon.001C0005280		Chr1C	209	630	6	7.71	23674.8
	Sspon.001D0004841		Chr1D	275	825	8	8.22	31051.01
SsANR1-15	*Sspon.006A0007900*	*Sb04g028960.1*	Chr6A	161	483	6	5.97	19011.28
	Sspon.006D0008030		Chr6D	181	543	5	9.24	20761.61
SsAGL12-like21	*Sspon.006A0017650*		Chr6A	148	444	4	9.12	17014.64

	Sspon.006B0017210	*Sb07g001250.1*	Chr6B	403	1212	6	6.04	44333.04
	Sspon.006D0016052		Chr6D	208	624	6	6.23	23441.9
	Sspon.006C0016990		Chr6C	155	465	5	8.87	17764.42
SsAGL12-like-18	*Sspon.005C0009410*	*Sb06g017660.1*	Chr5C	348	1047	10	8.9	38900.57
	Sspon.005D0015580		Chr5D	190	570	6	8.59	22011.71
	Sspon.005D0015600		Chr5D	142	426	4	8.95	16433.91
SsAGL12-like-23	*Sspon.007A0027942*	*Sb08g006430.1*	Chr7A	225	675	7	10.32	25232.71
	Sspon.007D0021480		Chr7D	214	642	6	10.27	24019.28
SsTT16-14	*Sspon.004A0021913*	*Sb04g004736.1*	Chr4A	239	717	5	7.7	26387.9
	Sspon.004B0022640		Chr4B	258	774	6	6.45	28874.74
	Sspon.004B0022650		Chr4B	258	774	6	6.45	28874.74
	Sspon.004C0022071		Chr4C	258	774	6	6.45	28874.74
SsTT16-30	*Sspon.008A0005210*	*Sb10g026690.1*	Chr8A	320	960	5	7.02	36509.76
	Sspon.008B0005242		Chr8B	462	1389	8	5.46	51151.97
SsTT16-20	*Sspon.005A0003590*	*Sb06g028420.1*	Chr5A	631	1893	14	9.12	69259.78
	Sspon.005A0013481		Chr5A	263	792	6	9.2	30293.77
	Sspon.005D0006440		Chr5D	253	759	6	8.85	29097.31
	Sspon.005D0006461		Chr5D	239	717	5	9.07	27333.29
	Sspon.005D0006490		Chr5D	82	249	1	10.11	9530.23
SsAP3/PI-31	*Sspon.008A0002260*	*Sb10g029810.1*	Chr8A	254	765	7	7.61	28610.79
	Sspon.008B0001180		Chr8B	399	1197	2	9.4	44454.65
	Sspon.008D0001391		Chr8D	221	666	7	8.43	25181.48
	Sspon.008C0008910		Chr8C	260	783	6	8.35	29366.43
SsAP3/PI-11	*Sspon.003D0004890*	*Sb03g041860.1*	Chr3D	211	636	7	7.76	24338.82
	Sspon.003C0008110		Chr3C	211	636	7	7.76	24338.82
	Sspon.003C0008690		Chr3C	211	636	7	7.76	24338.82
SsAP3/PI-27	*Sspon.007A0009973*	*Sb09g020770.1*	Chr7A	227	684	8	9.22	26467.26
	Sspon.007C0007880		Chr7C	180	543	6	9.54	21115.13
SsAP3/PI-32	*Sspon.003B0000024*	*Sb03g033380.1*	Chr3B	215	648	4	5.77	24698.89
	Sspon.003C0008970		Chr3C	212	636	4	5.93	24232.44
SsMIKC*-35	*Sspon.008A0014421*	*Sb10g007810.1*	Chr8A	259	777	3	4.94	27939.37
	Sspon.008B0015123		Chr8B	262	786	3	4.94	28336.8
	Sspon.008D0014290		Chr8D	397	1194	10	5.39	44199.86

	Sspon.008B0015130		Chr8B	369	1110	11	5.37	40544.49
	Sspon.008D0014300		Chr8D	380	1140	10	5.09	41911.35
SsMIKC*-34	*Sspon.006B0023900*	*Sb05g025970.1*	Chr6B	307	921	7	6.15	33650.84
	Sspon.006D0023120		Chr6D	307	921	7	6.26	33694.85
	Sspon.006D0023110		Chr6D	305	915	7	6.49	33484.71
SsMα-53	*Sspon.005A0012560*	*Sb06g019040.1*	Chr5A	189	570	5	5.17	20373.36
	Sspon.005B0010621		Chr5B	87	261	2	10	9869.36
	Sspon.005D0014413		Chr5D	103	309	3	9.95	11604.48
SsMα-49	*Sspon.004A0012210*	*Sb04g024010.1*	Chr4A	268	804	8	8.39	30830.91
	Sspon.004D0013821		Chr4D	124	372	4	5.38	14087.75
SsMα-56	*Sspon.004B0010461*	*Sb07g021110.1*	Chr4B	228	687	5	9.59	26054.91
	Sspon.004B0003520		Chr4B	313	942	11	6.53	34560.28
	Sspon.004C0005610		Chr4C	281	846	6	9.45	31645.36
	Sspon.004D0006200		Chr4D	205	615	6	9.51	23663.08
SsMα-40	*Sspon.002A0028870*	*Sb02g006700.1*	Chr2A	244	732	1	5.14	26925.32
	Sspon.002B0025690		Chr2B	249	750	1	5.08	27835.49
	Sspon.002C0028250		Chr2C	194	582	1	5.58	21681.68
SsMα-42	*Sspon.002B0025650*	*Sb02g006730.1*	Chr2B	253	762	2	4.44	27926.21
	Sspon.002C0028240		Chr2C	253	762	2	4.4	27966.24
	Sspon.002D0024610		Chr2D	335	1008	5	4.47	37040.42
SsMα-41a	*Sspon.002B0025700*	*Sb02g006710.1*	Chr2B	326	981	4	5.37	35981.62
	Sspon.002B0025660		Chr2B	572	1719	6	6.15	62233.09
	Sspon.002C0028270		Chr2C	58	174	1	10.45	6479.7
	Sspon.002D0024620		Chr2D	253	762	1	5.69	27670.03
SsMα-51	*Sspon.006B0011280*	*Sb0836s002010.1*	Chr6B	289	867	2	9.46	32006.17
	Sspon.006B0011260		Chr6B	247	744	1	8.93	26892.68
	Sspon.006C0011810		Chr6C	245	738	1	8.93	26675.36
	Sspon.006C0011680		Chr6C	196	591	3	9.1	22121.3
SsMα-60	*Sspon.007A0002050*	*Sb09g029120.1*	Chr7A	237	714	1	6.53	24476.5
	Sspon.007B0001241		Chr7B	239	720	1	6.53	24652.69
	Sspon.007D0001670		Chr7D	241	726	2	6.53	24907.95
SsMα-62	*Sspon.008B0010391*	*Sb10g021663.1*	Chr8B	684	2055	8	8.93	75966.46
	Sspon.008D0009720		Chr8D	400	1203	7	9.64	45429.33

	Sspon.005C0001960		Chr5C	287	864	6	9.4	32041.25
SsMα-63	*Sspon.008A0006430*	*Sb10g019770.1*	Chr8A	203	609	1	9.81	22311.69
	Sspon.008D0010450		Chr8D	212	639	1	9.81	23327.9
SsMα-61	*Sspon.008D0020380*	*Sb10g002680.1*	Chr8D	208	627	1	9.94	22703.92
SsMα-59	*Sspon.007C0011450*	*Sb09g016220.1*	Chr7C	136	411	1	10.73	14879.2
	Sspon.007D0011150		Chr7D	227	684	3	10.64	24733.38
SsMα-48a	*Sspon.004A0022300*	*Sb04g004410.1*	Chr4A	260	783	2	9.18	27127.69
	Sspon.004B0022920		Chr4B	326	981	1	5.5	34231.3
	Sspon.004C0020411		Chr4C	328	987	1	5.42	34392.57
SsMα-48b	*Sspon.003A0028120*	*Sb04g004410.1*	Chr3A	796	2388	4	10.89	87287.16
	Sspon.003B0032610		Chr3B	150	450	1	9.89	16209.77
	Sspon.003C0035970		Chr3C	235	708	1	8.22	25439.08
SsMα-36	*Sspon.002A0032130*	*Sb02g002400.1*	Chr2A	444	1335	1	5.75	48249.39
	Sspon.002B0030700		Chr2B	434	1305	1	5.92	47054.91
	Sspon.002D0029840		Chr2D	434	1305	1	5.87	47098.93
SsMα-37	*Sspon.002A0032120*	*Sb02g002410.1*	Chr2A	448	1347	3	5.79	50242.77
	Sspon.002D0029800		Chr2D	489	1470	4	5.25	54251.17
SsMα-38	*Sspon.002A0032080*	*Sb02g002430.1*	Chr2A	199	600	2	7.72	22273.23
	Sspon.006B0021670		Chr6B	372	1116	1	9.25	41334.9
SsMα-45	*Sspon.003B0017430*	*Sb03g027570.1*	Chr3B	399	1197	2	6.93	43712.7
	Sspon.003B0017420		Chr3B	451	1356	1	6.11	49324.92
SsMα-58	*Sspon.007B0028270*	*Sb08g005892.1*	Chr7B	130	393	2	9.79	14902.67
	Sspon.007D0021760		Chr7D	72	219	2	9.85	8706.12
SsMα-44/47	*Sspon.003C0033440*	*Sb03g006210.1*	Chr3C	63	192	1	11.13	7344.63
	Sspon.003B0003800		Chr3B	203	612	1	10.29	22241.79
SsMα-57	*Sspon.006D0002312*	*Sb07g026180.1*	Chr6D	193	579	3	10.06	21920.25
	Sspon.006D0002320		Chr6D	112	339	2	10	13171.95
SsMβ-65	*Sspon.002D0003530*	*Sb02g037430.1*	Chr2D	490	1473	1	5.31	51738.17

	Sspon.002C0007530		Chr2C	492	1479	1	5.5	51709.19
	Sspon.002C0007510		Chr2C	189	567	2	10.91	20045.1
SsMβ-64a	*Sspon.003A0000680*	*Sb03g013650.1*	Chr3A	479	1440	1	4.81	51305.97
	Sspon.003B0004530		Chr3B	398	1197	1	4.83	42526.82
	Sspon.001D0025450		Chr1D	432	1299	1	4.98	46356.44
SsMβ-64b	*Sspon.003B0023510*	*Sb03g013650.1*	Chr3B	428	1287	1	4.72	45395.4
SsMβ-64c	*Sspon.003B0004450*	*Sb03g013650.1*	Chr3B	313	942	1	4.7	33662.43
	Sspon.003C0025280		Chr3C	313	942	1	4.7	33662.43
	Sspon.003C0006720		Chr3C	320	963	1	4.7	34280.09
SsMγ-like-a	*Sspon.001A0027920*	*Mγ-like*	Chr1A	305	918	1	8.73	32448.11
	Sspon.001B0031732		Chr1B	568	1704	2	6.36	60034.3
	Sspon.001C0027130		Chr1C	291	876	2	9.05	30941.3
	Sspon.001D0028140		Chr1D	305	918	1	8.73	32385.02
	Sspon.001A0027930		Chr1A	330	993	3	9.4	35554.82
	Sspon.001C0027140		Chr1C	294	882	1	8.41	31498.87
SsMγ-like-b	*Sspon.004A0013490*	*Mγ-like*	Chr4A	259	780	1	9.11	28622.14
	Sspon.004D0015380		Chr4D	265	798	1	8.93	29094.59
SsMγ-like-c	*Sspon.006B0022030*	*Mγ-like*	Chr6B	220	660	1	9.41	25104.03
	Sspon.006D0021280		Chr6D	246	741	5	9.6	28219.93
	Sspon.006D0021300		Chr6D	231	696	2	9.85	26398.68
	Sspon.006D0021310		Chr6D	446	1341	8	7.14	49720.58
	Sspon.006C0021980		Chr6C	233	699	1	9.52	26347.21
SsMγ-like-d	*Sspon.003B0025330*	*Mγ-like*	Chr3B	196	591	1	9.49	22495.12
	Sspon.003B0025320		Chr3B	297	894	3	9.79	33846

Table S2| A summary of RNA-seq mapping reads

Samples	Clean Reads	Multiple mapped reads	Uniquely mapped reads	Mapped reads %
Leaf	196911564	135135387	24671860	82.78%
Root	166132789	114012698	18242031	81.20%
Stem1	154094208	105750927	19669672	83.04%
Stem2	133966730	91937952	10507194	78.92%
Stem3	161743219	111000248	14271460	79.91%
L_ET_24h	181472403	124539884	23128835	82.33%
L_ET_48h	141996928	97448872	15313394	81.43%
L_ET_96h	143285065	98332887	9833288	77.96%
S_ET_24h	172798142	113504662	14586196	75.61%
S_ET_48h	177541163	116620175	24925386	79.32%
S_ET_96h	202106594	132756292	10343102	72.22%
L-ABA	186798927	128195342	24210606	83.21%
L-GA	166883650	114527995	15101334	79.23%
L-IAA	202771475	119277338	27473546	73.82%
S-ABA	172039514	101199714	29702116	77.61%
S-GA	150977144	99171261	18531704	79.55%
S-IAA	161257784	105924230	17738356	78.21%

Table S3| The expression data (FPKM-average) of *MADS-box* gene models in different tissues of AP85.

Gene Model	Leaf	Root	Stem1	Stem2	Stem3
SsSOC1-6a	32.14422	12.61744	36.79667	61.49424	46.26613
SsSOC1-6b	13.9211	14.28872	25.01661	46.06356	37.96133
SsSOC1-13	16.71323	4.91871	8.214317	0.617997	1.444801
SsAG/SHP-12	0	0	0	0	0.059466
SsAG/SHP-24	0	0	0	0.032047	0
SsAG/SHP-26	0	0	0	0	0
SsAG/SHP-10a	8.100087	0	6.610233	1.408773	1.537546
SsAG/SHP-10b	0.554179	0	0	0	0
SsSEP-22a	0	0	0	0	0
SsSEP-22b	0	0	0	0	0
SsSEP-1	1.426637	0	0	0.636807	0.046204
SsSEP-3	0	0	0	0	0
SsSEP-28	0	0	0	0	0
SsSEP-16	0	0	0	0	0
SsSEP-19	0	0	0	0	0
SsAP1/CAL/FUL-7/8	0	0	0	0	0
SsAP1/CAL/FUL-9	6.175635	3.298917	5.741807	20.57669	17.08832
SsAP1/CAL/FUL-25	0	0.078724	0.648554	0.967218	1.03928
SsSVP-29	6.108212	0.61663	7.6175	13.46481	11.94939
SsSVP-17	2.097826	0.815444	5.504778	9.84447	9.904498
SsSVP-4/5	24.26511	36.75418	30.85725	169.8139	100.3336
SsANRI-15	0.120207	0.559709	0	0	0
SsAGL12-like-21	2.807202	5.2651	3.518347	9.692531	6.006594
SsAGL12-like-18	0	0.304388	0	0	0
SsAGL12-like-23	1.287075	0.581703	0.514148	0.587066	0.678216
SsTT16-14	0	0	0	0	0
SsTT16-30	2.633524	2.91618	13.41589	5.524492	4.322533
SsTT16-20	0.205668	0.111122	0.047546	0.088118	0.142294
SsAP3/PI-31	0	0.3077	0.594326	0.0649	0.07437
SsAP3/PI-11	0	0.034718	0.315368	0.139333	0
SsAP3/PI-27	0.563741	0.46655	0.4725	0.209	0
SsAP3/PI-32	0.075215	0.0805	0.5581	0	0
SsMIKC-35*	0	0.075986	0	0	0.024832
SsMIKC-34*	0	0.102518	0	0	0
SsMα-49	0.87683	8.118726	0.986707	0.316954	1.736493
SsMα-56	0.640488	0.305445	1.786331	0.26619	0.275576
SsMα-53	0.554179	1.118648	0	0	0.425405
SsMα-40	0	0	0	0	0
SsMα-42	0	0	0	0	0
SsMα-41a	0	0	0	0	0
SsMα-51	0	0	0	0	0
SsMα-60	0	0	0	0	0
SsMα-62	0.522228	0.346058	0.225936	0.387953	0.525273
SsMα-63	0.158137	0	0.566178	0	0
SsMα-61	17.26523	2.963417	3.070172	0.598291	2.497617

SsMα-59	0.080133	0.1615	0.0999	0.34714	0
SsMα-48a	0	0	0	0	0
SsMα-48b	0	0	0	0	0
SsMα-36	0	0	0	0	0
SsMα-37	0	0	0	0	0
SsMα-38	0	0	0	0	0
SsMα-45	0	0	0	0	0
SsMα-58	0	0	0	0	0
SsMα-44/47	1.711792	0.444049	1.434042	1.093795	1.431495
SsMα-57	0.819045	0.194176	0.787096	1.253387	0
SsMβ-65	0	0	0	0	0
SsMβ-64a	0	0.068119	0	0	0
SsMβ-64b	0	0.019028	0	0	0
SsMβ-64c	0.056241	0.045385	0.038112	0.020547	0.017486
SsMγ-like-a	0	0	0	0	0
SsMγ-like-b	0	0.080489	0	0	0
SsMγ-like-c	0	0	0	0	0
SsMγ-like-d	0	0	0	0	0

Table S4| The expression data (FPKM-average) of MADS-box gene models in leaf and stem after ethylene treatment in SES-208.

Gene	L_ET_24h	L_ET_48h	L_ET_96h	S_ET_24h	S_ET_48h	S_ET_96h
SsSVP-4/5	49.5666667	52.2966667	45.67333333	74.69667	71.47333	88.11
SsSOC1_6a	25.76	41.34	24.42	82.72	58.77	35.41
SsSOC1-6b	65.395	72.65	70.945	72.845	100.7875	71.455
SsSVP-17	30.78	33.975	36.9375	55.6325	56.3675	64.1
SsSVP-29	19.6475	16.1425	21.7225	20.4025	30.88	27.9
SsAP1/CAL/FUL-9	26.315	25.025	22.165	14.435	15.255	21.5
SsTT16-30	4.115	4.235	7.295	9.605	9.27	16.555
SsAGL12-like21	2.1975	1.235	1.28	1.4175	1.94	1.9975
SsMα-61	1.02	2.87	4.8	0	0	0.84
SsSOC1-13	3.08	10.095	2.035	4.67	5.39	8.285
SsAG/SHP-10a	10.055	11.405	16.185	4.52	5.21	8.405
SsMα-49	0	0	0	1.1	0.9	0
SsAP1/CAL/FUL-25	0.235	0.22	1.76	3.975	0.76	11.815
SsMα-56	0	0	0	0.145	0	0.1825
SsAGL12-like-23	2.375	2.76	1.27	0.655	0.53	1.015
SsMα-44/47	1.32	0.995	1	0.355	0.29	2.405
SsSEP-1	1.97333333	2.87333333	1.88	0.623333	1.72	2.303333
SsMα-57	90.04	115.05	174.365	36.65	50.84	92.48
SsMα-53	0	0	0	0	0	0
SsAP3/PI-27	5.845	4.19	9.57	0.74	2.05	3.11
SsMα-62	2.53	2.51	2.176666667	1.606667	1.89	2.423333
SsAP3/PI-31	0.1875	0.085	0	0.1525	0.25	0.55
SsAP3/PI-11	0	0	0	0	0	0.273333
SsAP3/PI-32	0	0	0	0.32	0	0
SsMα-63	0	0	0	1.045	1.165	0.22
SsAG/SHP-10b	15.305	23.075	33.04	13.505	12.945	16.05
SsANR1-15	0	0	0	0	0.36	0
SsAGL12-like-18	0	0	0	0	0	0.07
SsMα-59	0	0	0	0.295	0.475	0.185
SsTT16-20	0.432	0.664	0.932	0.334	0.564	0.486
SsAG/SHP-12	0	0	0	0	0	0.14
SsAG/SHP-24	0	0	0	0	0	0
SsMβ-64b	0	0	0	0.24	0	0
SsMα-38	0	0	0	0	0	0
SsMα-45	0	0	0	0	0	0
SsMα-58	0	0	0	0	0	0
SsMβ-65	0	0	0	0	0.053333	0
SsMγ-64/65-like-a	0	0	0	0.063333	0	0
SsMγ-64/65-like-c	0	0	0	0	0	0
SsMγ-64/65-like-d	0	0	0	0	0	0
SsAG/SHP-26	0	0	0	0.125	0.3	0.6325
SsSEP-22a	0	0	0	0.2675	0.1075	0
SsSEP-22b	0	0	0	0	0	0
SsSEP-3	0	0	0	0	0	0
SsSEP-28	0	0	0	0	0	0

SsSEP-16	0	0	0	0	0	0
SsSEP-19	0	0	0	0	0	0.083333
SsAP1/CAL/FUL-7/8	0	0	0	0	0	0
SsTT16-14	0	0	0	0	0	0
SsMα-40	0	0	0	0	0	0
SsMα-42	0	0	0	0	0	0
SsMα-41a	0	0	0	0	0	0
SsMα-51	0	0	0	0	0	0
SsMα-60	0	0	0	0	0	0
SsMα-48a	0	0.08333333	0	0.113333	0.093333	0
SsMα-48b	0.42	0.13	0	0	0.03	0
SsMα-36	0	0	0	0	0	0
SsMα-37	0	0	0	0	0	0
SsMβ-64c	0	0	0	0	0	0
SsMIKC-34*	0	0	0	0	0	0
SsMIKC-35*	0	0	0	0	0	0
SsMβ-64a	0	0	0	0	0	0
SsMγ-64/65-like-b	0	0	0	0	0	0

Table S5| The expression data (FPKM-average) of *MADS-box* gene models in leaf and stem after 48 hr of hormones treatment in SES-208.

Gene	L-ABA	L-GA	L-IAA	S-ABA	S-GA	S-IAA
SsSOC1_6a	37.49	43.28	85.33	50.53	45.4	28.93
SsSOC1-6b	69.7125	79.6325	69.475	61.055	51.8225	86.7675
SsSOC1-13	4.185	2.025	4.1	5.135	2.955	5.09
SsAG/SHP-12	0	0	0	0	0	0.2
SsAG/SHP-24	0	0	0	0	0	0
SsAG/SHP-26	0	0.0675	0	0.675	0	0
SsAG/SHP-10a	3.48	6.355	6.525	3.775	5.305	1.92
SsAG/SHP-10b	12.13	16.025	4.395	5.31	14.09	9.72
SsSEP-22a	0	0.0725	0.2225	0	0	0
SsSEP-22b	0	0	0	0	0	0
SsSEP-1	2.233333	1.09	0.806667	1.983333	1.87	1.33
SsSEP-3	0	0	0	0	0	0
SsSEP-28	0	0	0	0	0	0
SsSEP-16	0	0	0	0	0	0
SsSEP-19	0	0	0	0	0	0
SsAP1/CAL/FUL-7/8	0	0	0	0	0	0
SsAP1/CAL/FUL-9	17.135	11.52	17.555	9.925	13.315	14.49
SsAP1/CAL/FUL-25	0	0.17	0.255	5.71	3.005	4.945
SsSVP-29	12.46	12.35	22.04	22.37	16.52	19.6175
SsSVP-17	23.585	28.02	32.785	51.02	50.5325	42.1575
SsSVP-4/5	29.51667	42.64	46.88667	64.81333	71.08	85.65667
SsANR1-15	0.445	0	0	0	0	0.25
SsAGL12-like21	1.32	0.535	1.32	1.63	0.935	0.57
SsAGL12-like-18	0	0	0	0	0	0
SsAGL12-like-23	0.27	1.355	1.8	0.77	0.395	0.85
SsTT16-14	0	0	0	0	0	0
SsTT16-30	2.23	3.285	4.9	12.655	12.56	8.29
SsTT16-20	0.858	0.432	0.562	0.52	0.41	0.502
SsAP3/PI-31	0	0	0	0.24	0.28	0.185
SsAP3/PI-11	0.18	0	0	0	0	0
SsAP3/PI-27	11.96	5.045	0.935	1.48	3.73	2.245
SsAP3/PI-32	0	0	0	0.32	0.63	0
SsMIKC-35*	0	0	0	0	0	0
SsMIKC-34*	0	0	0	0.063333	0	0.056667
SsMα-53	0	0	0	0	0	0
SsMα-49	0	0.52	0.385	0	0	0.465
SsMα-56	0.145	0.385	0.555	0.11	0	0.07
SsMα-40	0	0	0	0	0	0
SsMα-42	0	0	0	0	0	0
SsMα-41a	0	0	0	0	0	0
SsMα-51	0	0	0	0	0	0
SsMα-60	0	0	0	0	0	0
SsMα-62	2.36	3.103333	3.67	1.256667	1.666667	2.376667
SsMα-63	0	0.19	0	0	0.45	0.32
SsMα-61	1.69	0.73	0	1.64	0.86	0.92

SsMα-59	0	0.475	0.24	0	0.56	0.135
SsMα-48a	0.133333	0	0	0	0.073333	0
SsMα-48b	0.033333	0.4	0.306667	0.02	0	0
SsMα-36	0	0	0	0	0	0
SsMα-37	0	0	0	0	0	0
SsMα-38	0	0	0	0	0	0
SsMα-45	0	0	0	0	0	0
SsMα-58	0	0	0	0	0	0
SsMα-44/47	0.875	1.7	0.86	1.36	1.12	2.395
SsMα-57	80.965	79.41	45.89	42.185	47.75	30.145
SsMβ-65	0	0	0	0	0	0
SsMβ-64a	0	0	0	0	0.056667	0
SsMβ-64b	0	0	0	0.25	0	0
SsMβ-64c	0	0	0	0	0	0.053333
SsMγ-64/65-like-a	0	0	0	0	0.018333	0
SsMγ-64/65-like-b	0	0	0	0	0	0
SsMγ-64/65-like-c	0	0	0	0	0	0
SsMγ-64/65-like-d	0	0	0	0	0	0

Table S6. The primer sequences used for qRT-PCR

Gene	Representative allele	Forward primers(5'-3')	Reverse primers(5'-3')
SsSOC1-6b	*Sspon.001A0001360*	ACGTGAAAAGTGCGAGAAGC	GCCGGGCAATCCTATGAACA
***SsSOC1*_6a**	*Sspon.001B0000912*	ATGAAGCGGATAGAGAATCCG	ATTCGAAGAGCTTGCCGC
SsSVP-29	*Sspon.008B0015440*	ATACGGCGGATAGAGAACGC	GCGGAGTAGAGCTGAGCAAA
SsSVP-4/5	*Sspon.001C0005280*	AGAAGACCCTTGAATCCGGC	CTGCCCTTCCTCGTACACAA
***SsSVP*-17**	*Sspon.004A0003600*	TCGACTTGAACTTAGAACATAGCA	ATCTGTTCCAAGAATTGTTGAT
**GAPDH**		CACGGCCACTGGAAGCA	TCCTCAGGGTTCCTGATGCC

**Table S7**	**Accession numbers and sequences of 182 MADS-box in sugarcane**
**Accession no.**	**Sequences**
>Sspon.004B0010461	MGRGKIVIRRIDNSTSRQVTFSKRRNGLLKKAKELSILCDAEVGLIIFSSTGRLYDFSSTKQL<br>MGEELSGLGVRDLQNLESRLEMSLRSIRMRKVILIIHFISTFWFSIHFNLICNNVKQDHILKSE<br>IEELHRKGSLIHEENMELGRRVHIMSQQKVELQRKASEPRGVADASSSTPYSFSIRAQYADV<br>PANFEQRQSQQKEGSCLMLGATRCNTQTGQQQNVEPLEK*
>Sspon.004C0005610	MGRGKIVIRRIDNSTSRQVTFSKRRNGLLKKAKELSILCDAEVGLIIFSSTGRLYDFSSTKQL<br>MGEELSGLGVRDLQNLESRLEMSLRSIRMRKVILIIHFISTFWFSIHFNLICNNVKQDHILKSE<br>IEELHRKGSLIHEENMELGRRVHIMSQQKVELQRKASEPRGVADASSSTPYSFSIRAQYADV<br>PANFEQRQSQQKEGEPPYSNVASPLAISTHFAPLPQEFYLVLKICNQQSNMKCALHEEKFKC<br>AGPGISMLLATPVVASNKKGGSNTQSSFGD*
>Sspon.004B0003520	MGEELSGLGVRDLQNLESRLEMSLRSIRMRKDHILKSEIEELHRKGSLIHEENMELGRRVHI<br>MSQQKVELQRKASEPRGVADASSSTPYSFSIRAQYADVPANFEQRQSQQKEGEPPYSNVAS<br>PLAISTHFAPLPQEFYLVLKICNQQSNMKCALHEEKFKCAGPGISMLLAKPVVASNKKGGS<br>NTQSSFGGLVLGDDGLKSSEETGAADIKTKRLFVTGLSFYTSEKTLRAAFEPFGELVEVKIIM<br>DRISKRSKGYAFIEYTTEEAGGAALKAMNGQIADKIEGLLQEPSTAKEINQAAIVLDHWPDA<br>HAPH
>Sspon.004D0006200	MAVGRGIQKPASISMDSQISIRWSTSLPLKRECTEAKASQKLTLWQREAASLRQQLHNLQES<br>HKQLMGEELSGLGVRDLQNLESRLEMSLRSIRMRKVILIIHFISTFWFSIHFNLICNNVKQDHI<br>LKSEIEELHRKGSLIHEENMELGRRVHIMSQQKVELQRKASEPRGVADASSSTPYSFSIRAQ<br>YADVPANFEQRQSQQKE
>Sspon.006A0007900	MDSNIQNFDHNSLDYMHFLLLWQREAGSLRQQLHNLQEHHRQLLGQQLSGLDVRDLQNL<br>ENKLETSLRNIRLKKDQLIFDQIQELNRKGSLMHQENIELYNKVNLLHQENIELRRKVYGQG<br>VNEHPTSTTVRHSILNTENEDVSINLELSVQRDKSETPSI
>Sspon.006D0008030	MGRGKIEIKRIDNATSRQVTFSKRRSGLFKKAKELAILCDAEVGLVVFSSTGRLYDFASTRQ<br>LLGQQLSGLDVRDLQNLENKLETSLRNIRLKKDQLIFDQIQELNRKGSLMHQENIELYNKV<br>NLLHQENIELRRKVYGQGVNEHPTSTTVRHSILNTENEDVSINLELSVQRDKSETPSI
>Sspon.004A0012210	MGRGKIVIRRIDNSTSRQVTFSKRRNGIFKKAKELAILCDAEVGLMIFSSTGRLYEYSSTSMK<br>SVIDRYGKAKEEQQVVANPNSELKNFHSPYYDFMESACNIVAQQYPLQYLNILLVDPKFW<br>QREAASLRQQLHNLQENYRQLMGEDLSGLNVKELQSLENQLETSLRGVRAKKDHLLIDEIH<br>ELNRKASSFHQENRDLYNKINLIRQENAELHKKIYETEGSSGVNRESPTPFNFPVVETRDVP<br>VELGLSTLPQQNNIEPSTAPKL
>Sspon.004D0013821	MGEDLSGLNVKELQSLENQLETSLRGVRAKKDHLLIDEIHELNRKASSFHQENRDLYNKIN<br>LIRQENAELHKKIYETEGSSGVNRESPTPFNFPVVETRDVPVELGLSTLPQQNNIEPSTAPKL
>Sspon.001A0001360	MVRGKTQMKRIENPTSRQVTFSKRRNGLLKKAFELSVLCDAEVALVVFSPRGKLFEYASG<br>RASDLYSVQKTIERYRTYTKDNVSNKTVQQDIEQVKADAEGLAKKLEALDAYKRKLLGER<br>LEECSIEELHSLEVKLEKSLHCIRGRKTMLLEEQVNKLKEKEMNLRKSNEDLREKCEKQPP<br>MLMAPPPPPAPTVITVEDDHPEPKDDGVDVETELFIGLPGRDYRSNKDRAAVAIRSG*
>Sspon.001C0001370	MTYKYIDIVQKTIERYRTYTKDNVSNKTVQQDIEQVKADAEGLAKKLEALDAYKRKLLGE<br>RLEECSIEELHSLEVKLEKSLHCIRGRKTMLLEEQVNKLKEKEMNLRKSNEDLREKCKKQP<br>PMLMAPPLPPAPAVITVEDDHPEPKDDGVDVETELFIGLPGRDYRSSKDKAAVAIRSG*
>Sspon.001B0000911	CILNSDIILVTLFFSLFSVQKTIERYRTYTKDNVSNKTVQQDIEQVKADAEGLAKKLEALDA<br>YKRKLLGERLEECSIEELHSLEVKLEKSLHCIRGRKTMLLEEQVNKLKEKEMNLRKSNEDL<br>REKVMAGKHFTAMGRRCMRHANNPTTGFDYTAYLDLP*
>Sspon.001D0001730	MEELLQGNWSSPIMAAQVTLFFSLFSVQKTIERYRTYTKDNVSNKTVQQDIEQVKADAEGL<br>AKKLEALDAYKRKLLGERLEECSIEELHSLEVKLEKSLHCIRGRKTMLLEEQVNKLKEKEM<br>NLRKSNEDLREKCKKQPPMLVSPPPPPAPAVITVEDDHPEPKDDGVDVETELFIGLPGRDYR<br>SSKDKAAVAIRSG*
>Sspon.003B0033681	MLNMMTDLSCGPSEVTEQPAAPTGSGDKQGRGKIEIKRIENTTNRQVTFCKRRNGLLKKAY<br>ELSVLCDAEVALIVFSSRGRLYEYANNSVKSTIERYKKANSDTSNSGTVAEVSAQHYQQES<br>AKLRQTISSLQNANSRTIVGDSIHTMSLRDLKQLEGRLEKGISNIRARKNELLYAEVDYMQK<br>REMDLQTDNMYLRSKIAENNETGQPAMNMMGVPSTSEYDHMVPFDSRNFLQVNIMQQPQ<br>HYSHQLQPTTLQLG*
>Sspon.003D0025901	MFINFSPALVSAREGEGDKENHSPVPEPPKRGEVVRCSLPWPRPPRPMLSQTQTSASSAEEE<br>RGRSSPASAPPARPPVPRPSTHMVDSTHHTIRHAPVRPRGWCCSFAGVPESPEHRRALPASS<br>AIGTAVPKLLPPPPPPPPQVPAHAVVEAGGAHRPAKDPLAGPRVPDRLRGVPDAARSRRTV<br>DSAAFANGAPPRRCLGSRLRLRHRLLAIPHYQQESAKLRQTISSLQNANSRTIVGDSIHTMS<br>LRDLKQLEGRLEKGISNIRARKNELLYAEVDYMQKREMDLQTDNMYLRSKIAENNETGQP<br>AMNMMGVPSTSEYDHMVPFDSRNFLQVNIMQQPQHYSHQLQPTTLQLG*
>Sspon.007B0014670	MSPGGSASVAAGFAAERNNGKGKGKTEIKRIENTTNRQVTFCKRRNGLLKKAYELSVLCD<br>AEVALIVFSSRGRLYEYSNNSVKATIERYKKANSDNSSAAGTIAEVTIQHYKQESARLRQQI<br>TNLQNSNRALIGDSITTMSHKDLKHLEIRLDKGLGKIRARKNEVLCSELEYMQRREMELQN<br>DNLYLRSRVDENERAQQTVNMMGAPSTSEYQQQGFIPYDPIRSFLQFDIMQQPQFYSQQED<br>RKDFNLGGR*
>Sspon.007D0013010	MSPGGSASVAAGFAAERNNGKGKGKTEIKRIENTTNRQVTFCKRRNGLLKKAYELSVLCD<br>AEVALIVFSSRGRLYEYSNNSVKATIERYKKANSDNSSAAGTIAEVTIQHYKQESARLRQQI<br>TNLQNSNRALIGDSITTMSHKDLKHLEIRLDKGLGKIRARKNEVLCSELEYMQRREMELQN<br>DNLYLRSRVDENERAQQTVNMMGAPSTSEYQQQGFIPYDPIRSFLQFDIMQQPQFYSQQED<br>RKDFNLGRFYLPHLFE
>Sspon.007A0014650	MSHKDLKHLETRLDKGLGKIRARKEMELQNDNLYLRSRVDENERAQQTVNTMGAPSTSE<br>YQQQGFIPYDPIRSFLQFDIMQQPQFYSQQEDRKDFNLVKNKYETVKYLVQTIHNRTTEGD<br>WGQTYESVICSLQAILNHAELEIGGKPEKQRVCPSYWQCGGVQVR
>Sspon.007C0013540	MSPGGSASVAAGFAAERNNGKGKGKTEIKRIENTTNRQVTFCKRRNGLLKKASSNSFRSLS<br>LGSPDVSACSNSVTYLIILCVHDCSVKATIERYKKANSDNSSAAGTIAEVTIQHYKQESARL<br>RQQITNLQNSNRALIGDSITTMSHKDLKHLEIRLDKGLGKIRARKKYFVYAINEHPTLQNEV<br>LCSELEYMQRREMELQNDNLYLRSRVDENERAQQTVNMMGAPSTSEYQQQGFIPYDPIRS<br>FLQFDIMQQPQFYSQQEDRKDFNLGRFYLPHLFE
>Sspon.003D0004710	MGRGKIEIKRIENTTSRQVTFCKRRNGLLKKAYELSILCDAEIALVIFSCRGRLYEYSSNSVR<br>STIERYKKASASTSGTAPVIDINSLQFFQQEVGKLRQQIQTLQNSNRHLMGESIGNMTAKEL<br>KGLETRLERGIGRIRSKKNELLLAEIEYMQKREADLHNENMFLRAKVAEVERALQQEAAED<br>QTMTMVPAAVRGATTELKALPASFDPRGYYQYQQPQHVQASVLGAASAASSSQYSEQPQ<br>GQGQQEYHHQTALHLGYHVKIDDSAAGKGFL*
>Sspon.003C0007950	MGRGKIEIKRIENTTSRQVTFCKRRNGLLKKAYELSILCDAEIALVIFSSRGRLYEYSSNSYS
>Sspon.007B0028090	MGRGRIEIKRIENNTSRQVTFCKRRNGLLKKAYELSVLCDAEVALIVFSSRGRLYEYANNSV<br>KATIERYKKAHTVGSSSGPPLLEHNAQQFYQQESAKLRNQIQMLQNTNRHLVGDSVGNLS<br>LKELKQLESRLEKGISKIRARKNELLAAEINYMAKRETELQNDHMNLRTKIEEGEQQLQQV<br>TVARSVAAAAATNVELNPFLEMDTKCFFPGGPFATLDMKCFFPGSFQMLDAAAAQQRQM<br>LATELNLGYQLAPPGSDAANNNPH
>Sspon.007A0027950	MGRGRIEIKRIENNTSRQVTFCKRRNGLLKKAYELSVLCDAEVALIVFSSRGRLYEYANNSV<br>KATIERYKKAHTVGSSSGPPLLEHNAQQFYQQESAKLRNQIQMLQNTNRHLVGDSVGNLS<br>LKELKQLESRLEKGISKIRARKNELLAAEINYMAKRETELQNDHMNLRTKIEEGEQQLQQV<br>TVARSVAAAAATNVELNPFLEMDTKCFFPGGPFATLDMKCFFPGSFQMLEAAAAQQRQM<br>LATELNLGYQLAPPGSDAANNNPHQF*
>Sspon.007B0028081	MGRGRIEIKRIENNTSRQVTFCKRRNGLLKKAYELSVLCDAEVALIVFSSRGRLYEYANNR<br>HLVGDSVGNLSLKELKQLESRLEKGISKVRARKNELLAAEINYMAKRETELQNDHMNLRT<br>K
>Sspon.007D0021450	MGRGRIEIKRIENNTSRQVTFCKRRNGLLKKAYELSVLCDAEVALIVFSSRGRLYEYANNSV<br>KATIERYKKAHTVGSSSGPPLLEHNAQQFYQQESAKLRNQIQMLQNTNRHLVGDSVGNLS<br>LKELKQLESRLEKGISKVRARKNELLAAEINYMAKRETELQNDHMNLRTKIEEGEQQLQQV<br>TVARSVAAAAATNVELNPFLEMDTKCFFPGGPFATLDMKCFFPGSFQMLDAAAAQQRQM<br>LATELNLGYQLAPPGSDAANNNPH
>Sspon.007B0028270	MGRGRIEIKRIENNTSRQQVTFCMRRNGLLKKAYDELCAVLCYAVVALIVVFSSRGRLYER<br>VRQQQQVTFCMRRNGLLKKAYDELCAVICYAVVALIVVFSSRGCLYERVRQQQVCGRLSC<br>MHIGSLVNA
>Sspon.007D0021760	MGRGRIEFKRIENNTSRQQVTFCMRRNRLLKKAYDELCAVLCYAMAALIIVFSSRGRLYER<br>VRQQQDYEYTF*
>Sspon.002A0033930	MGRGKVQLKRIENKINRQVTFSKRRNGLLKKAHEISVLCDAEVAVIVFSPKGKLYEYASDS<br>RMDKILERYERYSYAEKALISAESESEGNWCHEYRKLKAKIETIQKCHKHLMGEDLESLNP<br>KELQQLEQQLESSLKHIRSRKLAERQKAAASRQQQQVQWDQRTQTQAQTSSSSSSFMMRQ<br>DQQGLPPPQNICFPPLTIGERGEEVAAAAAQQQPLPGQVQQQAQLRIS
>Sspon.002C0034720	MGRGKVQLKRIENKINRQVTFSKRRNGLLKKAHEISVLCDAEVAVIVFSPKGKLYEYASDS<br>RMDKILERYERYSYAEKALISAESESESSKFEVKCVPPSVITINLYRTANVSHASKNNRLCPL<br>APWMGNWCHEYRKLKAKIETIQKCHKHLMGEDLESLNPKELQQLEQQLESSLKHIRSRKS<br>HLMAESISELQKKLAERQKAAASRQQQQVQWDQRTQTQAQTSSSSSSFMMRQDQQGLPPP<br>QNIWYTSMLTSTSPCLNWSTPSHAMLVLYACISFPPLTIGERGEEVAAAAAQQQPPPEQVQ<br>QQAQLRISGLPPWMLSHLNA*
>Sspon.002C0034710	MGRGKVQLKRIENKINRQVTFSKRRNGLLKKAHEISVLCDAEVAVIVFSPKGKLYEYASDS<br>RMDKILERYERYSYAEKALISAESESEASNGEDLESLNPKELQQLEQQLESSLKHIRSRKSHL<br>MAESISELQKKVTKLKNFPGVTHR
>Sspon.002A0005163	MGRGPVQLRRIENKINRQVTFSKRRNGLLKKAHEISVLCDAEVALIVFSTKGKLYEYSSHCS<br>MEGILERYQRYSFEERAVLDPSIEDQADWGDEYVRLKSKLDALQKSQRHLLGERLDSLTIK<br>ELQQLEQQLDSSLKHIRSRKNQLMFDSISELQKKLMEAEKEKNNALMNAHLREQQNGAST<br>SSPSLSPPMVPDSMPTLNIGPCQPRGAGESEPEPSPAPVQANSGNLPPWMLSSVSNR*
>Sspon.002B0004041	MGRGPVQLRRIENKINRQVTFSKRRNGLLKKAHEISVLCDAEVALIVFSTKGKLYEYSSHSS<br>MEGILERYQRYSFEERAVLDPSIEDQADWGDEYVRLKSKLDALQKSQRHLLGEQLDSLTIK<br>ELQQLEQQLDSSLKHIRSRKNQLMFDSISELQKKVSYTLDSLVPRILHVLHESLVKIIVTSTPL<br>EFGE*

>Sspon.005A0006090	MGRGRVELKRIENKINRQVTFSKRRNGLLKKAYELSVLCDAEVALIVFSSRGKLYEFGSAG<br>VNKTLEKYHNCCYNAQNSNTGFDGEPQSWYREMSRLKDKLESLQRCQRHMLGEDLGPLSI<br>KELQQLEKQLDYSLSQARHRKTQMMMEQMDELRRKERQLGELNKKLKNKVSK*
>Sspon.005D0008462	MAARGGDRPARAKAVTPQAEADQIPLLPAEESCRYIPLYWGAGDRRGARSRWGGGRVEL<br>KRIENKINSQVTFSKRRNGLLKKAYELSVLCDAEVALIVFSSRGKLYEFGSAGVNKTLEKYH<br>NCCYNAQNSNTGFDGEPQSWYREMSRLKDKLESLQRCQRHMLGEDLGPLSIKELQQLEKQ<br>LDYSLSQARHRKTQMMMEQMDELRRKERQLGELNKKLKNKLEAEGCSNYRGVQTSWAT<br>DVAITSDSGALSTPNAEPPAAAVDCEPTLQIRFVAPEAAAMPRSNTEGGENSHFMLGWAL*
>Sspon.005B0004502	MGRGRVELKRIENKINRQVTFSKRRNGLLKRAYELSVLCDAEVALIVFSSRGKLYEFGSAG<br>YACIWPGVYVSRLRCRYWSLAHAILADLR*
>Sspon.004B0006672	MGRGRVELKRIENKINRQVTFSKRRNGLLKKAYELSVLCDAEVALIIFSSRGKLYEFGSAGI<br>TKTLERYQHCCYNAQDSNGALSETQSWYQEMSKLRAKFEALQRTQRHLLGEDLGPLSVKE<br>LQQLEKQLECALSQARQRKTQLMMEQVEELRRKERHLGEMNRQLKHKLEAEGSSNYRTL<br>QHAAWPAPGGTIVEHDGATYHVHPPAHSVAIDCEPTLQIGYNTCSIVIQETQMQLLCTNRS<br>NKTDSVSGTLITSFCLLRQPIISQGAPPEERTTTSCWDGFFELFSQSIHQMGDVETNKRHSVL<br>AIQFSVEYDH*
>Sspon.004D0008890	MGRGRVELKRIENKINRQVTFSKRRNGLLKKAYELSVLCDAEVALIIFSSRGKLYEFGSAGI<br>TKTLERYQHCCYNAQDSNGALSETQSWYQEMSKLRAKFEALQRTQRHLLGEDLGPLSVKE<br>LQQLEKQLECALSQARQRKTQLMMEQVEELRRKERHLGEMNRQLKHKLEAEGSSNYRTL<br>QHAAWPAPGGTIVEHDGATYHVHPPAHSVAIDCEPTLQI
>Sspon.006A0003760	MGRGRVELKRIENKINRQVTFAKRRNGLLKKAYELSVLCDAEVALIIFSNRGKLYEFSSTQS<br>MPKTLEKYQKCSFAGPETALQNRENEQLKSSRNEYLKLKARVDNLQRTQRNLLGEDLESL<br>GIKELEHLEKQLDSSLKHIRSTRGHAPPPADSAALQGYDPLPAGSAALPAGAAGVQGADPL<br>SDGAAGHISRAAGATFQPHPALHPAAARPPAAWTAPAMTAIGGAATGTPSTQHMVDQLTE<br>LQKRLEESNQVIWQHAWEQSERHPEVQPQQLHGTNNFFHPLDAAGEPTLQIGYPSEALTSP<br>CMTTFLPPWLP*
>Sspon.006D0002440	MGRGRVELKRIENKINRQVTFAKRRNGLLKKAYELSVLCDAEVALIIFSNRGKLYEFSSTQS<br>MPKTLEKYQKCSFAGPETALQNRENEQLKSSRNEYLKLKARVDNLQRTQRNLLGEDLESL<br>GIKELEHLEKQLDSSLKHIRSTRTQHMVDQLTELQKREQMFCEANKCLRRRLEESNQVIWQ<br>HAWEQSERHPEVQPQQLHGTNNFFHPLDAAGEPTLQIGYPSEALTSPCMTTFLPPWLP*
>Sspon.006C0003411	MGRGRVELKRIENKINRQVTFAKRRNGLLKKAYELSVLCDAEQLKSSRNEYLKLKARVDN<br>LQRTQRNLLGEDLESLGIKELEHLEKQLDSSLKHIRSTRTQHMVDQLTELQKREQMFCEAN<br>KCLRRRLEESNQVIWQHAWEQSERHPEVQPQQLHGTNNFFHPLDAAGEPTLQI
>Sspon.006D0002420	MGRGRVELKRIENKINRQVTFAKRRNGLLKKAMPKTLEKYQKCSFAGPETALQNRENEQL<br>KSSRNEYLKLKARVDNLQRTQRNLLGEDLESLGIKELEHLEKQLDSSLKHIRSTRTQHMVD<br>QLTELQKRMQRET*
>Sspon.002A0013910	MGRGRVELKRIENKINRQVTFAKRRNGLLKKAYELSVLCDAEVALIIFSNRGKLYEFCSGQS<br>ITKTLERYEKSGYGGPDTAVQNKENELVQSSRNENLLGEDLGSLGIKELEQLEKQLDSSLRH<br>IRSTRTQHMLDQLTDLQRREQMLCEANKCLRRKLEETSNQVHGQVWENGANLLGYERHS<br>PPQQAPSHVGNGLFFHPLEAAAEPTLQIGFAPEHMNNFMPTWLP*
>Sspon.002B0011890	MGRGRVELKRIENKINRQVTFAKRRNGLLKKAYELSVLCDAEVALIIFSNRGKLYEFCSGQS<br>ITKTLERYEKSSYGGPDTAVQNKENETIDFGFIGNYRNLLGEDLGSLGIKELEQLEKQLDSSL<br>RHIRSTRTQHMLDQLTDLQRREQMLCEANKCLRRKLEETSNQVHGQVWENGANLLGYER<br>HSPPQQAPSHVGNGLFFHPLEAAAEPTLQIGFAPEHMNNFMPTWLP
>Sspon.001C0022260	MGRGKVVLQRIENKISRQVTFAKRRNGLLKKAYELSILCDAEVALVLFSHAGRLYQFSSSS<br>NLLKTLERYQRYIYASADAAVPSSDEMQNNYQEYVKLKARVEVLQHSQRNLLGEDLAPLG<br>PSELDQLESQVDKTLKQIRSRKTQVLLDELCDLKRKEQMLQDANRVLKRKLDEFKAEAAS<br>PPQLAWQDGGGMLSHDPPQPEHFFQALESNPSLQPT*
>Sspon.001C0034400	MGRGKVVLQRIENKISRQVTFAKRRNGLLKKAYELSILCDAEVALVLFSHAGRLYQFSSSS<br>NLLKTLERYQRYIYASADAAVPSSDEMQNNYQEYVKLKARVEVLQHSQRNLLGEDLAPLG<br>PSELDQLESQVDKTLKQIRSRKTQVLLDELCDLKRKEQMLQDANRVLKRK
>Sspon.001B0044900	MGRGKVVLQRIENKISRQVTFAKRRNGLLKKAYELSILCDAEVALVLFSHAGRLYQFSSSS<br>NLLKTLERYQRYIYASADAAVPSSDEMQNNYQEYVKLKARVEVLQHSQRNLLGEDLAPLG<br>PSELDQLESQVDKTLKQIRSRKTQVLLDELCDLKRKEQMLQDANRVLKRKLDEFKAEAAS<br>PPQLAWQDGGGMLSHDPPQPEHFFQALESNPSLQPTYHTMDMNQEPVPEPGGCYPPAWM<br>A
>Sspon.008B0018213	MGRGKVELKRIENKISRQVTFAKRRNGLLKKAYELSVLCDAEVALIIFSSRGRLFEFSTSSC<br>MYKTLERYRSCNFASEASAPLEAELNNYQEYLKLKTRVEFLQTTQRNLLGEDLGPLNVKEL<br>EQLENQIEISLKHIRSSKNQQMLDQLFDLKRKEQQLQDANKDLRRKIQETSEENVLRLSCQD<br>IACGGSSGHGDEANQERLQLALDPSLHI
>Sspon.008D0017856	MGRGKVELKRIENKISRQVTFAKRRNGLLKKAYELSVLCDAEVALIIFSSRGRLFEFSTSSC<br>MYKTLERYRSCNFASEASAPLEAELNNYQEYLKLKTRVEFLQTTQRNLLGEDLGPLNVKEL<br>EQLENQIEISLKHIRSSKNQQMLDQLFDLKRKEQQLQDANKDLRRKIQETSEENVLRLSCQD<br>IACGGSSGHGDEANQERLQLALDPSLHIGEKGKK*
>Sspon.008A0018143	MGRGKVELKRIENKISRQVTFAKRRNGLLKKAYELSVLCDAEVALIIFSSRGRLFEFSTSSC<br>MYKTLERYRSCNFASEASAPLEAELNNYQEYLKLKTRVEFLQTTQRNLLGEDLGPLNVKEL<br>EQLENQIEISLKHIRSSKNQQMLDQLFDLKRKEQQLQDANKDLRRKIQETSEENVLRLSCQD<br>IACGGSSGHGDEANQERLQLALDPSLHIGEKGKKNEST*
>Sspon.001B0007520	MGRGKVELKRIENKISRQVTFAKRRNGLLKKAYELSLLCDAEVALIIFSGRGRLFEFSSSSCF<br>AYNSSSKLSMYKTLERYRSSNYSSQEVKTPLDSEINYQDYLKLRTRVEFLQTTQRNILGEDL<br>GPLSLKELEQLENQIETSLKHIRSREEQELQDLNKDLRKKGLLQHPENYPSLQIGYHQQAYM<br>DQLNNEDMADPNEHGRSGWI*
>Sspon.001C0005651	VTFAKRRNGLLKKAYELSLLCDAEVALIIFSGRGRLFEFSSSNILGEDLGPLSLKELEQLENQI<br>ETSLKHIRSRENQMLLDQLFDLKSKEQELQDLNKDLRKK
>Sspon.001D0006180	MGRGKVELKRIENKISRQVTFAKRRNGLLKKAYELSLLCDAEVALIIFSGRGRLFEFSSSSC<br>MYKTLERYRSSNYSSQEVKAPLDNEVSINYQDYLKLRTRVEFLQTTQRNILGEDLGPLSLKE<br>LEQLENQIETSLKHIRSRENQMLLDQLFDLKSKVTTMSSGCLLPNFISVPCIRTKPFIAELPIM<br>LQEQELQDLNKDLRKKCQLQETSPENVLHVSSWGEGGHSGASGNVLDPYQGLLQHPENYP<br>SLQI
>Sspon.001D0006230	MGRGKVELKRIENKISRQVTFAKRRNGLLKKAYELSLLCDAEVALIIFSGRGRLFEFSSSSCF<br>AYNSSSKLRNILGEDLGPLSLKELEQLENQIETSLKHIRSRENQMLLDQLFDLKSKEQELQDL<br>NKDLRKKLQETSPENVVHVSSWGEGGHSGASGNVLDPYQGLLQHPENYPSLQIG*
>Sspon.004D0028033	MQIHPSPPDHLMVMATAAAAMDVDAPAAPDGNAANKRGRRGRREMRRIEDTTSRQVTFS<br>KRRSGLLKKAYELSVLCDAEVALIVFSPRGRLYQFASAADLQNTIDRYLKHTEGTPSNGKV<br>ETGVEDKKLMDQILDLREKEQKLLMENAMLRDQCKTLPLLELNDKDHDHHMDAASGGQE<br>EEEAAAAAAKEGRMEDVVTELAIGIIGSRRLTESPALRLHQQPAGSYRRSSA*
>Sspon.004D0028070	MQIHPPPVHLMVMTTAAAAMDVDAPAAPDGNAANKAARQGRRGRREMRRIEDTTSRQV<br>TFSKRRSGLLKKAYELSVLCDAEVALIVFSPRGRLYQFASAPEYALVMLNALASAFKNTID<br>RYLKHTEGTPANGEVETGVEDKKLMDQILDLREKEQKLLMENSMLRDQCKALPLLELNDK<br>DHDHHMDAAGGGQEEEEAAAVKEGRMEDVETELTIGIFGSREAY
>Sspon.008A0005210	MSSQHQIVYQQAQQFHQQLQQQQQQQLQQFWAERMAEIEATTDFKNHNLPLARIKKIMK<br>ADEDVRMISAEAPVVFAKACEIFILELTLRSWMHTEENKRRTLQKNDIAAAITRTDIYDFLV<br>DIVPRDEMKEDGIGLPRAGLPPMGAPADAYPYYYMQQQQLLKSSSLCMKVAENVAQKLSP<br>DSDLMGRGKVEMKRIENKVSRQVTFSKRRKGLLKKAEELAVLCDVDVGVIVFSERGKLFD<br>YSSPARRSPTRNSSIRRHTALTIRYADPQQMAAEISKLQHECEQLEASLKTYTGEDLSSLTSV<br>VELGELEQQLESAVGK
>Sspon.008B0005242	MGVAAAGSQVYPASAYPPAATVAAPAVASAGLQSVQPFPANPAHMSSQHQIVYQQAQQF<br>HQQLQQQQQQQLQQFWAERMAEIEATTDFKNHNLPLARIKKIMKADEDVRMISAEAPVVF<br>AKACEIFILELTLRSWMHTEENKRRTLQKNDIAAAITRTDIYDFLVDIVPRDEMKEDGIGLPR<br>AGLPPMGAPADAYPYYYMQQQQLLKSSSLCMKVAENVAQKLSPDSDLMGRGKVEMKRIE<br>NKVSRQVTFSKRRKGLLKKAEELAVLCDVDVGVIVFSERGKLFDYSSPARRSPTRNSSIRRH<br>TALTIRYADPQQMAAEISKLQHECEQLEASLKTYTGEDLSSLTSVVELGELEQQLESAVGK<br>DELFINQTDELQLKINEHGRHDGAAGAAGVEAEETTTMAEPPLPQSPSFAYLLAVEEKSAA<br>STMLRLWPQPDDDADADADADGGTVVTNRVRVRPKLA*
>Sspon.001B0000912	MVRGKTQMKRIENPTSRQVTFSKRRNGLLKKAFELSVLCDAEVALVVFSPRGKLFEYASG<br>R*
>Sspon.001D0001740	MVRGKTQMKRIENPTSRQVTFSKRRNGLLKKAFELSVLCDAEVALVVFSPRGKLFEYASG<br>R*
>Sspon.003C0033440	MARRRGRVELRRIEDRVSRQVRFSKRRAGLFKKAFELSLLCDAEVALLVFSPAGKLYEYAS<br>AR*
>Sspon.003B0003800	MSRRGRVELRRIEDKASRQVRFSKRRAGLFKKAFELALLCDAEVALLVFSPGGKLYEYSST<br>RFSTPAPLSLLSSYNRLPVDRSETEAGEAPPYNLAVEQGRSSIRSGPILESRRCSTASRARGGP<br>PAAVLVHAMCSRQSPIPGGCACATLHDSVWDLCPACRCRSRRHLRFFLPRSATCSMSLFWT<br>LAALGRAVQLAGLLGVL
>Sspon.006D0002312	MVMLLAAAAIRPQLPSPSLSPHREFVRILLWLRPAFHPLPHSSEVTRQSSRASYGYDPAFHP<br>LPIELFVGGALVLALAVSFASLGKMEGEPPQKQERRKRGRVELRRIEDRTSRQVRFSKRRR<br>GLFKKAHELSVLCDAQVALVVFSPAGRLHEFASADSSIEKTFCRYWDLANTTIDLNIEARD<br>DSRVNYNIQ
>Sspon.006D0002320	MEGEPPQKQERRKRGRVELRRIEDRTSRQVRFSKRRRGLFKKAHELSVLCDAQVALVVFSP<br>AGRLHEFASADSSIEKTFCRYWDLANTTIDLNIEARDDSRVNYNIQVKHNK*
>Sspon.008D0014300	MGRVKLQIKRIENTTNRQVTFSKRRNGLIKKAYELSVLCDIDIALIMFSPSNRLNHFSGRRRI<br>EDVIMRYINLPEHDRGGVVRNREYLIKMLTQLKCEGDIAEQLAPNKGPVNSNVEELQQEIR<br>TYQHQLQVLEERLRMFEPDPVALASMNEKYLLCNHMGPFEPSPSDMHHVFGLPPPPQQQQ<br>QQEEEQQGDLGVNAFGGDVSSWFADGLPTSSIFAGPDPILSFRDQVIFDSMRRDPVVAGVD<br>PGIASMCHVDQQVPSDDWQQAYTSAELLSALIPSTPFPLDDQVTWHGSGPSSSTSTTAALLS<br>PELARYGVIQMQDAMAPVLTSPMVPPHVHEQVEAPPGSCSNVPTDGDCATATAAAQEHGL<br>PARSTSASCICIS
>Sspon.008B0015130	MGRVKLQIRRIENNTNRQVTFSKRRNGLIKKAYELSVLCDIDIALIMFSPSNRLNHFSGRRRI<br>EDVIMRYINLPEHDRGGVVRNREYLIKMLTQLKCEGDIAEQLAPNKGPVNSNVEELQQEIR<br>TYQHQLQVLEERLRMFEPDPVALASMNEKYLLCNHMGPFEPSPSDMHHVRVRHAAPPPPQ

	QQQEEEQQGDLGVNAFGGDVSSWFADGLPTSSIFAGPDPILSFRDQVIFDSMRRDPVVAGVDPGIASMCHVDQQVPSDDWQQAYTSTELLSALIPSTPFPLDDQVTWHGSGPSSSTSTTAALLSPELARYGRHGAGAHVSDGAAAVHEQVEAPPGSCSNVPTDGDCATATAAAQEHGLPGAVNIG*
>Sspon.008D0014290	MGRVKLQIRRIENNTNRQVTFSKRRNGLIKKAYELSVLCDIDIALIMFSPSNRLNHFSGRRRIEDVIMRYINLPEHDRGGVVRNREYLIKMLTQLKCEGDIAEQLAPNKGPVNSNVEELQQEIRTYQHQLQVLEERLRMFEPDPVALASMNEVETNDRISRSRYINRRACVHSLQKYLLCNHMGPFEPSPSDMHHVFGLPPPQQQQQQQEEEQQGDLGVNAFGGDVSSWFADGLPTSSIFAGPDPILSFRDQVIFDSMRRDPVVAGVDPGIASMCHVDQQVPSDDWQQAYTSTELLSALIPSTPFPLDDQVTWHGSGPSSSTSTTAALLSPELARYGVIQMQDAMAPVLTSPMVPPHVHEQVEAPPGSCSNVPTDGDCATATAAAQEHGLPRAVNIG*
>Sspon.008A0014421	MGRVKLQIRRIENNTNRQVTFSKRRNGLIKKAYELSVLCDIDIALIMFSPSNRLNHFSGRRRSVFGMPPPPQQQQEEEQQGDLGVNAFGGDVSSWFADGLPTSSIFAGPDPILSFRDQVIFDSMRRDPVVAGVDPGIASMCHVDQQVPSDDWQQAYTSAELLSALIPSTPFPLDDQVTWHGSGPSSSTSTTAALLSPELARYGVIQMQDAMAPVLTSPMVPPHVHEQVEAPPGSCSNVPTDGDCATATAAAQEHGLPGA
>Sspon.008B0015123	MGRVKLQIRRIENNTNRQVTFSKRRNGLIKKAYELSVLCDIDIALIMFSPSNRLNHFSGRRRSVFGMPPPPPQQQQQQEEEQQGDLGINAFGGDVSSWFADGLPTSSIFAGPDPILSFRDQVIFDSMRRDPVVAGVDPGIASMCHVDQQVPSDDWQQAYTSTELLSALIPSTPFPLDDQVTWHGSGPSSSTSTTAALLSPELARYGVIQMQDAMAPVLTSPMVPPHVHEQVEAPPGSCSNVPTDGDCATATAAAQEHGLPGA
>Sspon.005C0009410	MLCLLYSFDQVAESENIKGSCPTWKSDNNGRHQEYELRRKLGNEDQWQPLALDPSWGDQWVACHGKSKLLAAWLPCSMRRIENPVHRQVTFCKRRMGLLKKAKELSVLCDADIGVIVISPHGKIYDLATNGSIIKQEVLALTHEIDLLQKGFRYMHAENGENDINHMNLDELQTLENKLEMWVNNIRSQKMQIISREIEMLRNKVGSVSVVLFLQQTQEAMLQAVNGVLQERQHSKHLLCLTSSNTPAPLLLTSSKNSKSPAPLYLTTSNTASRSVPPIHPCKHHNRGKMNTTVPSTTTVAAAAESLHSRVTSTRADASPKQSATPTAPPQLQPPGISRCMLKTESES*
>Sspon.005D0015580	MARGKVQMRRIENPVHRQVTFCKRRMGLLKKAKELSVLCDADIGVIVISPHGKIYDLATNGSVILSCLLMQTNMQGLIERYWRTYSEMCGESSNHNKTQIIKQEVLALTHEIDLLQKGFRYMHAENGENDINHMNLDELQTLENKLEMWVNNIRSQKMQIISREIEMLRNKEAMLQAVNGVLQEKVLQIC
>Sspon.005D0015600	MARGKVQMRRIENPVHRQVSFCKRRMGLLKKAKELSVLCDADIGVIVISPHGKIYDLATNGNMQGLIERYRRTYSEMRGESSNHNKTQIIKQEVLALTHEIDLLQKGFRYMHGENGENGINHMNLDELQTLENNLEMWVNNI
>Sspon.006D0016052	MARGKVQLRRIENPVHRQVTFCKRRAGLLKKARELSVLCDAHIGIIIFSAHGKLYDLATTGYVIVIVSSSSTMEELIERYKTASGEAADSSGDNRMDPKQETMVLQQEINLLQKGLRYIYGNRANEHMNVEELNALERYLEIWMYNIRSAKEGMLKAANEVLQEKIVEQSSLLDVGMVVADQQNGHFSTVPLIEEITNPLTILSGYSN
>Sspon.006A0017650	MARGKVQLRRIENPVHRQVTFCKRRAGLLKKARELSVLCDAHIGIIIFSAHGKLYDLATTGYVIVTVSTMEELIERYKTASGEAADSSGDNRMDPKQETMVLQQEINLLQKGLRYIYGNRANEHMNVEELNALERYLEIWMYNIRSAK
>Sspon.006B0017210	MARGKVQLRRIENPVHRQVTFCKRRAGLLKKARELSVLCDAHIGIIIFSAHGKLYDLATTGTMEELIERYKTASGEAADSSGDNRMDPKQETMVLQQEINLLQKGLRYIYGNRANEHMNVEELNALERYLEIWMYNIRSAKEGMLKAANEVLQEKSWYQCSATSDPSTAPVIAPNPVASRTPAVNHSHPPTNAADEQQQNQTDVTTDLENLAATHGRKGTPSVIPCGALENLSATPPRWAAPTRPPASANFLDSRRALSCGNRICGQRRGEARRGGRGRGADGIADLGPVDRVGGDELVDVGVGPLGEGLGAAVDLLEVVEEGEVARPVLLHALEVLTDPHRHRRFAFTWIRFDGEEVVWCCCPCCGWSGGRREEEDATQSDDKWAWMDRAGMVLLHGLSPT*
>Sspon.006C0016990	MARGKVQLRRIENPVHRQVTFCKRRAGLLKKARELSVLCDAHIGIIIFSAHGKLYDLATTGTMEELIERYKTASGEAADSSGDNRMDPKQETTVLQQEINLLQKGLRYIYGNRANEHMNVEELNALERYLEIWMYNIRSAKEGMLKAANEVLQEK
>Sspon.003B0000024	MGRGRSEIKRIENPTQRQSSFYKRRDGLFKKARELSVLCDVDLLLLLFSTSGKLYHYLSPTVPSVKDLVERYEAATHTKVWTDIRQERRAELEKAEQMCELMEKELRYMTVDDGEQYTVPSLELLEHNLEAAVHKVRSEKDRKIGGEINYLENIIRGRHEERYGLCDKLAHSQASNNDGEGESAPPSSGLELKLGTIQSLCGLLNFDSDQLFQYT*
>Sspon.003C0008970	MGRGRSEIKRIENPTQRQSTFYKRRDGLFKKARELSVLCDVDLLLLLFSTSGKLYHYLSPTVPSVKDLVERYEAATHTKVWTDIRQERRAELEKAEQMCELMEKELRFMTVDDGEQYTVPSLELLEHNLEAAVHKVRSEKDRKIGGEINYLENIIRGRHEERYGLCDKLAHSQASNNDGEGGSAPPSSGLELKLGTIQSLCGLLNFDSDQLF
>Sspon.001C0005280	MAGKRERIAIRRIDNLAARQVTFSKRRRGLFKKAEELSILCDAEVGLVVFSATGKLFHFASTSMKQVIDRYDSHSKNLQKSEALSQLQSHIDDGTCSRLKEELAQTSLKLRQMRGEELQRLSVQQLQELEKTLESGLGSVLKTKRMELIEENSRLKEQVTRMARMETQLGVDSEIVYEEGQSSESVTNTSYPRPSTDTDDCSDTSLRLG*
>Sspon.001D0004841	MRLCSINIVAVGHWSFAKRYSYQPLDCSRSAPQIYSNTSAAAPPRVEEQERGTMAGKRERIAIRRIDNLAARQVTFSKRRRGLFKKAEELSILCDAEVGLVVFSATGKLFHFASTSMKQVIDRYDSHSKNLQKSEALSQLQSHIDDGTCSRLKEELAQTSLKLRQMRGEELQRLSVQQLQELEKTLESGLGSVLKTKRMELIEENSRLKEQVTRMARMETQLGVDSEIVYEEGQSSESVTNTSYPRPSTDTDDCSDTSLRLGNFDANVAYRAAEQ
>Sspon.001B0036011	MRLCSINRSGRHCCGAAAGGGARERHDGREEGADSDTEIDNLAARQVTFSKRRRGFQKAEELSILCDAEVGLVVFSATGKLFHFAST
>Sspon.008A0014682	MARERREIRRIENAAARQVTYSKRRRGLFKKAEELAVLCDADVALLVFSSTGKLSHMNDIIDKYSTHSKNLGKSHQQPPIDLNMRGEDLEGLSVEELHQMERKLEAGLHRVLSTKDQLFTQQISELHQKVTSIRRALSGDQLRLSHSHVPSDLGRTS*
>Sspon.008D0014681	MLANIMNDIIDKYSTHSKNLGKSHQQPPIDLNVEQNRYTSLNEQLAEATHGLRQMRGEDLEGLSVEELHQMERKLEAGLHRVLSTKDQLFTQQISELHQKVTSIRRALSGDQLRLSHSHVPSDLGRTS*
>Sspon.008C0014931	MARERREIRRIENAAARQVTYSKRRRGLFKKAEELAVLCDADVALLVFSSTGKLSHMNDIIDKYSTHSKNLGKSHQQPPIDLNMRGEDLEGLSVEELHQMERKLEAGLHRVLSTKDQLFTQQISELHQKVTSIRRALSGDQLRLSHSHVPSDLSRTS*
>Sspon.008B0015440	MARERREIRRIENAAARQVTYSKRRRGLFKKAEELAVLCDADVALLVFSATGKLSQFASSRHTLTSLPTFAQLYSAHPTPTSITSAVFRNLLFSPCMNDIIDKYSTHSKNLGKSHQQPPIDLNVEQNRHTSLNEQLAEATHGLRQMRGEDLEGLSVEELHQMERKLEAGLHRVLSTKDQLFTQQISELHQKMPQVRTAGTTVVAAGAENILTEDGQSSESVMTALHSGSSLDNDDGSDISLKL
>Sspon.004B0002060	MNEIIDKYNTHSKNLGKAEEPSLDLNLEHSKYANLNEQLVEASLRLRQMRGEELEGLSVEELQQLEKNLETGLHRVLQTKDQQFLEQISDLERKLSRSERSYLNLRI
>Sspon.006C0006131	MARERREIKRIESAAARQVTFSKRRRGLFKKAEELSVLCDADVALIVFSSTGKLSQFASSMNEIIDKYNTHSKNLGKAEEPSLDLNLEHSKYANLNEQLVEASLRLRQMRGEELEGLSVEELQQLEKNLETGLHRVLQTKDQQFLEQISDLERKVINNNCIVVFSCIG*
>Sspon.004A0003600	MARERREIKRIESAAARQVTFSKRRRGLFKKAEELSVLCDADVALIVFSSTGKLSQFASSMNEIIDKYNTHSKNLGKAEEPSLDLNLEHSKYANLNEQLVEASLRLRQMRGEELEGLSVEELQQLEKNLETGLHRVLQTKDQQFLEQISDLERKSTQLAEENMQLRNQVSQIPPSGKQAVADTENVIAEDGQSSESVMTALHSGSSQDNDDGSDVSLKLGKHSVVIKVWMT*
>Sspon.004C0004320	MARERREIKRIESAAARQVTFSKRRRGLFKKAEELSVLCDADVALIVFSSTGKLSQFASSMNEIIDKYNTHSKNLGKAEEPSLDLNLEHSKYANLNEQLVEASLRLRQMRGEELEGLSVEELQQLEKNLETGLHRVLQTKDQQFLEQISDLERKSTQLAEENMQLRNQVSQIPPSGKQAVADTENVIAEDGQSSESVMTALHSGSSQDNDDGSDVSLKLGKLQKRLRVDLGGSCTASADRHLCVLVLWTTPDYCNKLALEARSVNLICHPCGFMMRCSACTCHVTKRRKHSVVIKV*
>Sspon.004B0022640	MGRGKIEIKRIENSTNRQVTFSKRRGGLLKKANELAVLCDARVGVVIFSSTGKMFEYCSPACSLRELIEQYQHATNSHFQEINHDQQILLEMTRMKNEMDKLETGIRRYTGDDLSSLTLDDVSDLEQQLEYSVSKVRARKHQLLNQQLDNLRRKEQILEDQNTFLYRMINENQQAALTGEVKLGEMTAPLAMLPPPAAFAHSTYYGGESSSSGTALQLMSAAPQLQHADLGFRLQPTQPNLQDPAAACGGLHGHGLQL*
>Sspon.004B0022650	MGRGKIEIKRIENSTNRQVTFSKRRGGLLKKANELAVLCDARVGVVIFSSTGKMFEYCSPACSLRELIEQYQHATNSHFQEINHDQQILLEMTRMKNEMDKLETGIRRYTGDDLSSLTLDDVSDLEQQLEYSVSKVRARKHQLLNQQLDNLRRKEQILEDQNTFLYRMINENQQAALTGEVKLGEMTAPLAMLPPPAAFAHSTYYGGESSSSGTALQLMSAAPQLQHADLGFRLQPTQPNLQDPAAACGGLHGHGLQL
>Sspon.004C0022071	MGRGKIEIKRIENSTNRQVTFSKRRGGLLKKANELAVLCDARVGVVIFSSTGKMFEYCSPACSLRELIEQYQHATNSHFQEINHDQQILLEMTRMKNEMDKLETGIRRYTGDDLSSLTLDDVSDLEQQLEYSVSKVRARKHQLLNQQLDNLRRKEQILEDQNTFLYRMINENQQAALTGEVKLGEMTAPLAMLPPPAAFAHSTYYGGESSSSGTALQLMSAAPQLQHADLGFRLQPTQPNLQDPAAACGGLHGHGLQL
>Sspon.004A0021913	MGRGKIEIKRIENSTNRQVTFSKRRGGLLKKANELAVLCDARVGVVIFSSTGKMFEYCSPACRLVVGCLRELIEQYQHATNSHFQEINHDQNEMDKLQTGIRRYTGDDLSSLTLDDVSDLEQQLEYSVSKVRARKHQLLNQQLDNLRRKINENQQAALTGEVKLGEMTAPLAMLPPPAAFAHSTYYGGESSSSGTALQLMSAAPQLQHADLGFRLQPTQPNLQDPAAACGGLHGHGLQL
>Sspon.005D0006490	MGRGKVELKKIENPTNRQVTFSKRRMGLFKKANELAILCDAQIGVIIFSGSGRMYEYSSPPWRCVKHSFPFHIIHPFVHFHL*
>Sspon.005B0010621	MGRGKIVIRRIDNSTSRQVTFSKRRNGIFKKARELAILCDAEVGLVIFSSTGRLYEYASTSMKSVIDRYGRAKEEEQLVANPNTELK
>Sspon.005D0014413	MGRGKIVIRRIDNSTSRQVTFSKRRNGIFKKARELAILCDAEVGLVIFSSTGRLYEYASTSMKSVIDRYGRAKEEEQLVANPNTELKVCLKANNVLIKPTDPK
>Sspon.005A0012560	MGRGKIVIRRIDNSTSRQVTFSKRRNGIFKKARELAILCDAEVGLVIFSSTGRLYEYASTRALAAVEAAGPDRGEGVTAVLASEHGHGGGGESGGGERENEATAGMSCGETLPRHGRGSGGVEHEEEGEDDREGEEGHGEDEQYPGCMKSVIDRYGQAKEEEQLVANPNTELKRFLVDDRTEPIHSSKL*
>Sspon.007A0027942	MYSDLQGAFKWPKAMQPLLPKGHIPFKVDCYIKAGRVNACLFYLRGKQSQCLFATSAIAATSARPIKTVGAISEKTMVRGKVQMRRIENPVHRRVTFSKRREGLLKKARELSVLCGADVGVIIFSSTGKVHELATNGNMQSLVERYQSITARGNRALGFLVKGRDMSTAAWFKMKIMQQEIHFLKNKESILKSANENLQQKEGILKAANEVLQQKALVKTTQLHK

>Sspon.007D0021480	MYSDLQGAFKWPKAMQPLLPKGHIPFKVDCYIKAGRVNACLFYLRGKQSQCLFAMSAIAA<br>TSARPIKTVGAISEKTMVRGKVQMRRIENPVHRRVTFSKRREGLLKKARELSVLCGADVGV<br>IIFSSTGKVHELATNGNMQSLVERYQSITARGNRALGFLVKGRDMSTAAWFKMKIMQQEIH<br>FLKNKESILKSANENLQQKEGILKAANKFPTE
>Sspon.008A0002260	MGRGKIEIKRIENATNRQVTYSKRRTGIMKKARELTVLCDAQVAIIMFSSTGKYHEFCSPGT<br>ESVVLALLASSSSFICLLLIEFCRCLICVLILGCIKTIFDRYQQAIGTSLWNEQYENMQRTLSH<br>LKDINRNLRTEIRQRMGEDLDTLEFDELRGLEQNVDAALKEYHVITTQTETYKKKVKHSYE<br>AYKNLQQELGMREDPAFGFVDHTGAGGWDGAAAALGGGAPDMYAFRVVPSQPNLHGM<br>AYGSHDLRLG*
>Sspon.008D0001391	MGRGKIEIKRIENATNRQVTYSKRRTGIMKKARELTVLCDAQLAIIMFSSTGKYHEFCSPGT<br>DIKTIFDRYQQAIGTSLWNEQYENMQRTLRHLKDINRNLRTEIRQRMGEDLDTLEFDELRG<br>LEQNVDAALKEYHVITTQTETYKKKVKHSYEAYKNLQQELGMREDPAFGFVDHTGAGGW<br>DGAAAALGGGAPDMYAFRVVPSQPNLHGMAYGSHDLRLG*
>Sspon.008B0001180	MTDAWHGGLQRAESEPAGRRALTPTPRASDQAGQGGRQAEAFPSDGRGGRAVHGTTRHG<br>RARCPTPFTDPVAVSVHAAHCTSSSPPRNAHAHALSFHPPPYYHHQRLLSSAASHHSISSSSS<br>SSSFFSSHPPLRPSRSPCRRGRDPQPETTVERGGEQQQPAAEEDPRRRPETTPPTMGRGKIEIK<br>RIENATNRQVTYSKRRTGIMKKARELTVLCDAQPRDRVSSSCFACFLLFLHLLASDRVLSVP<br>DLRVNSWLVIARSIKTIFDRYQQAIGTSLWNEQYENMQRTLSHLKDINRNLRTEIRQRMGE<br>DLDTLEFDELRGLEQNVDAALKEYHVITTQTETYKKKVKHSYEAYKNLQQELGMREDPAF<br>GFVDHTGAGGWDGAAAALGGGAPDMYAFRV
>Sspon.008C0008910	MGRGKIEIKRIENATNRQVTYSKRRTGIMKKARELTVLCDAQVAIIMFSSTGKYHEFCSPGT<br>ESVLLALLASSSSFICLLLNDRVLSVPDLRVNSWLVIARSIKTIFDRYQQAIGTSLWNEQYEN<br>MQRTLSHLKDINRNLRTEIRQRMGEDLDTLEFDELRGLEQNVDAALKEYHVITTQTETYKK<br>KVKHSYEAYKNLQQELGMREDPAFGFVDHTGAGGWDGAAATLGGGAPDMYAFRVVPSQ<br>PNLHGMAYGSHDLRLG*
>Sspon.007A0009973	MGRGKIEIKRIENSTNRQVTFSKRRAGLVKKAREIGVLCDAEVGVVIFSSGGKLHDYCSPRT<br>SILEKYQTNSGKILWDEKHKILSAEIDRVKKENDNMQIQLRPLRVHFTSSLFVHLKGEDLNS<br>LQPRELIAIEEGLQNGQTNMRDKQAILLHPMDHWRMRKRNGKMLEDEHRMLSFRMHQQA<br>VDLSGGMRELEIGYHQVQHDRDFTSQMPFTFRVQPNHPNLQEDE*
>Sspon.007C0007880	MGRGKIEIKRIENSTNRQVTFSKRRAGLVKKAREIGVLCDAEVGVVIFSSGGKLHDYCSPRT<br>SLSRILEKYQTNSGKILWDEKHKILSAEIDRVKKENDNMQIQLRPMDHWRMRKRNGKMLE<br>DEHRMLSFRMHQQAVDLSGGLRELEIGYHQVQHDRDFTSQMPFTFRVQPNHPNLQEDE*
>Sspon.003C0008110	MGRGKIEIKRIENSTNRQVTFSKRRNGILKKAREISVLCDAEVGVVIFSSAGKLYDYCSPKTS<br>LSKILEKYQTNSGKILWDEKHKSLSAEIDRIKKENDTMQIELRHLKGEDLNSLQPKDLIMIEE<br>ALDNGLTNLNEKLMEHWERRVTNNKMMEDENKLLAFKLHQQDIALSGSMKDLELGYHP<br>DRDLAAQMPITFRVQPNHPNLQDNNYG*
>Sspon.003D0004890	MGRGKIEIKRIENSTNRQVTFSKRRNGILKKAREISVLCDAEVGVVIFSSAGKLYDYCSPKTS<br>LSKILEKYQTNSGKILWDEKHKSLSAEIDRIKKENDTMQIELRHLKGEDLNSLQPKDLIMIEE<br>ALDNGLTNLNEKLMEHWERRVTNNKMMEDENKLLAFKLHQQDIALSGSMKDLELGYHP<br>DRDLAAQMPITFRVQPNHPNLQDNNYG*
>Sspon.003C0008690	MGRGKIEIKRIENSTNRQVTFSKRRNGILKKAREISVLCDAEVGVVIFSSAGKLYDYCSPKTS<br>LSKILEKYQTNSGKILWDEKHKSLSAEIDRIKKENDTMQIELRHLKGEDLNSLQPKDLIMIEE<br>ALDNGLTNLNEKLMEHWERRVTNNKMMEDENKLLAFKLHQQDIALSGSMKDLELGYHP<br>DRDLAAQMPITFRVQPNHPNLQDNNYG*
>Sspon.002A0036730	MGRGRGKVEVRRIENSVSRQVTFSKRRRGLAKKARELAVLCDADVALLIFSDKGRFHDFA<br>AHGSMERILDRYERYLLCEARDVTEDYPEESQENMSYDHIKLRSKLEALQKSQRNLMGEQ<br>LESLTFREVQQLEHQIDSALRNIRSRKEAFLMEQNSILEKFLQLKKAKSPPELAGKNCSPDDS<br>NEPGRTVAAPPGAAISLPWWMLQPPGVSQRQEQEQH*
>Sspon.002D0038760	MERNCLERILDRYERYLLCEARDVTEDYPEESQENMSYDHIKLRSKLEALQKSQRNLMGE<br>QLESLTFREVQQLEHQIDSTLRNIRSRKEAFLMEENSILEKEKAALGAASLHTTNTAASSTA<br>VAAALPNLNFCPDDSDEPGRAMAAPPGAAISLPWWMLQPPTASQRQEQH*
>Sspon.005A0013481	MGRGKVELKKIENPTNRQVTFSKRRMGLFKKANELAILCDAQIGVIIFSGSGRMYEYSSPP<br>WRIESIFDRYLKAPSTRFEEMDIQQKIVQEMTRMKDERNRLRMIMAQYMGEDLASFSVQDL<br>SNLEQQIEFSLYKVRLRKQELLDQQLLEIRQREMHMPGEQSGYLCLMVRRLKTAGPSPDLL<br>KGQQQQAQAGEMVGNPRPFPWWDVGASGSGSGSQSLQLLPGRDAAESSMTALQLSPQLH<br>EYRLQPRQPNLQDANVHGWLW*
>Sspon.005A0003590	MCYEGLKYLKKGGPGRVSSSGGLIINISATLQYTAAWYQIHVSAAKAGVDSITRSLALEWG<br>TDYDIRVNGIAPGPIQDTPGMRKLAPEEMSKGKRETMPLFKLGEKWDIAMAALYLASDAG<br>KYVNGAIIIVDGGLWLSRPRHIPKEEVKALSKVVEKKGKVALVTGGGSGICFEIAAQLARH<br>GAQVAIMGRRREVLDKAVAALRSQGLRAVGFDGDVRKQEDAARVLAATVGHFGKLDILV<br>NGAAGNFLASPEDLTPKGFRTVLEIDTLGTYTMCYEALKYLKKDGPGKGPSTGGLIINISAT<br>LHYSASWYQIHVSAAKHHKITCSGMGTDYDIRVNGIAPGPIQDTPGVRKLAPEEMSKGLRE<br>MMPLFKFGEKRDIAMAALYLASDAGKYVNGTTLVVDGGLWLSHPRHIPKEEVRELSKVVE<br>KKIRISGVGVPSSKLIESIFDRYLKAPSTRFEEMDIQQKIVQEMTRMKDERNRLRMIMAQYM<br>GEDLASFSVQDLSNLEQQIEFSLYKVRLRKQELLDQQLLEIRQREMHMPGEQSGYLCLMNP<br>AIARGQQQQAQAGEMVGNPRPFPWWDVGASGSGSGSQSLQLLPGRDAAESSMTATALQL<br>SPQLHEYRLQPRQPNLQDANVHGWL
>Sspon.005D0006440	MGRGKVELKKIENPTNRQVTFSKRRMGLFKKANELAILCDAQIGVIIFSGSGRMYEYSSPP<br>WRIESIFDRYLKAPSTRFEEMDIQQKIVQEMTRMKDERNRLRMIMAQYMGEDLASFSVQDL<br>SNLEQQIEFSLYKVRLRKQELLDQQLLEIRQREMHMPGEQSGYLCLMNPAIARGQQQQAQ<br>AGEMVGNPRPFPWWDVGASGSGSGSQSLQLLPGRDAAESSMTALQLSPQLHEYRLQPRQP<br>NLQDANVHGWL
>Sspon.005D0006461	MGRGKVELKKIENPTNRQVTFSKRRMGLFKKANELAILCDAQIGVIIFSGSGRMYEYSSPP<br>WRIESIFDRYLKAPSTRFEEMDIQQKIVQEMTRMKDERNRLRMIMAQYMGEDLASFSVQDL<br>SNLEQQIEFSLYKVRLRKEMHMPGEQSGYLCLMNPAIARGQQQQAQAGEMVGNPRPFPW<br>WDVGASGSGSGSQSLQLLPGRDAAESSMTALQLSPQLHEYRLQPRQPNLQDANVHGWL
>Sspon.002A0032130	MPRRPRRSSIKYIENNNSRSISFSKRRDGLFKMAANLFTLTGARIAIVLEAENGKMSGFSAPS<br>FGPIMDSFLSGEEGGAPEGPDEQEKDMIAKLQKEIMQLEESKAMREQRAAESLARFKAIRES<br>SRVGKLIYSNVDDLCVDELNELLRGLARIGQEIRALLAPPPWVEIGGSLRGPPPLRLFSPPHS<br>QVQQPPRRLPWVSSQSQPSLPVLRSSCTLPESIRPQVSVMSPPVQPSSLQAISRMLLRHTQLS<br>QRPASLMVRPQAPVMMRLPNEAQQYNYQILGVDITGNTSGHFSQTSMLSSLPPPPPPLPTSA<br>LQIPSQRSVEVEVPFQALNLNPDVPSQSHANPHSILENDDEVSHFFGGIGGTTPTHSFTGNEQ<br>PSLIPCGAIGGHHAPGCHNVPCPSGSNGETYEWLSKTLLESSSEGGSSSDDDGAGDSLGNM<br>DWFGDDN*
>Sspon.002B0030700	MRMPRRPRRSSIKYIENNNSRSISFSKRRDGLFKMAANLSTLTGARIAIILEAENGKMSGFSA<br>PSFGPIMDSFLSGEEGGAPEGPDEQEKDMIAKLQKEIMQLEESKAMREQRAAESLARIKAIR<br>ESSRVGKLIYSNVDDLCVDELNELLRGLARIGQEIRALLAPPPWVEIGGSLRGPPPLRLFSPP<br>HSQVQQPPRRLPWVSTQSQPSLPVLRSSCTLPESIRPSSLQAISRMLLRHTQLSQRPTSLMVR<br>PQAPVMPLPNEAHQYNYQSLGVAITGNTSGHFSQTSLLSSLPPPPPPLPTSALQIPSQRSVEV<br>EVPFQALNLSPDVPSQSHANPHSILENDDEVSHFFGGIGGTTPTHSFTGNEQPSLIPCGANGG<br>HHAPGCHNVPCPSGSNGETYEWLSKTLLESSSEGGSSSDDDGAGDSLGNMDWFGDDN*
>Sspon.002D0029840	MPRRPRRSSIKYIENNNSRSISFSKRRDGLFKMAANLSTLTGARIAIVLEAENGKMSGFSAPS<br>FGPIMDSFLSGEEGGAPEGPDEQEKDMIAKLQKEIMQLEESKAMREQRAAESLARVKAIRE<br>SSRVGKLIYSNVDDLCVDELNELLRGLARIGQEIRALLAPPPPWVEIGGSLRGPPPLRLFSPP<br>HSQVQQPPRRLPWVSSQSQPSLPVLRSSCTLPESIRPSSLQAISRMLLRHTQLSQRPTSLMVR<br>PQAPVMPLPNEAHQYNYQSLGVDITGNTSGHFSQTSMLSSLPPPPPPPLPISALHIPSQRSVE<br>VEVPFQALNLNPDVPSQRHANPHSILENDDEVSHFFGGIGGTTPTHSFTGNEQPSLIPCGANG<br>GHHAPGCHNVPCPSGSNGETYEWLSKTLLESSSEGGSSSDDDGAGDSLGNMDWFGDDN
>Sspon.002A0032120	MKPKRPRRTGIHYVENDIDRSFTFIKRRDGLFKSAADLSILTGAKVAVVVESEREKMFAFGT<br>PSAGPIIDSFLSGNVECPIPHEVQKANIISLENKLVRLGNLKDVQDRRMRVSMARSKEIQESS<br>KIAKLVYGEIDDLSVDELNELLHGLTRINQEIEDHLRASQIQVPPRHLPWTSVQPPLQIPRSSS<br>AVPEPSWLQSSLLNSTTLPSPQAPPVPPLQHSLMPQHPWVADMVPLPNEAQQYNDQSQQL<br>VINGNPSGHFWLPSLLSSVPQPPPPPSSEHLPPEVELPFQALNLNPVLPPQSHAHSILENNDLS<br>LFTGSAEGNNYPADGSQFASYQWNSLSPSNDLYYGAFGGQDAGGHDMPGPSGLNQGTYD<br>WVSRPLLKDWAKVYLFDGIKVCMENRQRITLEQGCEWWWIHRLNPHRIRKVEGIALRERY<br>SLFEVYTAANIQDNDV*
>Sspon.002D0029800	MKPKRPRRTGIHYVENDIDRSFTFIKRRDGLFKSAADLSILTGAKVAVVVESEREKMFAFGT<br>PSAGPIIDSFLSGNVECPIPHEVQKANIISLENKLVRLGNLKDVQDRRMRVSMARSKEIQESS<br>KIAKLVYGEIDDLSVDELNELLHGLTRINQEIEDHLRASQIQVPPRHLPWTSVQPPLQIPRSSS<br>AVPEPSWLQSSLLNSTTLPSPQAPPVPPLQHSLMPQHPWVADMVPLPNEAQQYNDQSQQL<br>VINGNPSGHFWLPSLLSSVPQPPPPPSSEHLPPEVELPFQALNLNPVLPPQSHETNTHSILENN<br>DLSLFTGSAEGNNYPADGSQFASYQWNSLSPSNDLYYGAFGGQDAGGHDMPGPSGLNQG<br>TYDWLCRLLKDWAKVYLFDGIKVCMENRQRITLEQGCWLCNVEPMTGIAAVPLNASCKG<br>ETKGSFSTGLISLWESMTLRFLVSDRKADAWPNGPGHLRTGEDNWEELEGGLGRFEEE
>Sspon.002A0032080	MSRTPRRAGISYIRNNNSRRATFFKRRAGLYKAAADLSTLTGARIAIVLQSENGKMSSFGTP<br>SASVVANSFLSGDAQVDPFVNQVQKDKITLLQNELFMVEKEKAMEDKRTKENTARVKELC<br>ATSRKAKLVYSKVEDLNVEELSELLLDLSRVQREINDRLPPQQPSNQLEILLQKELEQHSDD<br>GPGNNLVDMGCLDDS
>Sspon.006B0021670	MSRTPRRAGISYIRNNNSRRATFFKRRAGLYKAAADLSTLTGARIAIVLQSENGKMSSFGTP<br>SASVVANSFLSGDAQVDPFVNQVQKDKITLLQNELFMVEKEKAMEDKRTKENTARVKELC<br>ATSRKAKLVYSKVEDLNVEELSELLLDLSRVQREINDRLPPQQPSNQLEVGGSRDPLLWLLS<br>SSTSHSKNLLPPRRLPWTHTQPSLHVAKSSLSLPQSSWSQATLLNQTILPTAQAPSTSLLQHG<br>LMPPHPLMVKPQAQMLSSSNEAHQHNYNNTRVHINSNILQPSSQSSLISMLQPPPSSLAQITF<br>YNEIPPPSLLQQPPTLLPPEPELHPAAQPENHASIGDFTAAHPFPIHQWPSPIQSDWPYFDA
>Sspon.003B0017430	MMPRRDRRSGVRFIEDGRDRSLTFFKRRSGLFKAASDLSTLTGARVAVNEVFQLEKDKTM<br>EDKRKKESMARTNEKIQEASKMAKYVVYGNIEDLDATELFDMYRELSRVKQEINDRLPTLL<br>HEDKVEAGGRLRDPSLLQPTWWRSMPPPQVATPPKYPWTPFQACFQQHPWLSTSASVLAT<br>SGSSLPNSVIHPSPQVPQNPLQHYYPLAPHTPSSVQFQAPKTPPAPMEAAHNPYSYHIYGIDI<br>NGNSSHPFSLSPILSSSTTPQPSSLQTTPPSDDSSPLSLSPQISSPLHLGSSSQQLPFSVQNYNT

	MQPPQHYANVGSTSIHSHQLFYSNLSSPELNVELGNIDKSGGTQVGGGHNERFGLSSPQQS<br>DGGFDGVMPKSFSAGESSGGGHAGGNLGGL
>Sspon.003B0017420	MMPRRDRRSGVRFIEDGRDRSLTFFKRRSGLFKAASDLSTLTGARVAVVLESEHGKFSSFG<br>TPEASPILDAFLLGSAPTDLDTSEAEKASITNLQNEVFQLEKDKTMEDKRKKESMARTNEKI<br>QEASKMAKYVYGNIEDLDATELFDMYRELSRVKQEINDRLPTLLHEDKVEAGGRLRDPSL<br>LQPTWWRSMPPPQVATPPKYPWTPFQACFQQHPWLSTSASVLATSGSSLPNSVIHPSPQVP<br>QNPLQHYYPLAPHTPSSVQFQAPKTPAAPMEAAHNPYSYHICGTDINGNSSHPFSLSPILSSS<br>TTPQPSSLQTTPPSDDSSPLSLSPQISSPLHLGSSSQQLPFSVQNYNTMQPPQHYANVGSTSIH<br>SHQLFYSNLSSPELNVELGNIDKSGGTQVGGGHNERFGLSIPQQSDDGFDGVMPKSFSAGES<br>SGGGHAGGNLGGLKFPWY
>Sspon.008D0009720	MGRPSKGRQRIEIRRIEDAGRLEVTFSKRKSGLQKKASELFLLCGSPVALVVFSPGKKAFAL<br>GTPSVDDVLRRYAPVPGEELDAKLLAVLQDTDDASAVADRAEAEAIVRRTEDTRARSATE<br>KARMDAIGKSVRQAAAKAGRKFWWEADSDELGEAELPEFVKVLRRLRVNLQRHLDSLSA<br>RLHFPDAEDNLTFNDTMIALRLMRTQFPKLDKVVAQPFILQSQLYSSVKDRTQVDRDLESF<br>KKDKVLRIFKLSSGQDDHAIMFMDDYLKQVAFAIKRSGGKDQDSSEVFEWFERYVVPSKL<br>DVSINQIELTRQLIDPNIYWFSIPRIGPILKGLSQGRKEILSLLNRRKYKEMLLSSLENTKLRLS<br>PLDTRFLLRDLIGSGHIKTVQTPTGLLARISRD*
>Sspon.005C0001960	MHYRPARRVEEEEQTGRRQATEARCRGDSQTYQNFSVSSYVAAAELLSSCSSRTENQRSPD<br>TETPFLCRLIAIERAFLSGIFSAVEEGGRKPEKQDTRRTATTMGRPSKGRQRIEIRRIEDAGRL<br>EVTFSKRKSGLQKKASELFLLCGSPVALVVFSPGKKAFALGTPSVDDVLRRYAPVPGEELD<br>AKLLAVLQDKDDASAVADRAEAEAIVRRTEDTRARSATEKARMDAIGKSVRQAAAKAGR<br>KFWWEADSDELGEAELPEFIKVLSRLRVNLQRHLDLLSARLQ*
>Sspon.008B0010391	MGRPSKGRQRIEIRRIEDAGRLEVTFSKRKSGLQKKASELFLLCGSPVALVVFSPGKKAFAL<br>GTPSVDDVLRRYAPVPGSELDAKLLAVLQDTDDASAVADRAEAEAIVRRTEDTRARSATE<br>KAQMDAIGKSVRQAAAKAGRKFWWEADSDELGEAELPEFVKVLRRLRVNLQRHLDSLSA<br>RLHFPDAEDNLTFNDTMIALRLMRTQFPKLDKVVAQPFILQSQLYSSVKDRTQVDRDLESF<br>KKDKVLRIFKLSSGQDDHAIMFMDDYLKQVAFAIKRSGCSLLSQGGDVTDQHITLLMNAG<br>LLGRKEILSLLNRRKYKEMLLSSLENTKLRLSPLDTRFLLRDLIGSGHIKTVQTPTAVNGSR<br>MHVLMLPWLAFGHILPFTELAKRIARQGHSVTFLSTPRNTRRLIDVPPDFASLIRVVDVSLPP<br>VEHLPEGAEAIIDLPSDDLRPCLRRAYDAAFFPALLDILQAPKTSRPDWVLTDYATYWAPQ<br>AAARHGVPCAYLSLYGAAVLSFFGPPEALMGRGRHAKTAPEHLTEVPDFVPFPTTIAYRGY<br>EARQMFKPAVVPDVSGVAELYRSGMSIDGSQVVGIRSSRELEPEWLQLLGELYQKPVIPVG<br>FFPPPPTQDVVAGHEATLRWLEEQAPGSVVYAAFGSEAKLTSAQLETIPLGLEASCLPFLWA<br>FRAPGVANNSRN*
>Sspon.008A0006430	MLKPGKKTSRGRQKIEIRRIEKKESRQVTQCKRKGGLLKKASELHLLCGAHVAIIVFKRTEP<br>EGKEVPVARRGKRDDGSIAFAMGTPSVDHVLRRCALLHAGEGLTAVEDVGAVAAERAVM<br>EARARETEQTRALVEAEKARNDAVGEKVLQAVEVSGRRFWWEVDVGVLGEAELPVFTSQ<br>LQRLRESVQLQANKSQTSATPAA
>Sspon.008D0010450	MLKPGKKTSRGRQKIEIRRIEKKESRQVTQCKRKGGLLKKASELHLLCGAHVAIIVFKRTEP<br>EGKEVPVARRGKRDDGSIAFAMGTPSVDHVLRRCALLHAVEGLTAVEDVGAVAAERAVM<br>EARARETEQTRALVEAEKARNDAIGEKVLQAVEVSGRRFWWEVDVGVLGEAELPVFTSQL<br>QRLRESVQLQANKSQTSATPAAVAAPWHQLG*
>Sspon.008D0020380	MVKTAGKKATSKGKQRIQIEYIEDKEKRQVTFSKRKGGLLKKASELHLLCGAHVAIVIFSQ<br>RQDANPPIEAPPGGRRSSTRRRGGNVFAMGAPSVDHVMSRLAVVAPLAAAGDPYHRAALE<br>DDAARAGREGAEATARRRDEARELVDAEEARMKAVADKVRLATAVGRSWWEADVEAL<br>GEAELPEFGGALKRLRDNVLRRHADKLVPQ
>Sspon.007C0011450	MVSPRRTGKKSQGRRRKEMVLIEDPKSRLVTFSKRKSGFFKKASELSLLCGARVAAVVFSA<br>TGKPSAVGAPSVERVISRFAPLPSGGDDDREREVMEATVRRAKETGARVAEEKTRMDAVG<br>EKVLRAAAADGVRFW
>Sspon.007D0011150	MVSPRRTGKTSQGRRRREMVLIEDPKSRLVTFSKRKSGFFKKASELSLLCGARVAAVVFSA<br>TGKPSAVGAPSVERVISRFAPLPSGGDDDREREVMEATARRAKETGARVAEEKTRMDAVG<br>EKVLRAAAADGVRFWWQADAEALGEEELREFARKLRRLRDNVRRCADKLQVAPASQPM<br>TWLQGLREGRPAMTEVTVVTVGSRRSFVETLPLCGGAVASGGMSGGGR*
>Sspon.004B0022920	MQVTAASSASMASEAEMVGEAGGGGERKRKKTLGRRKIDIKPIKCMEARHVCFSKRREGL<br>NKKASELCALTGAKVAVIVYSPAGKPYSFGHPSVSAVVDRYLDHDPVSSAAANDAFEAPPP<br>PMTYEFDGQRDRLCEAIAAEARRKDTLDAAARAAGVWTDDVVRQAEMPELVAMLAALE<br>RVKDDADHAMRQHQCAAAAAAGACDACYYDLGDGTFAADDYGGASSSSHHHQQAAMD<br>AQTMALLMGSSAVGHAAAHAPMLLPPPDLPPPTVAPVPLAFNYGSDHNHITGYEGYAYDL<br>GDGGGYGHGGAAFETEGCYFGPTATCNFFG*
>Sspon.004C0020411	MQVTAASSASMASEAEMVGEAGGGGERKRKKTLGRRKIDIKPIKCMEARHVCFSKRREGL<br>NKKASELCALTGAKVAVIVYSPAGKPYSFGHPSVSAVVDRYLDLDPVSSAAANDAFEAPPP<br>PMMYEFDGQRDRLCEAIAAEARRKDALDAAARAAGVWTDDVVRQAEMPELVAMLAALE<br>RVKDDADHAMRQHQCAAAGAAAGACDACYYDLGDGTFAADDYGGASSSSHHHQQAA<br>MDAQTMALLMGSSAVGHAAAHAPMLLPPPDLPPPTVAPVPLAFNYGSDHNHITGYEGYA<br>YDLGDGGGYGHGGAAFETEGCYFGPTATCNFFG*
>Sspon.004A0022300	MQVTAASSASMASEAEMVGEAGGGGERKRKKTLGRRKIDIKPIKCMEARHVCFSKRREGL<br>NKKASELCALTGAKVAVIVYSPAGKPYSFGHPSVSAVVDRYLDTTPSPPPPPTTRAAAADD<br>VRVRRPARPAVRGHRGGGAAEGHARRGGARGRVWTDDVVRQAEMPELVAMLAALERV<br>KDDADHAMRQHQCAAAAAAAGACDACYYDLGDGTFAADDYGGASSSSHHHQQAAMDA<br>QTMALLMGSSAVAHAAAHAPMLASS*
>Sspon.003C0035970	MAMAKEVERVGEAGGDRRRKKTMGRQKIEMKPIQCPEARHVCFSKRRVGLLNKATELCA<br>LTGAQLAIVVFSPAPAGKPYSFAHPSVNAVIDRYCDPSAAAAASTEATTRPTILCGGFDSE<br>CERLRKAIAAEARRRDELDAAARAAGVWTSDDVRGWGMPGLVAMLAALERVQAEAAER<br>AHEMFTDEEAMVQWYAATTGVFSDAFDYDLGGSVTALSRLTVVPAAVTMEAWTRRR*
>Sspon.003A0028120	MEVETHRKFKLPSKSNRIPCFHYFLPPPSPDPRAPSPAAALRLLRPPRRPPRSLLRPPRVPRRS<br>PYPWLLLPPAAIPAPAPRLPPPRPSSATRRSPPPRLWKLRPPPCATPTRRAAPNSRRRQTSSGP<br>PPPPRSGSALLPPLSTWSASRPTCWQQAAALRARLHLEHHRDDDSEGDADSISTETATVAH<br>LHSQAAAVQNIKNLIPIVLDLQASNYSKWRGYILLILGRLALKDHVLSDAIHPHDAAWSRM<br>DYVVVSWIFNTIAPDLLDVVHERDGITARAASLGLEQQFFNNRESRAMLLDAEFRTLCQGA<br>LSVDEYCRKMKNMADALADLGEPVLDRTLVLNVLRGLNERFQFMSQLVTRQKPFPSFAD<br>VRAYLRLAELNMASPLAPPSTLITAPSSKPPAPSPAPQRPPQPARGQQLGHTPAPPRHPQAA<br>GGQQSGNDNNRGRRHRGGRGQGDVARVQPRRLSRSTAPTCLHLHRHNKLSWPGRPPGYF<br>APPPVPGAYYQAPQQAPTSTWSPSPWTTEGLANAFSTVTLTPPSSTSDRVFDSGASSHIAGT<br>PGMVTMSPTSSFPSSIVVGNGATLPVVGTGYSTLPGPFRLNNVLIAPDIIRNLLSIRQFTTDNL<br>VYVEFDPLGIYVKDLRTRNLLLRSLLSPLRAHSKVYVAAMAMAKEVERVGEAGGDRRRK<br>KTLGRQKIEMKPIQCPEARHVCFSKRRVGLLNKATELCALTGAQLAIIVFSPAPAVNAVIDR<br>YRDPSAAAAASTEATTRPTILCGGFDSERERLRKAIAAEAGRARRCGARGRRVDQRR
>Sspon.003B0032610	MAMAKEVERVGEAGGDRRRKKTLGRQKIEMKPIQCPEARHVCFCKRRVGLLNKATELCA<br>LTGAQLAIVVFSPAPAGKPYSFAHPSVNAVIDRYCDPSVAAAAASTEATTRPTTLCGGFDSE<br>CERLRKAIAAEAGRRPCGAPGRRVNQQR
>Sspon.007A0002050	MGRQKIEIRRIESDEARQVCFSKRRAGLFKKASELSILCGADVAAVVFSPAGKAFSFGHPSV<br>ESVVERFLASSSSPSPAGAGAGHSSAGGGEDRAVSELNRQHGELRAQLDAEKTRQERADE<br>AIRKEREARSPAMAWIDADLGAMGHDDLVAFWAALAGVQATVAASADQLLRDALLVGR<br>RGRHQPPPAQLAGGGVAFDVGAFGIGVQVQPPPGFAGVDLQGFGGQAAAILGAAGPS*
>Sspon.007D0001670	MGRQKIEIRRIESDEARQVCFSKRRAGLFKKASELSILCGADVAAVVFSPAGKAFSFGHPSV<br>ESVVERFLASSSSSSPSPAGAGAGHSSAGGGEDRAVSELNRQHGELRAQLDAEKTRQERA<br>DEAIRKEREARSPAMAWIDADLGAMGHDDLVAFWAALAGVQAAVAASADQLLRDALLV<br>GRRGRHQPPPAQLAGGGVAFDVGAFGIGVQVQPPPGFAGVDLQGFGGQAAAILGAAESPK<br>*
>Sspon.007B0001241	MGRQKIEIRRIESDEARQVCFSKRRAGLFKKASELSILCGADVAAVVFSPAGKAFSFGHPSV<br>ESVVERFLASSSSSSPSPAGAGAGHSSAGGGEDRAVSELNRQHGELRAQLDAEKTRQERAD<br>EAIRKEREARSPAMAWIDADLGAMGHDDLVAFWAALAGVQAAVAASADQLLRDALLVG<br>RRGRHQPPPAQLAGGGVAFDVGAFGIGMQVQPPPGFAGVDLQGFGGQAAAILGAAGPS*
>Sspon.006B0011260	MVKDKSTKGRQRIEMKSIEGEVARQVCFSKRRPSLFKKASELSTLCGADVAVVTFSPGGKC<br>FSFGHPSTSSVADRFLAVHTFDGLTMGSGSHGSQGSTGTSHEMNQQVMELQQLMETEKRR<br>KERAVEAMERESGGPVMKLLNANVGALGIHELEELRKELCMVQNMVKERSREMLEDAM<br>QTRRLPPQSQMHMVAMPSQVLSGGQSAGTMYTTFPSLSNGPREGLHVNSPLHGSLGGLGN<br>YLNGQFGG*
>Sspon.006C0011810	MVKGKSTKGRQRIEMKSIEGEEARQVCFSKRRPSLFKKASELSTLCGAEVAVVTFSPGGKC<br>FSFGHPSTSSVADRFLAVHTFDGLTMGSGSHGSQGSTGTSHEMNQQVMELQQLMETEKRR<br>KERAVEAMERESGGSVMKLLNANVGALGIHELEELRKELCMVQNMVKERSREMLEDAM<br>QTRRLPPQSHMHMVPSQVLSGGQSAGTMYTTFPSLSNGPREGLHVNSPLHGSLGGLGNYL<br>NGQFGG
>Sspon.006C0011680	MVKGKSTKGRQRIEMKSIEGEEARQVSFSKRRPSLFKKASELSTLCGAEVAVVTFSPGGKC<br>FSFGHPSTSSVAYRFLAVHTFDGLTMGSGSHGSQGSIGTSHEMNQQVMELQQLMETEKRK<br>KEGAVEAMERERIHELEELRKELCMVQNMVKERCHANKKAATTISYAYGALASIVWWPE<br>CRNHVYHFPKFEQWPT*
>Sspon.006B0011280	MVKGKSTKGRQRIEMKSIEGEEARQVCFSKRRPSLFKKASELSTLCGAEVAVVTFSPGGKC<br>FSFGHPSTSSVVDCFLAVHTLGGHTMRSGSHGSQGSTGRSHEMNQQVMELQQLMEIEKKR<br>KERAVEALERERGGPVMQLLNANVGGALGIHELEELRKELCMVQDMVKQRSRKVLQDAM<br>QTRRLPPQSHMHMVAMPSQVLFGSLRVGTMYTTFPSLSNGPREGLLINSLLHGAPALGATE<br>PSKKPRTHLVLSFTQGLLKLTDLFRWCDCVILCTKNLNWPFDLCGFGGH
>Sspon.002B0025700	MCLALPLPHNDRGKAAMPRRKIEIKRIENEDARKVCFSKRRHGLFKKASELSILCGATVGS<br>VVFSNSGRSFSFGHPSINDVADRFLNSVAPGDLASGGASHDNSGAVTDTAHRLNMELLELQ<br>HALDSENKKKERLQEAIEKENGEHMMQWLNANVLELGLAELQEFQKCLEAMDSAVKEKT<br>NQILLEASQITQGSEPQLPMEIASTSRYQFGQHISADPMALADPSSSNGFIDGFEVNDPLRSG<br>GLQDVCGLQNFPNNQNQEKSCIQWEDNVLVASREPGQAWCGQPIRPLMLDVDHMIVANK<br>SLMEENRRVNAERLDVDRVMGAC*
>Sspon.002B0025660	MAAPGACGPSASQHDVGAGPAVERPPSEVGGRENPPRVGLLGVVEECLTTCRLPTAGGAR<br>PALAMKEEAEDDGADADGDEDGTPPLPCPSPRQELATPRRTHRHRPWSRTRTTSPAVQGT

	AMRSLATIGGTSMMRRQKGGCEGAAPMVTRVRSAAVAAASSGGKLKSESEEDIRTKGEAL<br>VWGGPRGRAATGVFWWTLGPERPPAGLMLFYSCFTAPHSSVRSEVVNLGKAAMPRRKIEI<br>KRIENEDARKVCFSKRRHGLFKKASELSILCGATVGSVVFSNSGRSFSFGHPSINDVADRFL<br>NSVAPGDLVSGGASHDNSGAVTDTAHRLNMELLELQHALDSENKKKERLQEAIEKENGEH<br>IMQWLNANVLELGLAELQEFQKCLEAMDSAVKEKTNQILFEASQITQGSEPQPPMEIASTSR<br>YQFGQHISADPMALADPSSSNGFIDGFEVNDPLRSGGLQDVCGLGNFPNNQNQEKSCIQWE<br>DNVLYIANYRMHLIVSIHKQNNHQQVASKEPGQAWCRQPIRPLMLDVDHMIVANKSLMEE<br>NRRVNAERLDASTESWILLILENRKEN*
>Sspon.002C0028270	MVKGKAAMPRRKIEIKRIENEDARKVCFSKRRHGLFKKASELSILCGATVGSVVFSNS
>Sspon.002D0024620	MVKGKAAMPRRKIEIKRIENEDARKVCFSKRRHGLFKKASELSILCGATVGSVVFSNSGRSF<br>SFGHPSINDVADRFLNSVAPGDLASGGASHDNSGAVTDTAHRLNMELLELQHALDSENKK<br>KERLQEAIEKENGEHMMQWLNANVLELGLAELQEFQKCLEAMDSAVKEKTNQILLEASQI<br>TQGSEPQPPMEIASTSRYQFGQHINANPMALADPSSSNGFIDGFEVNDPLRSGGLQDVCGLG<br>NFQNNQNQG
>Sspon.002A0028870	MVRRKIEIKPIENENSRQVCFSKRRQGLFKKASEISILCGAMVGSVVFSSFGKSFSFGHPSIDD<br>VVNRFLNSVTPDGPASSGANHDNSLAVTGTVQGLNMEYLELQQSLDSQKKKKERLQEATK<br>KEMGERMMQWLNANILELSLDELQEFQKLLEAIDGVVKEKENNIVVEARETEGSATQPPM<br>EIASALQYQFGEHISANSMAFTAPSSSNGFIDGFEVNDPLLSGGLQDVCGLGNFPYNQNH
>Sspon.002C0028250	MVRRKIEIKPIENENSRQVCFSKRRQGLFKKASEISILCGAMVGSVVFSSFGKSFSLGHPSIDD<br>VVNRFLNSVTPDGPASSGANHDNSLAVTGTVQGLNMEYLELQQSLDSQKKKKERLQEATK<br>KEMGERMMQWLNANILELSLDELQEFQKLLEAIDGVVKEKENNIVVEARETEGSATQPPM<br>EIASALQYQF
>Sspon.002B0025690	MVRRKIEIKPIENENSRQVCFSKRRQGLFKKASEISILCGAMVGSVVFSSFGKSFSFGHPSIDD<br>VVNRFLNSVTPDGPASSGANHDNSLAVTGTVQGLNMEYLELQQSLDSQKKKKERLQEATK<br>KEMGERMMQWLNANILELSLDELQEFQKLLEAIDGVVKEKENNIVVEARETEGSATQPPM<br>EIASALQYQFGEHISANSMAFTAPSSSNGFIDGFEVNDPLLWWSPRCLWPGEFPLQPESWLIS<br>TN
>Sspon.002D0024610	MAGDGAGRCSGAWGDGDRLSILHVAHIIFDQPSASEDYIYILVHEQYTIVLIYIVQQLMVKD<br>GKANYYHGVPEQLGKGEIKPIENEETRQICFTKRRQSLFNKASELSILCGAMVGSVVFSTAG<br>TSFSFGHPSIDDVANRFLNYSVTSDGPASSGESNDYSWAVPDTIQPLNMEYSELQQALVSEK<br>KKNEMLQEATKKEMGEPMMQLLNTNISELSLEELQEFQKYLDAIHGVVEEKDTKMPETSQ<br>PQGLVPQPPMDIAPDLQYQFGDEHISANPMAFTAPSSSDIEFVDGLFEVNDPCLNGDFADGQ<br>IGIFDHTMPRSVTASQSPQTLDNYSKV*
>Sspon.002B0025650	MVKGEIKPIENEETRQICFTKRRQSLFNKASELSILCGAMVGSVVFSTAGTSFSFGHPSIDDV<br>ANRFLNYSVTSDGPASSGASNDYSWAVPDTIQPLNMEYSELQQALVSEKKKNEMLQEATK<br>KEMGEPMMQLLNTNISELSLEELQEFQKYLDAIHGVVEEKDTKMPETSQPQGLVPQPPMEI<br>APDLQYQFGDEHISANPMAFTAPSSSDIEFVDGLFEVNDPCLSGDFADVCGLGNFPDNQNH<br>GYCHGILH*
>Sspon.002C0028240	MVKGEIKPIENEETRQICFTKRRQSLFNKASELSILCGAMVGSVVFSTAGTSFSFGHPSIDDV<br>ANRFLNYSVTSDGPASSGASNDYSWAVPDTIQPLNMEYSELQQALVSEKKKNEMLQEATK<br>KEMGEPMMHLLNTNISELSLEELQEFQKYLDAIHGVVEEKDTKMPETSQPQGLVPQPPMEI<br>APDLQYQFGDEHISANPMAFTAPSSSDIEFVDGLFEVNDPCLSGDFADVCGLGNFPDNQNH<br>DCMGSVRY*
>Sspon.006D0023110	MGRVKLKIKRLENSSGRQVTYSKRRSGILKKAKELSILCDIDLILLMFSPTGKPTICIGERSNI<br>EEVIAKYAQLTPQERAKRKLESLEALKKTFKKLDHDENFAKQHLIGLQCAAAQFQTDMQL<br>PLGLTGEPGPSSWFQNGGADGQQAMMLPDDSSLLHQRDIAGCSTSTSLQGYPGYFSMAKQ<br>ETDTGGGTRAPAVHQPPPPPPDFSQAECLTSLHLGAQFPYAPCFDHPNLFNDRMFRPDAVE<br>LHDGSAGMDFGGGHFDLPRPGDEASFQNWASAAACGAAMFDHHHQQQQHHQQPQSSS<br>AQ
>Sspon.006B0023900	MGRVKLKIKRLENSSGRQVTYSKRLSGILKKAKELSILCDIDLILLMFSPTGKPTICIGERSNI<br>EEVIAKYAQLTPQERAKRKLESLEALKKTFKKLDHDENFAKQHLIGLQCAAAQFQTDMQL<br>PLGLTGEPGPSSWFQNGGADGQQAMMLPDDSSLLHQRDIAGCSTSTSLQGYPGYFSMAKQ<br>ETDTGGGSEHGPPAVHQPPPPPPDFSQAECLTSLHLGAQFPYTPCFDHPNLFNDRMFRPDAV<br>ELHDGSAGMDFGGGHFDLPRPGDEASFQNWASAAACGAAMFDHHHQQQQHHQQPQSS<br>SAQ
>Sspon.006D0023120	MGRVKLKIKRLENSSGRQVTYSKRRSGILKKAKELSILCDIDLILLMFSPTGKPTICIGERSNI<br>EEVIAKYAQLTPQERAKRKLESLEALKKTFKKLDHDENFAKQHLIGLQCAAAQFQTDMQL<br>PLGLTGEPGPSSWFQNGGADGQQAMMLPDDSSLLHQRDIAGCSTSTSLQGYPGYFSMAKQ<br>ETDTGGGSEHGQPAVHQPPPPPPDFSQAECLTSLHLGAQFPYAPCFDHPNLFNDRMFRPDA<br>VELHDGSAGMDFGGGHFDLPRPGDEASFQNWASAAACGAAMFDHHHQQQQHHQQPQS<br>SSAQ
>Sspon.006B0022030	MARKKVNLQWISNNATRRATYKRRSQGLEKKASELATLCGTKLCVVTYGQGEAQPKVWP<br>SDEEAKELLKKFNSMLDVSSLKKTKNQEEFLHSRSVRLHEQVSKLELENRERETLDLLHDS<br>MYGGRPGLVGTNKDELLSLCEMVEMKMRKIKARLQQLVVGQGVLPKPLPQMMLPASSAL<br>QTQASCYTYNEMQSMALLEEHRHLQPAWRLANVAPNYSQFA
>Sspon.006D0021300	MARKKVNLRWISNNATRRATYKRRSQGLEKKASELATLCGTKLCVVMYGQGEAQPKVW<br>PSDEEAKELLKKFNSMLDVSSLKKTKNQKEFLHSRSVRLHEQVSKLDLENRERETLDLLHE<br>TMYGGRPGLVGTNKDELLSLSEMVEMKMRKIKARLQQLVIGQGVLPKPLPQVMLPASSSL<br>QTQASYYTYNEMQRVALPEEHRHLRPAWRLASVAPNYSQFAGVLLLIHVLNS*
>Sspon.006D0021280	MARRKANLQWISINATRHATYKRRSQGLEKKASELTTLCGIKLCVVVYGEGEAQSKVWPS<br>DEEAKQLLMKFNSMLDVRSLKKTKNQEEFLRSRSVRLHEQTLDLLHDTMYGGRPGLVGSN<br>KDKLLSLSEMVEMKMRKIKTRLQQLVVEQGVLPQPLPQVMLPVSSSLQTQASCYTYNEMQ<br>SMAPKQAPRVAGPWVHHWYGGDAYGCHSFCLRHHFWPLLFFIAHPARVWISWTIDIAHK<br>CLLPAVF*
>Sspon.006D0021310	MEMKGQATPQEIQGHMLDVGSFKKIQNQEGFLHSRTASSLYTYNEMQIMAPIEEHQLQQD<br>WPIASPGPNIGEIGTVLNSVFVGSSRDASGSGCEMTQPYNLDWRSPKVGGEMFDLITECGIL<br>GPQEKLRSSPAIPTLNGAEHCPTRDRSRTTRLNHSTFVINSHRRYQSLVKKASELTTLCGIKM<br>CVVVYGDSKAQPEVWPSDAEAKKFLKKFKDMPNIGSFKKTQSQEEFLQSRTVRLHEQATK<br>LDHENRERETLVLLHDSLDGRRPGLGGTTKDELISLREMVEMKMSKAKARIQQFVGGQGA<br>LPDPLQVMLPVSSSSQTQASSYTYNEMQIMAPLEEHQLQQDWPLASLAPNYGELGAVVYG<br>GFVGSSRDPSGSGCEMMQPYNLGSSSGWRKMFKFMSKQFETNSQWAWLMVCTPERVTM<br>SSVLMPLAAKKFTILRSSENGVS*
>Sspon.006C0021980	MARKKVNLQWISNNATRRATYKRRYQSLVKKASELTTLCGIKMCVVVYGDSKAQPEVWP<br>SDAEAKKFLKKFKDMPNVGSFKKTQSQEEFLQSRMVRLHEQASKLHHENRERETLVLLHD<br>SLDGRRPGLSGTTKDELISLREMVEMKMSKAKARIPQFVGGQGALPDPLQVMLPVSSSSQT<br>QASSYTYNEMQIMAPLEEHQLQQDWPLASLAPNYGELGAVVYGGFVRSSRDPS
>Sspon.003B0025330	MARKKVNLRWITKASSRPATYKRHCNSMMKKVDELATLCGAKVGVVLYGENQIKPLSWP<br>NDSMVKDLPKIHQHAKFWQKIQEDTKSGGTSQQLDPKLQWQVSRMENENYKHEIMFLLY<br>EIMDGCRPGLIGTTNKECTSHGEMVERTKKVEELIQQLQLGIWQGKRVALDPPLPLQIALSS<br>TQPSLHPTPSMRYKSH
>Sspon.003B0025320	MSATRFPSRGYMYNCIVNARIMARKKVNLQWITKASSRRATFKRRRDGIKKKVDELATLC<br>GTKVGVVLYGENQDKPLAWPNDSEAKAMFQKFIDMPDCGKRFKKTQNQEELLSNRIPKLQ<br>HQVSRLEHENYKREISFLLYESMDGRRPSLIGTSEKERTSLGEMVEEKTKKVKERIQQLRQL<br>GIWQGKGAAREPPLPLQLGSSSAQPLAPYTFNEIQTSLVPAVGHVPPQQQDWIASLTTNGGE<br>LGAIVSRPFVGSSGGVDMMHPYNMDYFSRWKDTPPLHPAVKETTTLVHLMIWR*
>Sspon.004A0013490	MARKKVTLHRIANDSTRRGTFKKRRKGLMKKASELATLCDVDTCVVVYGEGESQAEVWP<br>DVPTAEHVLARFKAVPELDQCKKMLDMESFLRQRMDKLREQLHRAQRDNREREATLLLH<br>DAIVGRRPGLVGLSVEEIASLGCMVESRLNGVKDAIGRLQRMGQEVPATVAAALQPQLQA<br>PSSMPLMPAYRAGTTGHRDMMTMQVPVPHPQPQGWLVSGGDLGALVHGGGFGGDMNM<br>MMPQFGNMAVGFAWSDPGQYFHSM*
>Sspon.004D0015380	MARKKVTLHRIANDSTRRGTFKKRRKGLMKKASELATLCDVDTCVVVYGEGESQAEVWP<br>DVPTAEHVLARFKAVPELDQCKKMLDMEGFLRQRMDKLREQLHRAQRDNREREATLLLH<br>DAIVGRRPGLVGLSVEEIASLGCMVESRLNGVKDAIGRLQRMGQEVPATVAAALQPQLQA<br>PSSMPLMPAYRAGTTGHRDMMTMQVPVPHPQPQGWLVSGGDLGALVHDGGFGAGTSAG<br>GDMNMMMPQFGNMAVGFAWSDPGQYFHSM*
>Sspon.001A0027930	MARKKVTLQRIANDSTRRATFKKRRKGLMKKASELATLCDVDACIVVYGEGETQPEVWP<br>DVSKVAQVLARFKAMPELDQCKKTMDMEGLLTQRIDKQKEQLHKAWRENRERETTLLLH<br>DAIVGRRPGLAGLSVEDIAGLGWMVENRLVVVNESLERHHAAAAGAGKQQQDNVVVPPT<br>TLQLQMPQQVSLPLVPPYSIGGPTGGQTAVVHQAPPPNPQPQPHPQASWLMELARAGGDL<br>GALVYSGFGGGRGGCFGGSAGAGTSAGAADMLPHLGNFGAGFGWPFCFLSDVRRRSVRH<br>PSFLGSPQFLDATMLARHAACVAANLIQHATPRG*
>Sspon.001C0027140	MARKKVTLQRIANDSTRRATFKKRRKGLMKKASELATLCDVDACVVVYGEGETQPEVWP<br>DVSKVAQVLARFKTMPELDQCKKTMDMEGLLTQRIDKQKEQLHKAWRENRERETTLLLH<br>DAIVGRRPGLAGLSVEDIAGLGWMVENRLVVVNESLERHHAAAAGAGKQQQDNVVVPPT<br>TLQLQMPQQVSLPLVPHYSIGGPTGGQTAVVHQAPPPNPQPQPHPQASWLMELARAGGDL<br>GALVYSGFGGGRGESFGGSASAGTSAGAADMPPHLGNFGAGFGWPDPAGADPSAFPP
>Sspon.001D0028140	MARKKVTLQWIANDATRRATFKKRRKGLMKKASELATLCDVDACVVVYGEGETQPEVW<br>PDVAKAAEVLARFRAMPELDQCKKMMDMEGFLKQRIDKLKEQLHKARRENHEREVTLLL<br>HDAIVGRRPGLAGLSVEDIAGLGWMVENRLVGVKESLERHHLAAAGAGKQQAGQQQDN<br>VVAPPPALQLQMPPHVSLQPLVPAYGIGGGPTGQTDGVVHHQAPPNPNPQPQPHLQASWL<br>MEVARAGGDLGALVYSGFGGGRGSFGGSAGAGTSTSGAGAADMLPHLGNFGAGFGWPD<br>PAGAGPSSFPPM*
>Sspon.001C0027130	MARKKVTLQWIANDATRRATFKKRRKGLMKKASELATLCDVDACVVVYGEGETQPEVW<br>PDVAKAAEVLARFRAMPELDQCKKMMDMEGFLKQRIDKLKEQLHKARRENHEREVTLLL<br>HDAIVGRRPGLAGLSVEDIAGLGWMVENRLVGVKESLERHHLAAAGAGKQQAGQQQDN<br>VVAPRRSCSCRCLLSIGGGPAGQTDGVVHHQAPPNTNPQPQQASWLMEVARAGGDLGAL<br>VYSGFGGGRGSFGGSAGAGTSTSGAGAADMLPHLGNFGAGFGWPDPAGAGPSSFPPM*
>Sspon.001A0027920	MARKKVTLQWIANDATRRATFKKRRKGLMKKASELATLCDVDACVVVYGEGETQPEVW<br>PDVAKAAEVLARFRAMPELDQCKKMMDMEGFLKQRIDKLKEQLHKARRENHEREVTLLL<br>HDAIVGRRPGLAGLSVEDIAGLGWMVENRLVGVKESLERHHLAAAGAGKQQAGQQQDN

	VIAPPPTLQLQMPPQVSLQPLVPAYSIGGGPAGQTDGVVHHQAPPNLNPQPQPHPQVSWLM<br>EVARAGGDLGALVYSGFGGGRGSFGGSAGAGTSTSGAGAADMLPHLGNFGAGFGWPDPA<br>GAGPSSFPPM*
>Sspon.001B0031732	MARKKVTLQWIANDATRRATFKKRRKGLMKKASELATLCDVDACVVVYGEGETQPEVW<br>PDVAKAAEVLARFRAMPELDQCKKMMDMEGFLKQRIDKLKEQLHKARRENHEREVTLLL<br>HDAIVGRRPGLAGLSVEDIAGLGWMVENRLVGVKESLERHHLAAAGAGKQQAGQQQDN<br>VVAPPPTLQLQMPPQVSLQPLVPAYSIGGGPAGQTDGVVHHQAPPNLNSQPQPHPQVSWL<br>MEVARAGGDLGALVYSGFGGGRGSFGGSAGAGTSTSTSGAGAADMLPHLGNFGAGFGWP<br>DPAGAGPSSFPPIELSTLCDVDACVVVYGEGETQPEVWPDVSKVAQVLARFKAMPELDQC<br>KKTMDMEGLLTQRIDKQKEQLHKAWRENRERETTLLLHDAIVGRRPGLAGLSVEDIAGLG<br>WMVENRLVVVNESLERHHAAAAGAGKQQQDNVVVPPTTLQLQMPQQVSLPLVPPYSIGG<br>PTGGQTAVVHQAPPPNPQPQPHPQASWLMELARAGGDLGALVYSGFGGGRGGCFGGSAG<br>AGTSAGAADMLPHLGNFGAGFGWPDPAGAGPSAFPP
>Sspon.003A0000680	MPRGKIQMSLIGDARDRAKVFARRKAGLVKKAKELTKLCDVDIALVCAGPDGGAPAVWE<br>SDPGVVIERYRRLPADKRAKHTHLDYINGQLGKEERSLDKKRRQGLKALACPGEAVLKDM<br>NLEELLASIDAALLATAERQKALGVADDDGQQLGQQVSSDAVVPVVVGQGDGPPFVGGD<br>DLDDIQAWVDELMWDGAEPLPLNNASMMQPASGVIQYNNGNDLDMVSNHQCQAQMPPG<br>NGENGYGSQLPWHAYQPNTTVSYPDHGFLCTGNNGDMGEYSDMPVPSNANAYDGRYDQ<br>AWWGADEPSCDAPVAVLPAAASYPSLDIAGNPAYMPPQHEHPSMGIGSLMDAGGHEYLV<br>SGCLADYLQCPDASQHFGPLHYLSDSMAQGISYYDDLEAGGCRSGRAQLFAQSHSCSGTP<br>QFSGSEQSQSDVSSGRAQLFNQSHSCCSGTPQFSGQWFREVPVRRGTSVSGYRSTAFLGDV<br>VSGFN*
>Sspon.003B0004530	MPRGKIQMSLIGDARDRAKVFARRKAGLVKKAKELTKLCDVDIALVCAGPDGGAPAVWE<br>SDPGVVIERYRRLPADKRAKHTHLDYINGQLGKEERSLDKKRRQGLKALACPGEAVLKDM<br>NLEELLASIDAALLATAERQKALGVADDDGQQLGQQVSSDAVVPVVVGQGDGPPFVGGD<br>DLDDIQAWVDELMWDGAEPLPLNNASMMQPASGVIQYNNGNDLDMVSNHQCQAQMPPG<br>NGENGYGSQLPWHAYQPNTTICRCRAMPMPTTADQAWWGADEPSCDAPVAVLPAAASYP<br>SLDIAGNPAYMPPQYEHPSMGIGSLMDAGGHEYLVSGCLADYLQCPDASQHFGPLHYLSD<br>SMAQGISYYDDLEAGGCRSGRAQLFAQSHSCSGTPQFSGSGCV
>Sspon.001D0025450	MPRSKIQMSLIGNARDRAKAFARRKAGLLKKAQQLAKLCDVDVALVCAGPDGGAPVVWE<br>SDPGVVIDRYRKLPADKRVKHTHLGYINGQLSKEEGRLDKRRRQGLNKMLACPGEAMLK<br>GMNLEELLASIDAALLATAERQKALGVADDDGQQLGQVSSDVGQGRPPFVGGDELDDIQA<br>WVDELMWDGVEPLPLDASMTTQPASGVIQYINGGNGVDTGSNHQCQAQMLAGNGENGH<br>GQFPWHAYQPNTTVSFPDHSFQCADNNYVDMDEYSEMLMPPGNANAAYESSWDAIAGNP<br>AYNMPPQHEHHSMGIGSFMDAGGHEHETGCLADYIQCPDASQHFGAEPEPLHYLSDMAQ<br>GISYHDDLEAGGCSSGRAQLFAQSHSCSGTPQFSGSEKSQSDVGHQYQDPGVQHFLVMEGP<br>PGSTDVATASVRARGGV*
>Sspon.003C0036783	LHLHTAAANCITSHTFPSLPFLSTTTTSPLIPHPPLLFPFLAPPLTSSFNSLSGSLPAERHCHTTT<br>TMLNMMTDLSCGPSEVTEQPAAPTGSGDKQGRGKIEIKRIENTTNRQVTFCKRRNGLLKKA<br>YELSVLCDAEVALIVFSSRGRLYEYANNSVKSTIERYKKANSDTSNSGTVAEVSA
>Sspon.003C0036750	MQKGNTSNGTMTRRGYDNLRGFYEQHTGLRYDLKCVKNRYSQLKTMYSFYKWALNQTG<br>VGRQANGGLNAPLSWWERHTKKPASNNPSAFDRLVQYMDGTTDLAAPTDLSCGPSEVTE<br>QPAAPTGSGDKQGRGKIEIKRIENTTNRQVTFCKRRNGLLKKAYELSVLCDAEVRSSSSPAA<br>AASTSTQTTGISKTAAAA
>Sspon.003C0025280	MPRGKAGRTSATHAHLAKRKETLRNKAGELVTRCNVDVAVVCTGPGGVGDLDCWPSKA<br>AVDAVVRRYNALEPAERARLKEDLADHVASEVAKEREKLTRTRESGLANAFGSYDGSLQG<br>MSEEKLRELLASIEAALVAARGRVLKLRAPPGGAADRATLSPGLVHDHEAAEEDSASATN<br>NVVPPPTEPPNAVPVDAGEEEVVAENIVTGEDPGDEVQILRPPGDADADDAEWMRSLVDD<br>LKKKPQPHNSAANAAGIEYINVGNSWMERDAYDFIRFDLGMPPPCVAPNYLNDDDDDGEP<br>LELWSWDNTMPPPPK*
>Sspon.003C0006720	MPRGKAGRTSATATATATPHAHLAKRKETLRNKAGELVTRCNVDVAVVCTGPGGVGDLD<br>CWPSKAAVDAVVRRYNALEPAERARLKEDLADHVASEVAKEREKLTRTRESGLANAFGS<br>YDGSLQGMSEEKLRELLASIEAALVAARGRVLKLRAPPGGAADRATLSPGLVHDHEAAEE<br>DSASATNNVVPPPTEPPNAVPVDAGEEEVVAENIVTGEDPGDEVQILRPPGDADADDAEW<br>MRSLVDDLKKKPQPHNSAANAAGIEYINVGNSWMERDAYDFIRFDLGMPPQCVAPNYLN<br>DDDDDGEPLELWSWDSTMPPPPK*
>Sspon.003B0004450	MPRGKAGRTSATHAHLAKRKETLRNKAGELVTRCNVDVAVVCTGPGGVGDLDCWPSKA<br>AVDAVVRRYNALEPAERARLKEDLADHVASEVAKEREKLTRTRESGLANAFGSYDGSLQG<br>MSEEKLRELLASIEAALVAARGRVLKLRAPPGGAADRATLSPGLVHDHEAAEEDSASATN<br>NVVPPPTEPPNAVPVDAGEEEVVAENIVTGEDPGDEVQILRPPGDADADDAEWMRSLVDD<br>LKKKPQPHNSAANAAGIEYINVGNSWMERDAYDFIRFDLGMPPPCVAPNYLNDDDDDGEP<br>LELWSWDNTMPPPPK*
>Sspon.002C0007510	MARGLRGHGTGCPPPRRLLRLLVVPAPRPRPAVIQGPCVRFDAEVANLHTPHLEGIRNPVS<br>RRISIGYSDSPKYGSLRGLGPTLSRACSAPPPSCSLRSVPPIYTDAARPACPAGTRRTAERTQ<br>QGTAERGRGRRALGLIRDRRARVDTFGKRKETLKVKAYELSVLCGVDVALVVAAADGDG<br>GGAAAD
>Sspon.003B0023510	MGLIPNRRLRVSTFGKRKEGLKKKANELSVLCGVEVALVVAPADDGNGGAGVGKADVWE<br>SKEGVLARYRELDPEVRARHTFRDYLYAELGKEEAKLARVRQAGPDGLDCWDKALDGVD<br>TVEEAQKLLDAIDAAIQAADDRRRALGMPIDDDEDGGAGIVLEGIAPLNPAHVDDGYLLHA<br>LGGNVDANDQTMAMWGNNGFHPCSAANMQYGFQQFNSSNVAMEGYDLQMVPDMYNN<br>GGLATDAYHYQTRHAGTMQHGYVFPCARASASYFGMASGYQMQQVVGSGAAQPNLAM<br>SSADEPCHAMVPVPVEYPSADASLNYIDTQATHGVHDGSGGTSFAMDARGNFINAPPAFSV<br>AMSTSGGGGNFISAPPAAPSHTMGGTSDNFTPAQLLAMSYGGDMTVAGRYATQSGIEQLH<br>YLGDLEDTQPNLWGN*
>Sspon.002D0003530	MTGKRRAAERTKQGAAERGRGRSRGRRALGLIRDRRARVDTFGKRKETLKVKAYELSVL<br>CGVDVALVVAAADGDGDGGGAAADVWESTEGAVLARYRALDPEVRARHTHRAYLEGGL<br>GKEEAKLARVRQAGPTGLDPWDKVLDGVATEEEAQGLLEAIDAAIRATEDRMRALGLPVD<br>GEDGVVLEGIAPLAFAGADGYPLHAPGGGGDTYDNQAIWGNDGFETFASQQCTTGSGGAG<br>IEGYHLQITPNMYAAGGSNNNGRLATDDHLDPPRDAGTLRHGYGFHQCAGTDYFGMPAG<br>HQMQELLGWGAAQTNLAMWSTEEPHHAMVPVHYPSAETGLSYMDTPAALGAQGGGCNF<br>VNAPPELSLAMGTAGCVGGGDFINAPPVAFSHAIGGSSDNFTNTTPAQPLAMSYGADLTIA<br>GSRYATQWQAAQQPQRAGRGQQYGVEQLHYLSDLEDAQSQAAQKHQHDGNGPQSGIELL<br>HYLSKLEDTQLHLWGN*
>Sspon.002C0007530	MTGKRRTAERTQQGAAERGRGRGRGRRALGLIRDRRARVDTFGKRKETLKVKAYELSVL<br>CGVDVALVVAAADGDGGGAAADVWESTEGAVLARYRALDPEVRARHTHRAYLEGGLGK<br>EEAKLARVRQAGPTGLDPWDKALDGVATEEEAQGLLEAIDAAIRATEDRMRALGLPVDGE<br>DGVGAGVVLEGIAPLAFAGADGYPLHAPGGGGDTYDNQAIWGNDGFEAFGSQQCTTGSG<br>GAGIEGYHLQITPNMYAAGGSNNNGRLATDDHLYPPRDAGTLRHGYGFHQCAGTDYFGM<br>PAGHQMQELLGWGSAQTNLAMWSTEEPHHAMVPVHYPSAETGLSYMNTPAALGAQGGG<br>CNFVNAPPELSLAMGTAGCVGGGDFINAPPVAFSHAIGGSCDNFTNTTPAQPLAMSYGADL<br>TIAGSRYATQWQAAQQPQRAGRGQQSGVEQLHYLSDLGDAQSQAAQKHQHDGNGPQSGI<br>ELLHYLSKLEDTQLHLWGN*

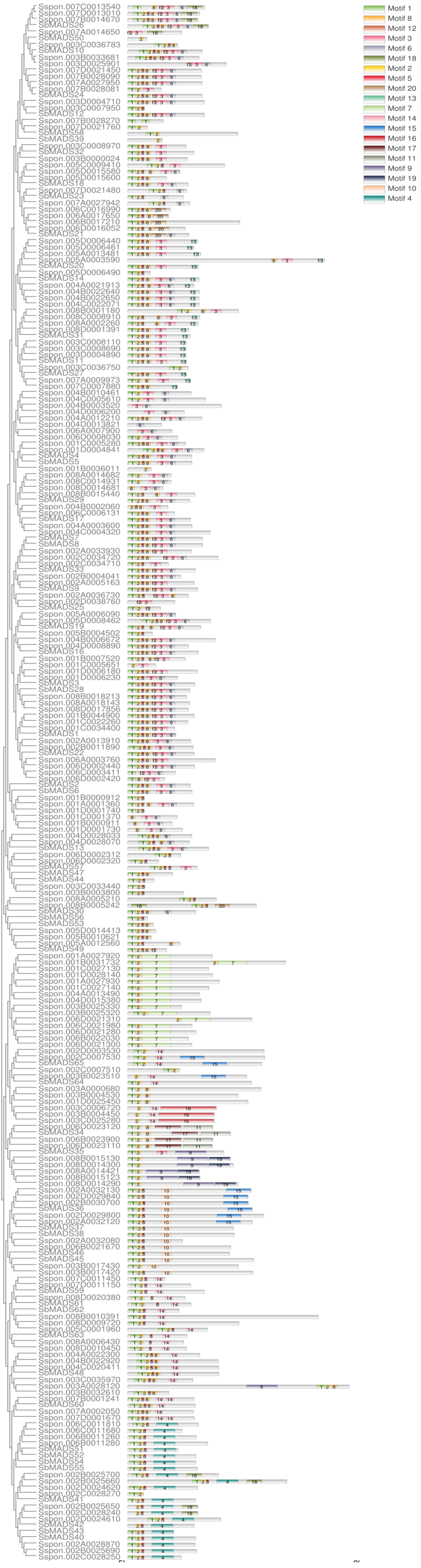

Fig. S1| Motif distribution of 182 *S. spontaneum* MADS-box members, motifs 1,3 and 6 represent MADS-box while 2 represents K-box
